# Mutation rate heterogeneity at the sub-gene scale due to local DNA hypomethylation

**DOI:** 10.1093/nar/gkae252

**Published:** 2024-04-08

**Authors:** David Mas-Ponte, Fran Supek

**Affiliations:** Institute for Research in Biomedicine (IRB Barcelona), The Barcelona Institute of Science and Technology (BIST), 08028 Barcelona, Spain; Institute for Research in Biomedicine (IRB Barcelona), The Barcelona Institute of Science and Technology (BIST), 08028 Barcelona, Spain; Biotech Research and Innovation Centre (BRIC), Faculty of Health and Medical Sciences, University of Copenhagen, 2200 Copenhagen, Denmark; Catalan Institution for Research and Advanced Studies (ICREA), 08010 Barcelona, Spain

## Abstract

Local mutation rates in human are highly heterogeneous, with known variability at the scale of megabase-sized chromosomal domains, and, on the other extreme, at the scale of oligonucleotides. The intermediate, kilobase-scale heterogeneity in mutation risk is less well characterized. Here, by analyzing thousands of somatic genomes, we studied mutation risk gradients along gene bodies, representing a genomic scale spanning roughly 1–10 kb, hypothesizing that different mutational mechanisms are differently distributed across gene segments. The main heterogeneity concerns several kilobases at the transcription start site and further downstream into 5′ ends of gene bodies; these are commonly hypomutated with several mutational signatures, most prominently the ubiquitous C > T changes at CpG dinucleotides. The width and shape of this mutational coldspot at 5′ gene ends is variable across genes, and corresponds to variable interval of lowered DNA methylation depending on gene activity level and regulation. Such hypomutated loci, at 5′ gene ends or elsewhere, correspond to DNA hypomethylation that can associate with various landmarks, including intragenic enhancers, Polycomb-marked regions, or chromatin loop anchor points. Tissue-specific DNA hypomethylation begets tissue-specific local hypomutation. Of note, direction of mutation risk is inverted for AID/APOBEC3 cytosine deaminase activity, whose signatures are enriched in hypomethylated regions.

## Graphical Abstract

**Graphical Abstract:**
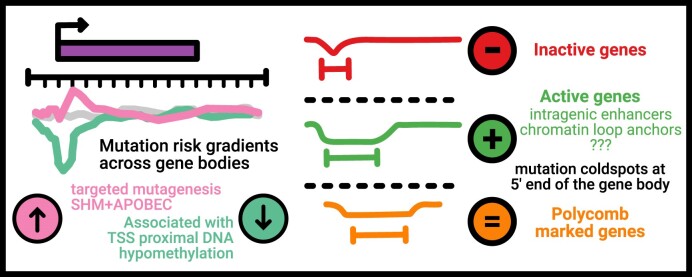

## Introduction

The local variation in mutation rates along the human genome is evident at different scales (1). At the coarse resolution, mutation rates vary substantially across approximately megabase-sized domains (2,3), which correspond to replication timing domains and to topologically-associated domains (4,5). This heterogeneity is generated by the differential activity of DNA repair pathways (6,7), which lower mutation rates in early-replicating, euchromatic domains. At the fine resolution, mutation rates vary strongly according to the trinucleotide sequence, yielding patterns of sequence predisposition termed mutation signatures (8–10); moreover, the pentanucleotide and heptanucleotide sequence neighborhoods predict mutation risk for some processes (11,12). In addition, individual examples of other factors that have an intermediate resolution in the genome can modify the accumulation of mutations by interacting either directly or indirectly with DNA damage and repair processes (1). This includes for instance nucleosome positioning (13), secondary DNA structures (14,15), CTCF (16–18) and ETS transcription factor binding (19–22), and locally open, accessible chromatin (23–25). These individual examples suggest there may be additional, extensive heterogeneity in mutation rates below the domain-scale and above the oligonucleotide scale (the range may be referred to as ‘mesoscale’ (14,26)).

We were motivated to consider mutation rate gradients across gene bodies, because some epigenetic features, such as histone modifications (2) are known to be variably deposited along genes and also known to associate with mutation rates. For instance, H3K36me3 mark is deposited variably along the length the transcribed gene bodies (27). This mark recruits the DNMT3B (28,29) protein, which increases gene body DNA methylation, and also the DNA repair protein MSH6 (30); both factors have potential to affect mutation rates (31). The H3K79 methylation mark was also reported to accumulate differentially across gene bodies (32) and may participate in the repair of breaks, UV damage and the control of error-prone DNA polymerases (33–35), thus with potential to affect mutation rates.

In addition to histone modifications, DNA methylation at the cytosine in CpG dinucleotides (36) is pervasive in the human genome, and is mutagenic since spontaneous deamination directly generates a T/G mismatch (37). A mismatch during DNA replication of the methylated cytosine has also been proposed as a mechanism of mutagenesis (38), particularly upon DNA repair failures (39–41). CpG dinucleotides are variably distributed across the genome, locally accumulating at CpG islands near gene transcription start sites (TSS), which regulate gene transcription via hypomethylation or hypermethylation of promoter-proximal DNA (36). Thus, the enrichment of the highly mutable CpG context near transcription start sites, as well as its variable DNA methylation, also represents a local mutation risk modifier at the gene scale.

Here, we perform a systematic and unbiased analysis of the mutation rate gradients along gene bodies. This revealed a heterogeneity in mutation risk affecting several kilobases at 5′ ends of gene bodies, which are usually hypomutated but this differs across mutational signatures. We highlight the role of local DNA hypomethylation in active genes in generating these intragenic mutation risk gradients, as well as in other functional elements, such as enhancers and chromatin loop anchors, which also correspond to coldspots of certain common mutational processes.

## Materials and methods

### Mutation datasets

Somatic mutations used in this study were compiled from tumor-normal matched whole genome sequencing experiments available by prior studies (Supplementary Table S1). In brief, single nucleotide variants (SNV) from a unified mutation calling dataset comprising the entire Pan-cancer Analysis of Whole Genomes (PCAWG) were downloaded from the PCAWG resource site (https://dcc.icgc.org/releases/PCAWG/Hartwig) with ICGC application code DACO-1101. Somatic SNVs for a set of tumor samples from the Personal Oncogenomics (POG) project were downloaded from BC Cancer, publicly available (www.bcgsc.ca/downloads/POG570). Additional sets of SNVs from healthy tissue samples were compiled from the literature, in particular Yoshida *et al.* Nature 578, 266–272 (2020) and Brunner *et al.* Nature 574, 538–542 (2019).

### Whole genome bisulfite data

Whole genome bisulfite sequencing data (WGBS) were downloaded from publicly available datasets, in brief, the ROADMAP epigenome project (see https://egg2.wustl.edu/roadmap/web_portal/) and the ENCODE data portal (see https://www.encodeproject.org/). From both sites, all available WGBS datasets were first selected and processed but only the ones that were found with sufficient quality and that passed manual inspection were used in the analysis (see Supplementary Methods, Supplementary Figure S15, Supplementary Figure S16 and Supplementary Table S2).

Downloaded data consisted in fractional methylation and coverage files that contains information about the percentage of methylated reads of each sufficiently covered CpG and the original depth of that loci (Supplementary Table S2). Additional sets of precomputed unmethylated and partially methylated genomic loci (UMRs and LMRs, see Supplementary Methods) were also compiled from prior literature (42–44) (Supplementary Table S4).

### Factorization of mutation gradient profiles along genes

Genes were segmented in 250 bp bins along the gene 5′ and 3′ ends together with a central window that extended from the central position of the gene. The gene ends were expanded 2 kb outward (upstream from 5′ end and downstream from 3′ end) and 4Kb inward. The central position was expanded 1 kb in each direction. An extra 1 kb reference bin was selected 4 kb downstream from the 3′ end of the gene. Individual genes were grouped into 3 quantiles according to their average expression in the GTEX V8 portal. Genic bins that overlapped bins from other genes in the same expression quantile were not considered in the analysis. The somatic mutations used in the analysis were first grouped according to the tissue and their assigned signature (Supplementary Methods) and then intersected with the bins across the gene body, yielding a total of 512 sets. The mutational enrichments of each set were measured with a negative binomial regression and the resulting genic bin coefficients factorized in a single PCA.

### Gene classification from DNA methylation profiles

Genes were segmented in 50 bp bins along the 5′ and 3′ ends similarly as above. Both the TSS and the TES ends of genes were extended inward for 5 kb and outward 3 kb. Methylation averages (within 50 bp segments) were computed using deeptools. The resulting matrix contained an average methylation value for each gene and segment. This matrix was factorized using a Principal Component Analysis (PCA) with no scaling (Supplementary Methods). The resulting PC coordinates where then clustered using medoids clustering, selecting five groups from a range of 2–7 after visual inspection of the genomic characterization and methylation gradients (Supplementary Methods). Note that only DNA methylation data was used to group genes. To generate the methylation gradients depicted in this study, each segment in each gene was grouped according to its assigned cluster and the average value per segment was plotted and the 95% (two-tailed) confidence intervals were extracted from a binomial test at a given sample size equal to the number of genes tested. We note that each gene instance was treated independently, thus overlapping regions were included multiple times, once for each gene. These overlaps represented the 5.4% for the region around the TSS and 7.1% for the region around the TES.

### Analysis of alternative epigenetic confounders

To control for potential epigenetic confounders that may overlap with the 5′ gene ends we performed a multivariate analysis that assessed if the inclusion of the confounder mark was sufficient to explain the observed mutation gradient along the 5′ gene end of each methylation-aware gene group. To control for transcription stalling we obtained ChiP-seq data for SPT5 and POL2 in DLD1 cells (45), with GEO codes GSM5170922 and GSM5170919 respectively. To control for premature termination we obtained ChiP-seq data for the protein PCF11 (46) in HeLa cells, GEO code GSM3633288. To control for nucleosome positioning around the TSS we included direct Mnase readouts from the ENCODE dataset ENCSR000CXP, measured in the GM12878 cell line and MNase accessibility data (47) (MACC) for the K562 cell line with GEO code GSE78984. To control for transcription factor binding sites (TFBS) we obtained a dataset of both active and inactive loci from ref (21).

### Analysis of sex specific mutation rate in chromosome X

We downloaded the sex of the donor information for our PCAWG from the clinical and histology section in the data portal (https://dcc.icgc.org/releases/PCAWG/clinical_and_histology). The resulting dataset comprised 994 male and 819 female samples. We obtained CpG islands from the UCSC Table Browser and promoters from the UCSC bioconductor package. We assigned a expression quantile to each locus based on the average gene expression value of the closest gene. Using a negative binomial regression, we compared the mutation rate of both promoters and CpG islands against a reference locus located 10Kb upstream, thus maintaining the overall replication time domain. We performed a single independent regression for each combination of chromosomes, group of samples (male or female), selected signature (1, 2, 7a, 7b, 10a, 10b, 13 and 15) and loci type (either promoters or CpG islands in both expression quantiles).

### Selection analysis

Selection in genes was estimated from whole exome data available from the mc3-TCGA dataset (Supplementary Methods). For each gene we mapped the gene id to the epigenomic-based PC coordinates of dNdScv package to use as a base model in our predictions. We also extracted the trinucleotide composition of each gene and normalized them per gene. A negative binomial regression was used to predict the total number of mutations observed at a specific gene (the mutation burden). This approach was repeated three times, (i) with a base model containing the size of the gene, the trinucleotide composition and the dNdScv covariates, (ii) with the base model and the methylation aware gene grouping and (iii) with the base model and a shuffled set of methylation groups that maintained the existing class imbalances of the original classification (Supplementary Methods). Cancer associated genes were downloaded from MutPanning dataset, with the added requirement that they must be associated with at least two cancer types, and they were excluded from the initial fitting of the model.

## Results

### Sub-gene mutation rate gradients are observed in DNA methylation-associated mutational signatures

**Figure 1.**
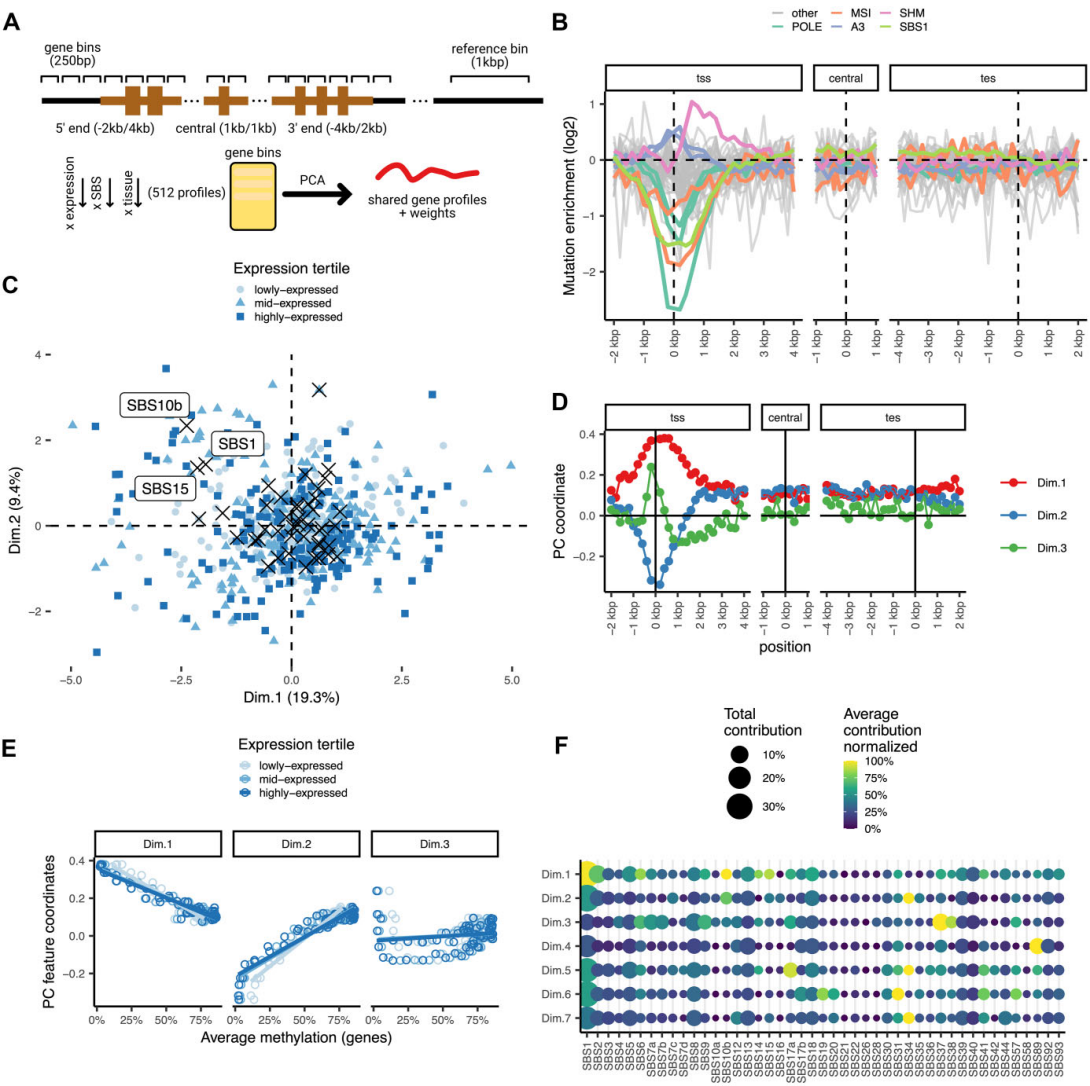
Systematic quantification of mutation gradients along gene bodies. (**A**) Diagram of the analysis of mutation rate gradients process and posterior factorization with a PCA. The genes are divided in 250bp long bins for which the mutation rate is calculated. The mutation rates at each bin is measured with a negative binomial regression and the output is factorized using a PCA. (**B**) Mutation rate estimates for different mutational signatures in a pan-cancer setting. Gene regions defined as 250 bp bins across extended regions near both genic ends and the central position. (**C**) PCA coordinates of the instances included in the regression, here 512 points representing each combination of expression bin, signature and tissue of origin. Crosses in black represent the average of all points of each mutational signature. (**D**) Profile weights of the three principal component along the gene body. (**E**) Correlation of median methylation levels across genes against coordinates of component 1, 2 and 3. (**F**) Contribution of each mutational signature to each component. In color the average contribution within the tissues that contain a certain mutational signature is represented while the size represents the total contribution against all cancer types.

To systematically study the sub-gene scale variability of local mutation rates, we analyzed a set of 2782 tumor and healthy somatic whole-genome sequences (see Materials and Methods and Supplementary Table S1), mainly from the PCAWG dataset (48) and other sources (49–51), to calculate the relative mutation enrichment at various loci across the length of gene bodies. In brief, we separated mutations by the mutational signature that likely generated them (see Supplementary Methods), and binned the gene body into 250 bp long segments; these covered two extended regions (each 6 kb long) along the 5′ and the 3′ gene ends, and additionally they covered a central region (2 kb long). Genes were pooled for this analysis and stratified into three bins by mRNA expression levels (average across various tumor types, see Supplementary Methods). We estimated the local relative mutation rate, controlling for the trinucleotide composition of each 250 bp segment, by using an approach based on negative binomial regression (see Supplementary Methods and Figure 1A).

The pan-cancer mutation rate profiles, stratified by signature (averaged over gene expression bins, Figure 1B and Supplementary Figure S1) revealed a strong and consistent mutation rate depletion at the 5′ gene end, occurring mainly for the following signatures: SBS1, associated with the deamination of methylated cytosines; SBS15 and SBS6, associated with MMR deficiencies (microsatellite instability, MSI), and SBS10a, SBS10b and SBS28, associated with mutations in the proofreading domain of the replicative DNA polymerase ϵ (*POLE*) (52,53). In contrast to this depletion, the APOBEC-associated mutation signatures, SBS2 and SBS13, showed an increase in the region surrounding the transcription start site (TSS). As a positive control, we observed that SBS9, which associates with somatic hypermutation (SHM) process in lymphoma, localized at active promoters (Figure 1B) as expected (54). However, when considering the central section of the gene or the 3′ gene ends, in most mutational signatures no clear trends were observed. Overall, in a pan-cancer setting, most of the heterogeneity in mutation risk at the sub-gene resolution results from gradients at the 5′ gene end.

To more rigorously study these trends and their association with tissue and with gene activity levels, we next extracted the dominant patterns in mutation enrichment along the selected sub-regions of the gene bodies using a principal component (PC) analysis (Figure 1A,C); here, the mutation risk was further divided by tissue-of-origin to confirm that trends are consistent across cancer types (Supplementary Figure S2). The first PC accounted for ∼19% of the systematic variability in trinucleotide-adjusted local mutation rates (Supplementary Figure S3A). Its profile along the gene body plateaued at the TSS and the 1 kb downstream thereof, and continued in the downstream regions, with some of the altered mutation risk apparent up to 3kb downstream of TSS (Figure 1D). The second component (PC2), explaining ∼9% variability in local mutation risk (Supplementary Figure S3A), showed a narrower peak at the TSS (note that the direction of PCs is arbitrarily assigned by PCA; Figure 1D). We interpret these first two PCs jointly: their combination describes the variation in the width and/or intensity of the 5′ hypomutated region across genes and/or mutation signatures. This PC analysis confirms that most of the variability in mutation rates within genes accumulates at the TSS and further downstream into the 5′ ends of gene bodies, however, it does also suggest that additional, quantitatively minor, trends of mutation risk heterogeneity within gene bodies may exist (see below).

The PC1 of the local mutation rates is characterized by a lowered burden mainly from mutational signatures SBS1, SBS15 and SBS10b (Figure 1A, C). Each of these signatures contains a significant NCG > T component in its trinucleotide spectrum, suggesting an association with local DNA methylation (Figure 1C and Supplementary Figure S3B–D). Moreover, signature SBS1 is considered a stereotypical mutational process arising at methylated cytosines in a CpG context, presumably due to their spontaneous deamination (36,37). This localization strongly correlates with the average DNA methylation levels at those positions (Figure 1E and Supplementary Figure S3B). An association with DNA methylation also fits with the difference we observed between gene expression bins, where higher expressed genes (expected to have lower DNA methylation at the promoter/TSS) showed higher weights of PC1 in the SBS1 and in the other NCG-rich SBS mutational signatures (Figure 1C and Supplementary Figure S3D).

The third local mutation rate trend, PC3, explains less variance (∼3%), (Figure 1D–F and Supplementary Figure S3E) and its profile did not correlate with local DNA methylation (Supplementary Figure S3B). Instead, PC3 was characterized by a sharp, hotspot-like mutation risk increase directly upstream of the TSS observed in skin, gastric and lymphoid cancers (Supplementary Figure S3E and Supplementary Figure S4); this is consistent with the known promoter hotspots due to UV damage in skin (19,20), with CTCF-binding site hotspots associated with SBS17 in gastrointestinal cancers (16), as well as with local SHM in lymphocytes (54).

Overall, the trends in local mutation risk at the gene scale that we identified via PC1 and PC2 explain considerably more variation than the known promoter-associated hypermutation processes jointly summarized in PC3. Further PCs, PC4 and PC5, showed less clear patterns across the gene body (Supplementary Figure S3F).

To aid interpretation, we further applied a sparse PCA (see Supplementary Methods), which recapitulated the mutation rate variation near the TSS, and independently a type of variation that extends within the gene body for at least 2 kb (Supplementary Figure S3H, I).

Overall, our analysis identified mutation rate variability along human gene bodies in multiple tissues and across gene expression levels. There is a substantial reduction of mutation rates at the TSS and extending downstream into 5′ ends of gene bodies, with a plausible role of local DNA hypomethylation in shaping this intragenic mutation risk variation.

### Gene stratification by local DNA methylation profile reveals epigenomic signature of intragenic enhancers

**Figure 2.**
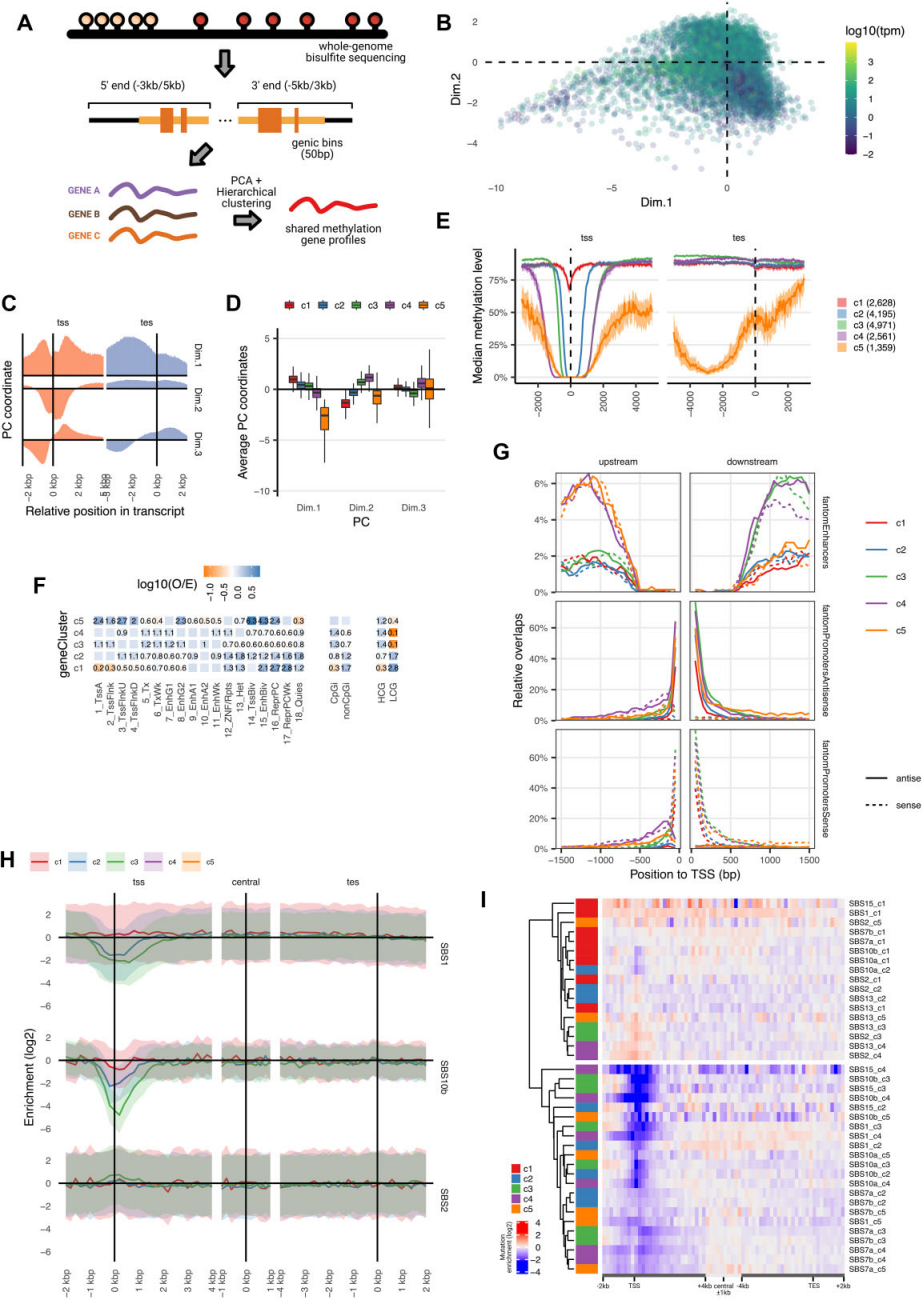
Clustering of genes according to their methylation profile. (**A**) Diagram of the gene clustering based on methylation gradients. Genes are tiled with 50bp bins and the average methylation value is calculated per bin. Gradients are then put together into a PCA and clustered using hierarchal clustering. (**B**) PCA coordinates of each gene from the factorization of methylation profiles. Color represents average gene expression across tissues. (**C**) PCA weights for the three first components used in the clustering of the methylation profiles. (**D**) PCA coordinate distribution of each gene cluster for the first three principal components. (**E**) Median methylation level for all genes in each cluster. Area represent the 95% confidence interval of the median across all genes in each group. (**F**) Overlap enrichment measured from observed and expected counts from a contingency table. Significant values are shown as numbers. Colors represent the logarithm in base 10 of the O/E score. Numeric values represent the untransformed O/E value. (**G**) Profiles of transcriptional data measured by CAGE from the FANTOM consortia(56) and subdivided across promoters, enhancers and sense and antisense transcription (**H**) Mutation rate gradients of a selected set of mutational signatures (SBS1, SBS10b and SBS2) in a selected set of methylation profile gene groups. (**I**) Same as in (h) but with all gene groups for a wider set of signatures.

We hypothesized that different groups of genes, for instance due to varying expression level (36,55), would show distinct shapes of local DNA methylation profiles and thus also varying shapes of mutation risk gradients along their gene body. To test this, we used the whole genome bisulfite sequencing (WGBS) DNA methylation data averaged along multiple solid tissues (see Materials and Methods and Supplementary Table S2) to profile the local DNA methylation levels. In brief, gene bodies were segmented into 50 bp bins, extending from the TSS and the TES inwards (i.e. towards gene center) by 5 kb, and extending outward (i.e. to gene flanking regions) by 3 kb, and the DNA methylation level was averaged within every bin. The resulting profiles were then factorized using a PCA (see Materials and Methods, Figure 2A and Supplementary Figure S5A), generating DNA methylation profile PCs (mePCs) that, expectedly, correlated with gene expression (Figure 2B and Supplementary Figure S5B). The first three mePCs, representing the DNA methylation levels globally in the whole gene body (mePC1), the local TSS-proximal hypomethylation extending downstream into the gene body (mePC2) and its shift towards more upstream versus downstream location (mePC3) (Figure 2C). These were used to cluster human genes into 5 groups, based on the shape of their local DNA hypomethylation profiles (Figure 2D, E and Supplementary Table S3).

While our main interest was to correlate these methylation profiles within genes with intragenic mutation rate profiles, we also briefly considered their various epigenetic and regulatory associations (Figure 2F and Supplementary Figure S5C, D). The group c1, and to some extent group c2, contained genes with a methylated promoter (Figure 2E), lower expression levels (Supplementary Figure S5D) and a depletion in active transcription chromatin states (see Supplementary Methods and Figure 2F). Content of CpG islands (57) and CpG-rich promoters (58) was low in c1 and c2 (Supplementary Figure S5C). The differentiating factor of c1 versus c2 was that c2 genes show some DNA hypomethylation at promoters (Figure 2E), and the c2 chromatin states having some features of activity (Figure 2F). In contrast, gene methylation group c3 and c4 each represent a set of active, expressed genes (Supplementary Figure S5D) with a wider hypomethylated section that includes the promoter. Importantly, we note also that this hypomethylation extends into the 5′ end of the gene body, approximately 2 kb downstream of TSS, for both the groups c3 and c4 (Figure 2E). While hypomethylation of DNA at active gene promoters is well-known, here we note the hypomethylated regions extending downstream into human gene bodies is a hallmark of gene activity.

Next, we asked what could underlie this lowly methylated region downstream of the TSS. Because these gene groups c3 and c4 show overlap with active enhancer chromatin states (Figure 2F), we hypothesized that the activity of intragenic enhancers could be one mechanism generating local hypomethylation within gene bodies. To test this, we analyzed nascent RNA transcription (measured by Cap Analysis of Gene Expression, CAGE) (56,59,60)), which is indicative of enhancer activity. This has indeed shown CAGE signal in the gene body hypomethylated sections of the c3 and c4 active genes (Figure 2G). Of note, transcription was detected in both DNA strands (Figure 2G, dashed versus full lines), which is consistent with the activity of an enhancer (60) within the gene body. The main difference between c3 and c4 groups is the extent of the 5′ unmethylated section upstream of TSS: c3 only presents the hypomethylation downstream of TSS into the gene body, while c4 has an extended hypomethylated region both upstream and downstream with similar length (Figure 2E). Encouragingly, the enhancer RNA transcription follows the same positioning, with c3 showing higher CAGE signal only in the TSS downstream section of the 5′ gene body, but c4 in both directions away from TSS (Figure 2G). Further characterization of the methylation differences between the gene groups revealed that the CpG island shores (61), i.e. regions adjacent to the island, could broadly account for these differences between c3 and c4 group (Supplementary Figure S5E).

The gene group 5 (c5) contained generally shorter genes (Supplementary Figure S5D) with an overall less methylated gene body across its whole length (Figure 2E and Supplementary Figure S5). These genes overlapped with repressed TSS and enhancer states (Figure 2F) and were, consistently, enriched with the Polycomb silencing histone mark H3K27me3 (Supplementary Figure S5G). Many of the homeobox genes, developmental genes with roles in cancer that were reported to be hypomethylated (42), were included in this group (representing ∼7% of the c5 genes, Supplementary Figure S5F). The Polycomb repressive mark was previously associated with longer DNA hypomethylated segments referred to ‘canyons’ and ‘valleys’ (62,63), consistent with patterns observed in our gene group c5.

The averaged histone profiles of each gene category (Supplementary Figure S5G) supported the mechanism where hypomethylation within gene bodies is causally linked to active intragenic enhancers: in a regression analysis, the active transcription initiation features however also enhancer-like chromatin features (the H3K4me1 histone mark within the gene body) were particularly relevant for the distinction of the gene groups with different DNA methylation profiles (Supplementary Figure S5H). Consistent with the nascent RNA transcription described above (via CAGE), the existence of intragenic enhancers thus explains some cases of the hypomethylated region extending downstream of the TSS, several kb into the 5′ part of the gene body. We note that this represents an average trend, but in individual genes intragenic enhancers may be located at various positions. Reported associations of higher gene expression with lower DNA methylation in the first exon, as well as patterns of evolutionary conservation, are consistent with a preferential 5′ gene body placement of intragenic enhancers (64,65).

### Sub-gene scale mutation rate gradients differ across DNA methylation-based gene groups

By considering grouped genes by local DNA methylation profiles, we aimed to ascertain the role of DNA methylation changes in the local, sub-gene mutation rate gradients. In other words, we asked if the intragenic mutation risk gradients change between genes as the intragenic DNA methylation gradients change, supporting the causal role of DNA methylation in mutation risk for each mutational mechanism. The genes having DNA hypomethylation at and/or nearby their promoters—to some extent those in groups c2, and more prominently the active c3 and c4—showed a stronger depletion of mutational burden at 5′ gene ends, mainly SBS1, SBS10b and SBS15 (Figure 2H and Supplementary Figure S6). Conversely, those mutation signatures that were enriched at the 5′ gene end, APOBEC SBS2 and SBS13 and the AID-associated SBS9, showed an increased rate around promoters in gene clusters c3 and c4 (Figure 1B, Figure 2H, I). In contrast, in the lowly active c1 gene group the local mutation rate was less variable across gene bodies, in accord with the rather homogeneous DNA methylation levels across their 5′ gene part (Figure 2I). Genes in the cluster 5 (c5) group presented a less localized hypomutation throughout the gene body, consistent with the hypomethylation of the whole gene locus.

Further, we considered a possible association between these gradients in DNA methylation, and the mutation risk in comparing exonic *versus* intronic DNA, in light of reports of subtly different mutation rates (66–68) and subtly different DNA methylation in exons versus introns (69,70). We checked the DNA methylation level of exons and introns, separately for each exon/intron in sequence, for a representative gene set (middle tertile of genes by length, and middle tertile in expression level). While methylation in the first exon was substantially lower compared to the first intron, consistent with the exon's more 5′ positioning, the DNA methylation levels across the subsequent introns and exons were highly similar (Supplementary Figure S7). Thus, in human WGBS data, after accounting for 5′ gene end hypomethylation, we see no notably different DNA methylation in the exonic versus intronic loci, and if there are any differences between introns and exons in mutation rates, these do not stem from different DNA methylation.

Overall, we see evidence that the main determinant of the local mutation rate heterogeneity along human gene bodies is local DNA methylation, which however has different manifestations in different genes and mutational processes. Genes that are more highly expressed have more prominent and wider mutational coldspots toward their 5′ ends, when considering common mutational processes such as aging-associated SBS1, and some DNA repair failures (SBS15 and SBS10b). These trends are however reversed for APOBEC/AID mutagenic signatures, which are enriched at hypomethylated DNA found at active promoters and intragenic enhancers.

### Lowly methylated regions within or outside genes show consistent hypomutation

**Figure 3.**
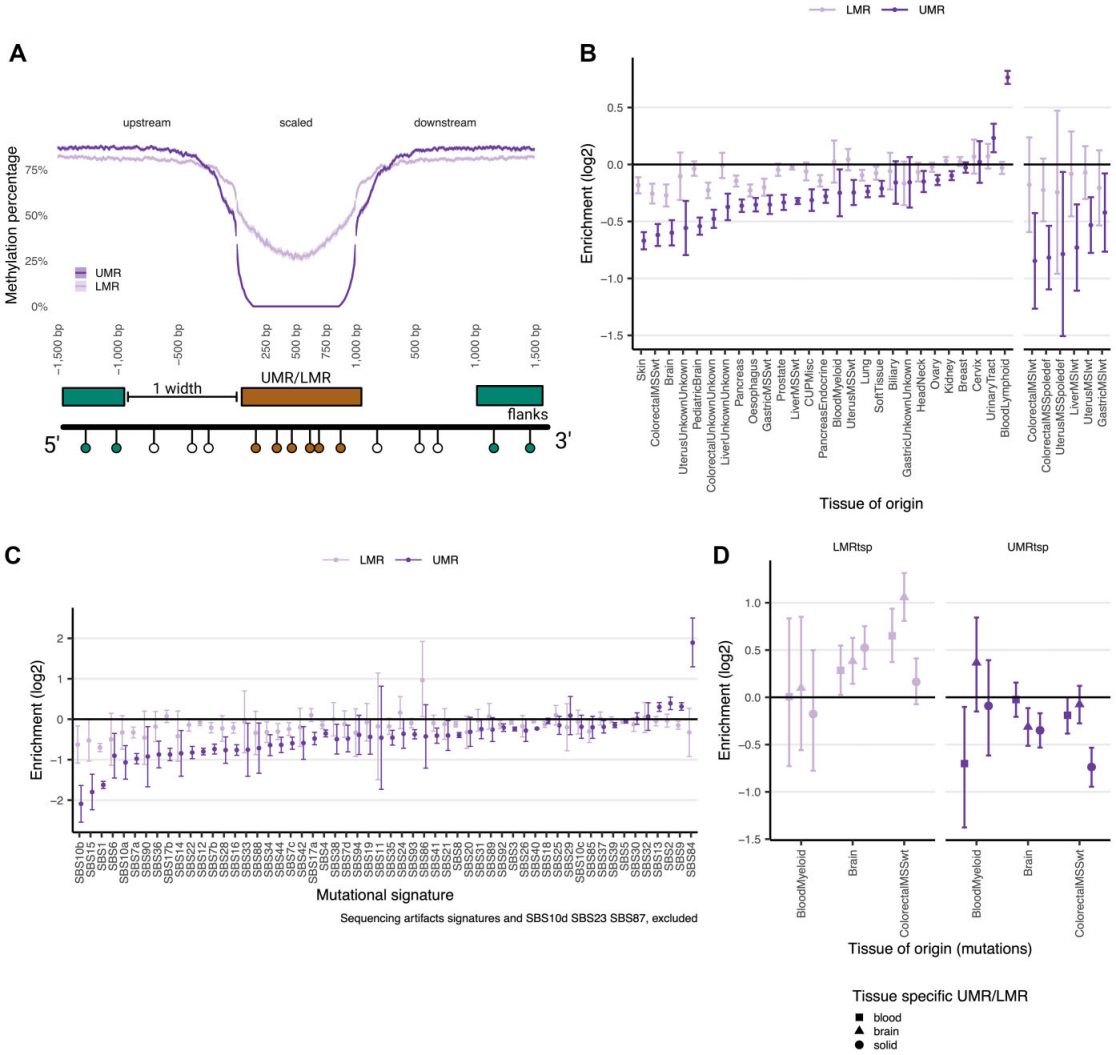
Mutation rate gradients in genome-wide hypomethylated regions. (**A**) Methylation profiles, measured as the median methylation level in each bin, for both UMRs and LMRs. Shadow area represents the 95% confidence interval of the median value across all regions. (**B**) Coefficients representing the relative mutation rate change for the UMR or LMR regions versus flanks. Each regression includes all mutations for a given tissue. (**C**) Same as in e but for the assigned mutational signatures. (**D**) Coefficients measuring the relative mutation rate change in tissue specific UMRs and LMRs versus flanks.

Following up on the analysis of gene body mutation rates, we hypothesized that DNA methylation has roles in shaping local mutation rates in various loci across the genome, even outside of genes. To test this, we collated a genome-wide dataset of loci that are hypomethylated in various cell types, either with a near-complete lack of DNA methylation, unmethylated regions (UMRs), or in lowly-methylated regions (LMR), with intermediate methylation levels (43,44) (Figure 3A). These segments contain a high CpG dinucleotide density and were reported to reflect the functional regulatory elements, including both promoters and enhancers (43).

In addition to hypomethylated regions from previous publications (42,44) (Supplementary Table S4), we also collected genome-wide (WGBS) DNA methylation data from Roadmap (71) and Encode (72), and called UMR and LMR loci therein using MethylSeeker (44) (see Supplementary Methods and Supplementary Table S2). Here, we considered 34 diverse solid tissues (non-neural) plus 6 blood, and 4 brain tissues, a selection that represents the tumors we analyzed (see Supplementary Methods; the solid, blood and brain tissue groups are treated separately because of considerable differences of epigenomes between them (72)). In total, all obtained UMRs spanned 41Mbp, similar to previous reports, while LMRs detected in this study covered a total of 25 Mb (Supplementary Figure S8A, B).

Most of the surveyed tissues, except the urinary tract cancers (often highly mutagenized by APOBEC) and the lymphatic blood cancers (often showing somatic hypermutation via AID), showed lower mutation rates at UMRs and LMRs, with an average depletion of ∼25% (Figure 3B). This local hypomutation was more substantial for tissues with a high proportion of SBS1 mutations, such as colon and brain (8), and was also notable in skin, consistent with a proposed role of DNA methylation predisposing to UV damage mutations (Figure 3B) (73). These associations were highly consistent when tested on either the UMRs obtained from previous publications, or the UMRs we identified in this study (Supplementary Figure S8C).

Considering individual mutational signatures, SBS1, 10b and 15 decreased the most in genome-wide UMRs, which mirrors our analyses of hypomethylated 5′ gene ends above. UMRs had on average ∼75%, ∼65% and ∼55% fewer mutations than expected by trinucleotide composition, for SBS10b, 1 and 15, respectively. Other mutational signatures like SBS6, related to MMR deficiency, and SBS7a resulting from UV exposure also showed a reduction at UMRs (Figure 3C).

Again consistently with the gene body analysis, certain signatures showed an increased mutation rate at UMRs genome-wide. SBS84, associated with AID activity in the SHM process (74) in lymphocytes showed a four-fold (2.5–5.6-fold; 95% CI) increase. Consistently, SBS9, also associated with SHM in lymphoid tissues, in part reflecting the activity of DNA polymerase η, did so as well to a lesser extent (29%). Finally, the widespread signatures SBS2 and SBS13 resulting from APOBEC mutagenesis also showed an enrichment in the genome-wide UMRs, with a 35% increase over expected mutation rates for SBS2 and 28% for SBS13 (Figure 3C). Thus, AID (expectedly) and interestingly also APOBECs preferentially mutate hypomethylated DNA, which is common at active gene regulatory elements.

### Tissue-specificity in DNA hypomethylation-associated mutational coldspots

We considered the differences in local DNA hypomethylation between tissues, testing association with local mutagenesis in tumors occurring in the matched *versus* the mismatched tissues, to provide evidence for causal roles of DNA methylation in mutation rate changes. As representatives from the solid tissue group, we chose three datasets from the digestive tract, comparing them with the colorectal cancer mutations. Of note, LMRs were more tissue specific than UMRs (Supplementary Figure S8D, E), consistent with the LMR-associated genomic features, i.e. enhancers (43). The total yield of tissue-specific UMRs was thus sparse (Supplementary Figure S8E).

Indeed, the depletion of mutations in UMRs from the matching tissue was more evident than in the UMRs of the mismatched tissues. For instance, colon cancers showed a 40% lower mutation rate for UMRs specific to digestive tissues, while the reduction of mutations in brain and blood cancers at digestive tissue UMRs was not as substantial. Similarly, the reduction of mutation rates in the blood-specific UMRs was 37% (7–61%, 95% CI) in myeloid blood tumors but only 4% (−30% to +35%, 95% CI) for colon cancers. Our brain-enriched UMRs showed a less striking selectivity (Figure 3D).

Overall, the variability in local mutation rates at UMRs at the tissue level is explained both by the signatures the tissue is normally exposed to, and additionally by the tissue-specific variation in hypomethylated loci.

### Variable mutation rates at UMRs that intersect important functional genomic elements

**Figure 4.**
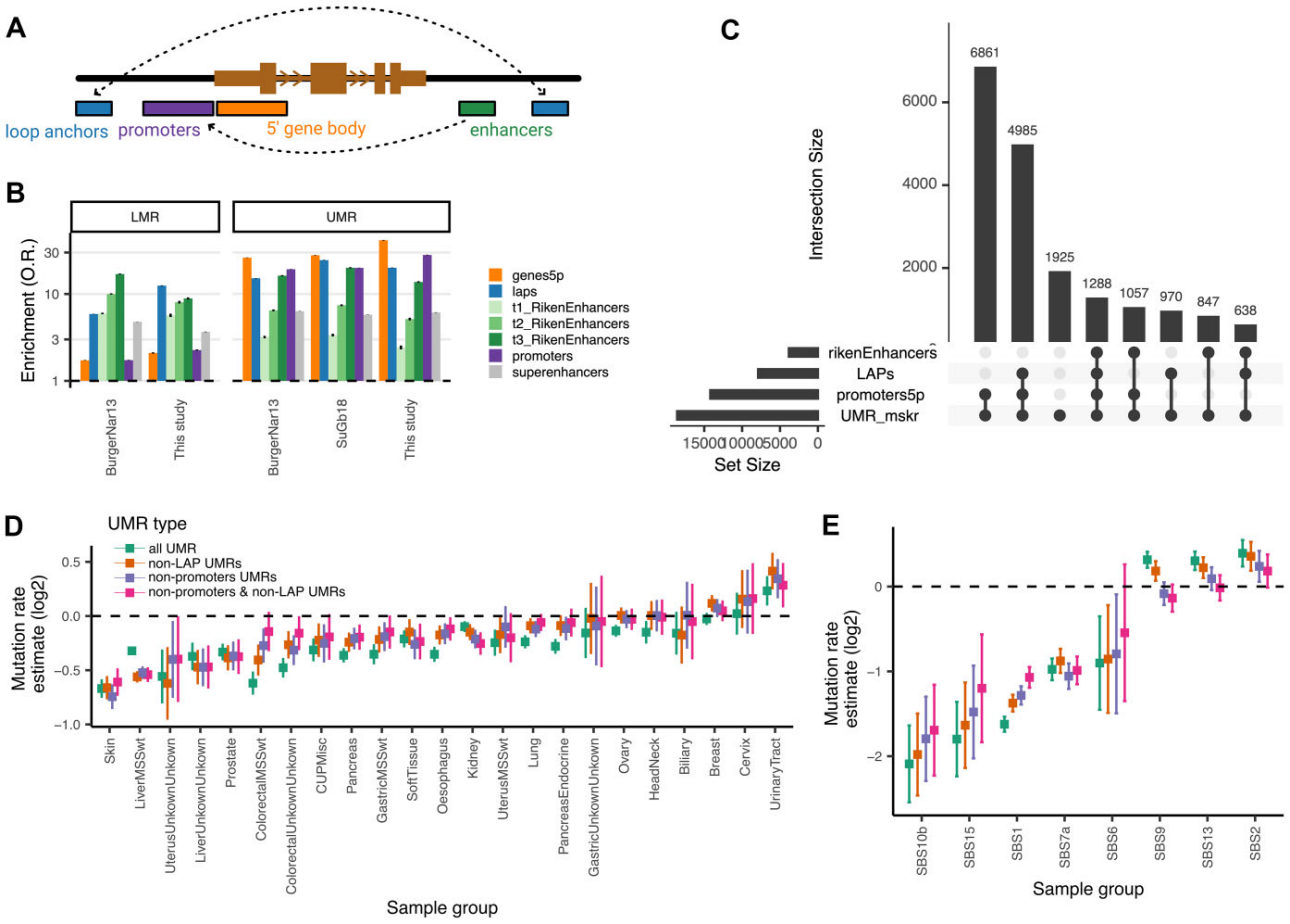
UMRs and interactions with other functional elements. (**A**) Diagram of the set of the relevant functional elements represented here. (**B**) Odds ratio enrichment of the overlap of a given functional element either with the UMR or the LMR. (**C**) Upset plot showing all possible intersections of UMRs with the functional elements depicted in (A). In this panel, the 5′ end of the gene body and the promoter is mixed in a single group. (**D**) Mutation rate enrichment for UMRs that do not present an overlap with functional promoters or loop anchors. (**E**) Same as in (D) but for the stratified mutational signatures.

Due to the characteristic hypomethylation of regulatory elements like promoters, enhancers, and CTCF/cohesin binding sites (75–78) (here, we considered chromatin loop anchor points from cohesin ChIA-PET experiments (79)) we used these annotations to classify the extracted UMR sets. This was to ask whether the methylation effect on mutation rate is particular to some functional elements or is a general property of DNA hypomethylation seen in every type of element and also seen outside known functional elements (Figure 4A).

As expected, UMRs were enriched in promoters as defined at the TSS upstream region, and LMRs in enhancers as defined by CAGE experiments from FANTOM (56) (Figure 4B). This held true for both prior UMR sets and the UMRs called in this study. The highest number of UMRs was explained by promoters and the promoter-adjacent 5′ ends of gene bodies (37% uniquely, and 76% explained together with other elements; (Figure 4C)). However, 10% of the UMRs genome-wide did not overlap with these known elements (Figure 4C) and for LMRs, this was up to 52% (Supplementary Figure S9A), broadly consistent with previous estimates of UMR and LMR overlap with regulatory elements (43).

We then asked if the local reduction in mutation rate may have resulted from some other property of these functional elements (promoters, enhancers or CTCF/cohesin-bound loop anchors), rather than necessarily their DNA hypomethylation itself. For every tissue, we removed the UMRs that overlapped either the promoter-adjacent region or 5′ gene body region (2 kb downstream and 1 kb upstream of TSS), or chromatin loop anchors. (Enhancers were not considered here because of little overlap with UMRs). The overall trend of hypomutation was still evident in remaining UMRs, both across tissues (Figure 4D and Supplementary Figure S9B) and signatures (Figure 4E), albeit at somewhat lower magnitude. SBS1 was hypomutated 69% in the full UMR set, and hypomutated to a similar level (51%) in the UMRs not overlapping promoters or cohesin loop anchors; SBS15 depletion was reduced slightly from 70% (all UMRs) to 54% (UMRs outside known elements). We note that the UMR sets that overlap with promoters and loop anchor points did show a slightly stronger hypomethylation (Supplementary Figure S9C).

Thus the mutational effect at hypomethylated DNA loci is largely independent of their overlap with known functional elements. This implies that some additional mutation coldspots resulting from hypomethylation can occur elsewhere in the genome, unrelated with known promoters or enhancers or loop anchors.

### Hypomutation of genes and regulatory elements is largely explained by hypomethylation thereof

The above analysis suggests that hypomethylation is causal to local mutation rate reduction at functional elements genome-wide. To further examine if hypomethylation is the main determinant of local mutation risk in regulatory DNA, we examined if the promoters and the CTCF/cohesin-bound chromatin loop anchors exhibit any additional mutation rate reduction, after accounting for their hypomethylation.

As anticipated from their overlap with identified UMRs, the median methylation value around promoters as well as CTCF/cohesin chromatin loop anchors shows a substantial depletion (Supplementary Figure S9D, E) of the average methylation values.

While the hypomethylation of CTCF bound sites is known (55,76), we note that in the broader region containing the chromatin loop anchors (here from cohesin ChIA-PET (79)), the methylation levels were reduced, meaning hypomethylation extends further than the CTCF binding site. This hypomethylation at loop anchors is consistent with the overlap between promoters, UMRs and CTCF binding sites (Supplementary Figure S9E), but appears not fully explained thereby. The above suggests that chromatin loop anchor regions are hypomethylated, regardless of their spanning promoters or included CTCF sites.

The mutation rate (relative to flanking DNA) was reduced at both promoters and chromatin loop anchors, and importantly this reduction depended on DNA hypomethylation (Supplementary Figure S9F). SBS1 mutations showed an overall 51% depletion in all chromatin loop anchors, while showing a lesser depletion in those loop anchors that did not contain a detected UMR, and a similar depletion was also seen in other methylation-associated signatures like SBS15 and SBS10b. Of note, the reduction of mutations in neither promoters nor chromatin loop anchors was as striking as when measuring the UMR itself, suggesting that other known factors at these regions such as chromatin accessibility (23) may further modulate local mutation rates therein (24).

Nucleosome occupancy and binding of transcription factors (TFs) may affect the mutation rates of some mutagenic processes (21,80); these factors may also be differentially positioned at 5′ gene ends (81). Therefore we investigated whether the binding of TFs and nucleosomes may be responsible for the 5′ gene end mutation rate gradients. To test this, we performed a regression stratified by TF binding (data from (21)) and nucleosome occupancy (data from ref (47)), thereby adjusting for possible confounding of these factors. These analyses revealed that TF and nucleosome positioning, while they may influence mutagenesis as anticipated, are not the underlying cause of the 5′ gene end hypomutation by SBS1/10/15 nor hypermutation by APOBEC signatures, since these associations largely remain significant after controlling for TF and nucleosomes (Supplementary Figures S10, S11 and Supplementary Table S5).

To rule out that the effect of the UMR on mutation rates was indirect and resulted from the increased transcription of genes with a UMR, we repeated this analysis after stratifying genes by mRNA expression (Supplementary Figure S9G). For colorectal and skin tissues, which contained sufficient mutation counts, the local mutation rate in genes with high mRNA expression but without overlapping UMR was less reduced, suggesting transcription levels do not explain the mutation rate decrease. The relative mutation rate of gene-overlapping or adjacent UMRs was notably reduced compared to their UMR-less counterparts of same expression level (Eq2 and Eq3 bins, Supplementary Figure S9G). Of note, some tissues like liver did show reduction of mutation rate in higher expression bins, consistent with transcription-coupled processes, however even so, mutation rates were still reduced in UMR-overlapping promoters (Supplementary Figure S9G).

To further consider the possible effects of transcription dynamics within genes upon intragenic mutation rate heterogeneity, we tested if changes in mutation rates at 5′ ends of gene bodies remain significant after adjusting for RNA polymerase II occupancy (45), binding sites of the SPT5 stalling factor (45), and the binding sites PCF11 transcription termination factor (46). The mutation rates shown for individual signatures we found depleted (SBS1, SBS10, SBS15, SBS7) or enriched (SBS2, SBS13) without or with adjustment are shown in Supplementary Figures S10, S11. Overall, our main results concerning mutation rate heterogeneity at 5′ ends of gene bodies cannot be explained by dynamics of RNA polymerase II during transcription, since the associations largely remain significant in the active gene groups (the ‘promoterDown’ region in the c3 and c4 groups, Supplementary Figure S11). However, these results also suggest that RNA polymerase II dynamics might have additional effects on mutation rates for certain signatures.

We further considered a global epigenetic modification that is related to CpG methylation at many sites—the inactivation of chromosome X in females (XCI) (82,83). Our various analyses normally considered only autosomal genes (as per our definition of the methylation-aware gene groups; Supplementary Table S1, Supplementary Figure S12A, Supplementary Methods). Here, we surveyed specifically the chromosome X, in terms of relative SBS1 mutation rates at CpG islands and 5′ gene ends (here defined as 1Kbp downstream and upstream of the TSS), contrasting lowly and highly expressed genes. We compared male samples, which do not undergo X inactivation, and female samples, which do (Supplementary Figure S12). We found a clearly increased mutation risk at the promoters of higher-expressed genes in female samples, compared to the chrX of male samples, and the autosomes, both in females and in males (Supplementary Figure S12). We see similar but less striking trends in other signatures (Supplementary Figure S13 and Supplementary Table S6).

In summary, DNA hypomethylation determines local mutation rates in a manner independent of other features at regulatory elements and chromatin loop anchors, and independent of transcription levels or dynamics.

### Local methylation-aware mutation rate baselines can clarify signatures of selection

Methods to detect selection on somatic mutations in protein-coding genes and other functional elements rely on an accurate baseline of local mutation rates. This is applied to test whether there is an excess or dearth of mutations over that baseline, signaling positive or negative selection, respectively. Such baselines are typically established at the whole-gene level, and are based on a variety of mutation rate covariates such as DNA replication time, mRNA expression levels and histone marks.

**Figure 5.**
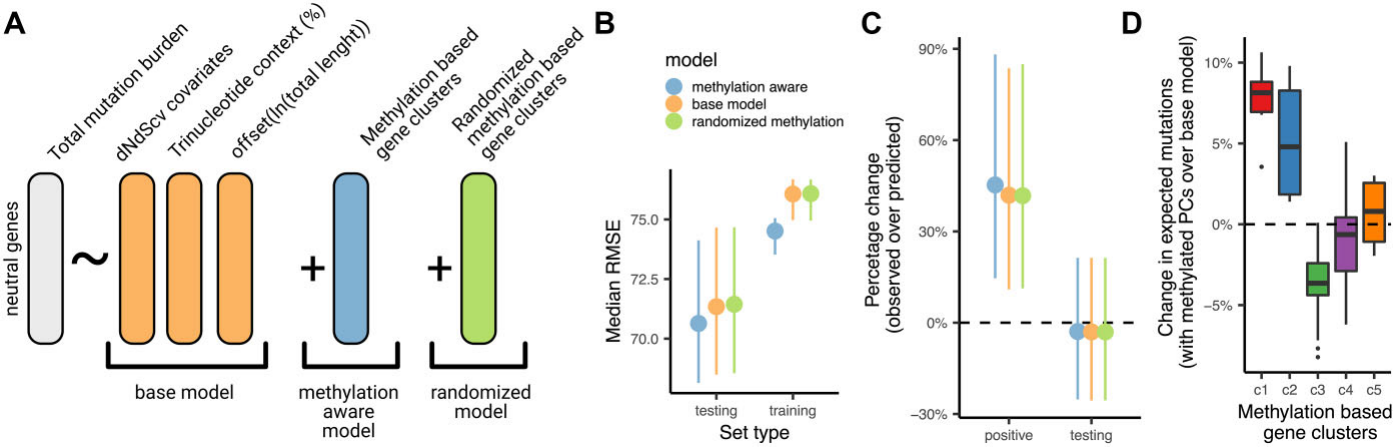
Methylation aware groups are relevant for selection estimation. (**A**) Diagram depicting a model to predict the mutation rate of genes according to epigenetic covariates (12), the context composition of the gene and the length as a offset. To this base model, the methylation-aware gene classes are added together with a randomized version of the gene clusters. (**B**) Root mean square error (RMSE) for the prediction of mutation rates by each model. (**C**) Percentage change of predicted mutations in the positive set (cancer genes with positive selection) and the testing set (genes that are used to evaluate the performance of each cross-validation round). (**D**) Changes in the predicted mutations burdens of genes for each gene cluster as defined in Figure 4.

We suggest that DNA methylation profiles of genes or other functional elements, capturing local variation in DNA methylation, may be considered in baselines that account for the within-gene heterogeneity in mutation rates. To provide a proof-of-concept analysis for utility of DNA methylation-aware baselines for mutation rates, we modeled the mutation burden of each gene from the 10 295 TCGA whole exome sequences (84). Because mutation rates are strongly associated with the epigenetic state and the replication time (85), as a base model, we predicted gene mutation rates from a set of epigenomic covariates from a state-of-the-art tool (dNdScv) (12). We then compared this with a model that includes the DNA methylation-profile gene groups labels defined above (c1–c5) as an additional covariate. As a negative control, we consider the same model but with the DNA methylation group labels shuffled (Figure 5A and Materials and Methods).

The root mean square error (RMSE) in number of exonic (including UTR) mutations per gene, using the average of 15 runs of five-fold cross validation, showed a better fit for the DNA methylation-aware model compared to both the base (covariate-only) model and the shuffled methylation-covariate model (Figure 5B). Significance of this improvement was tested by comparing the deviance using the observed *versus* DNA methylation-shuffled group labels (see Supplementary Methods). While in all iterations in the DNA methylation-aware model, the feature had a significant effect on improving the fit (*P*-value always <2e-16 in all 75 iterations), the shuffled-methylation label gene groups models were significant in only < 10% of the crossvalidation iterations (6 out of 75 at *P*-value < 0.01) (Supplementary Figure S14A).

Using the expected number of mutations predicted by this model, we can calculate the excess mutation burden for every gene, which is an effect size estimate of positive selection. This mutation burden excess was on average 45% in known broadly-acting known cancer driver genes (i.e. drivers in more than 2 tissues according to MutPanning (86), *n* = 131), which were excluded from the training and testing partition of the original data (Figure 5C). When considering non-cancer genes there was no significant excess in mutation rates between the different models, DNA methylation included as a covariate or not (Figure 5C).

Further indicating that gene DNA methylation patterns are an important factor to account for in identifying positive selection, the expected mutation burden differed significantly between different methylation-profile gene groups (Figure 5D and Supplementary Figure S14B). In particular, genes in the c3 group corrected their mutational burden prediction to lower values upon including DNA methylation covariate (with a −4% median adjustment, although this may differ in other datasets depending on the active trinucleotide mutational signatures). Example genes in this group are *EGFR*, *KRAS* or *RB1*, where the corrected expectation (to lower values) raised the observed excess mutation burden, suggesting a stronger positive selection than would be inferred without considering intragenic DNA methylation (Supplementary Figure S14B). Conversely, genes in group c1 and c2 (with a narrower and/or weaker hypomethylation region) showed a correction towards higher expected burden values when considering DNA methylation (with a median of +5% mutation burden for c1 and +3% for c2). *TSC1, FAM135B* and *APC* are examples from these groups, which show an overall reduction in their excess burden, suggesting the positive selection estimates would be revised downwards upon considering intragenic DNA methylation (Supplementary Figure S14B). The other methylation-aware gene groups, c4 and c5, showed only modest corrections (<1% change in excess mutation burden; median within group, although individual genes may have bigger deviations). Of note, genes classified by the Cancer Gene Census as either oncogenes (44.1%) or tumor suppressors (45.1%) were enriched in the active hypomethylated group c3 (Supplementary Figure S14C). Thus, our mutation risk model can capture relevant information for determining the excess mutation burden estimates for genes by accounting for their DNA methylation profile.

## Discussion

Somatic mutation rate variability was studied thoroughly at single nucleotide resolution, by tallying across oligonucleotide sequences (8–10), and it was additionally extensively studied across megabase-sized chromosomal domains (1,7). Prior work on the identification of mutation rate variability on the ‘mesoscale’ in between these two extremes—the coarse-scale and fine-scale heterogeneity—has considered some examples of genomic features that interact with damage formation or repair (14,23,80). Here, motivated by the known intra-gene gradients in epigenetic marks that tend to occur in active genes and that are relevant to DNA repair (30,31,35), we systematically tested for the local mutation rate gradients along gene bodies across 40 mutational signatures, separately considering tissues and gene expression levels. This highlighted the local DNA hypomethylation at promoters and further extending typically ∼2 kb downstream into gene bodies (and more generally at other hypomethylated loci along the genome such as chromatin loop anchors) as a major influence on mutation rate heterogeneity at the kilobase-scale.

In our unbiased analysis of mutation rates, which adjusted for trinucleotide composition of various gene segments, the 5′ gene part was the focus of the top trends in variation between mutational signatures and gene expression levels (Figure 1D). We observed the strong depletion of the common SBS1 clock-like signature, likely to be caused by the deamination of a methylated cytosine (5mC) (8,39,40) and thus should be reduced by the absence of 5mC. In addition to this pattern relying on a well-established mechanism, we also noted the DNA repair deficiency-associated SBS10b and SBS15 (Figure 1A, C), recently linked with DNA methylation (39,40) through the mispairing with adenine instead of guanine during replication (38). Thus, based on these known and anticipated mutagenic mechanisms of 5mC, we infer that the gradients in DNA methylation across genes are the cause of locally variable mutagenesis via generating mutational coldspots at 5′ ends of genes and elsewhere. We also note that the hydroxymethylated cytosine, which is also enriched around the promoters of post-mitotic tissues (87–89) was previously shown to decrease CpG > T (SBS1) mutation accumulation (90,91). Of note, all our analyses concern autosomal genes, and exclude sex chromosomes to avoid confounding with mutagenesis associated with X-specific mechanisms of inactivation (92).

An important exception regarding the genic mutational coldspots reported herein is that they may change to hotspots under conditions of APOBEC mutagenesis (SBS2 and SBS13) commonly observed across many somatic tissues, or the more specialized SHM associated signatures (SBS9, SBS84) observed in B-lymphocytes. Similar to how mutations from the AID-initiated SHM process are well-known to be enriched at or near promoters (54), we also show this is the case for 5′ gene end enrichment of the mutations by its APOBEC paralog(s), although the underlying mechanism may be different. The AID may sense 5′ stalled RNA polymerase (93), while we speculate that APOBEC deamination may be more mutagenic at 5′ gene ends due to DNA methylation itself protecting against APOBEC in the remainder of gene body. Links between higher APOBEC activity and DNA demethylation have been reported (94), however other studies did not show notable associations (95). We note it is also possible that the causality flows the other way: rather than DNA methylation blocking APOBEC, APOBEC could in principle remove DNA methylation in a non-mutagenic manner. AID, a homolog of APOBEC enzymes, was claimed to participate in active DNA demethylation via triggering DNA repair (96,97). By analogy to this, speculatively, an increased activity of APOBEC at 5′ gene ends, directed thereto by an unknown mechanism, might contribute to reduced DNA methylation; additionally the same mechanism of directing APOBEC would cause mutations in those regions. An increased burden of APOBEC mutations associated with actively transcribed genes was previously reported (98), and here we address the distribution of the APOBEC mutations across the segments within these genes, which suggests importance of a DNA methylation-based mechanism for local APOBEC hotspots.

We also show that a gene classification by the extent of their hypomethylated region at the 5′ end (Figure 2E) is reflected in the extent and the intensity of the mutation coldspot in the gene 5′ section (Figure 2H). This variable DNA hypomethylation and thus mutation rates within genes associates with occurrence of intragenic enhancer regions, chromatin loop anchors, or in some cases Polycomb marks, and possibly additional factors associated with local hypomethylation yet to be identified. We suggest that gene body local DNA methylation variability should be included in models for testing selection on somatic mutations at the gene level. Finding the best implementation of this principle remains a direction for future work, where for instance selection on different gene segments might be considered individually, sample size permitting. Further, methods that incorporate DNA methylation into mutation rate baselines may be developed for more accurately estimating selection on non-coding regulatory regions.

Prompted by the mutational coldspots at 5′ gene ends, we extended our analysis to genome-wide hypomethylated regions (both UMRs, and the partially methylated LMRs (43,44)). These were, as expected, associated with promoters and enhancers, however interestingly also with chromatin loop anchors, suggesting hypomethylation is common at these loci (Figure 4B, C). There was also a residual fraction of hypomethylated regions distributed across the genome, not overlapping known elements, however still constituting mutational coldspots, supporting proposed mechanisms underlying mutation reduction (39,75).

Overall, we highlight the role of local DNA hypomethylation in shaping mutation rate heterogeneity in the human genome, and stress the need to further characterize this local, mesoscale variation in mutation risk and the underlying mechanisms.

## Supplementary Material

gkae252_Supplemental_Files

## Data Availability

No new data were generated for this study. The datasets utilized for somatic mutations and DNA methylation (WGBS) are provided in Supplementary Tables S1 and S2, respectively. The source code for the data processing and analysis has been archived in Zenodo (DOI: 10.5281/zenodo.10516368).
