# Supplementary material for: Mutation rate heterogeneity at the sub-gene scale due to local DNA hypomethylation
Source: Nucleic Acids Res. 2024 Apr 8;52(8):4393–408. doi: 10.1093/nar/gkae252 (PMC11077091; doi:10.1093/nar/gkae252)
Supplement: gkae252_Supplemental_Files [file gkae252_supplemental_files.zip › SupplementaryFigures_upd.pdf]

Supplementary Material for *Mutation rate heterogeneity at the sub-gene scale due to local DNA hypomethylation* (2024)

Mas-Ponte, David **Supek, Fran**

Contents

Supplementary Figures 2
Supplementary Figure S1 2
Supplementary Figure S2 3
Supplementary Figure S3 4
Supplementary Figure S4 6
Supplementary Figure S5 7
Supplementary Figure S6 9
Supplementary Figure S7 11
Supplementary Figure S8 12
Supplementary Figure S9 13
Supplementary Figure S10 15
Supplementary Figure S11 16
Supplementary Figure S12 17
Supplementary Figure S13 19
Supplementary Figure S14 20
Supplementary Figure S15 21
Supplementary Figure S16 22
Supplementary Tables 23

Supplementary Figures

Supplementary Figure S1

Supplementary Figure S1 is available as a separate PDF file

Gene gradient plots for individual regressions. Coefficients (in points) and 95% CI (as vertical lines) of the regression values from a gene gradient analysis. Each point represents a 250 bp long bin distributed along the gene body in a region around the 5' end, the central location and the 3' end (same as in Fig. 1). Values represent the coefficients in a pan-cancer setting, thus, all mutations assigned to a given signature across cancer types are considered. Furthermore, genes are split across average GTEX expression level in equal sized terciles. Eq1 represents lower expression levels and Eq3 higher. (Relative to Fig.1A).

Supplementary Figure S2

Supplementary Figure S2 is available as a separate PDF file

Coordinates of individual points in the gene-gradient PCA. Coordinate values of every row included in the PCA of the gene gradients. Mutations were stratified in tissue, expression level, and signature. Thus, every square contains information about a single instance in the PCA. Empty squares result from no mutations assigned in that tissue of origin and signature, or alternatively, due to a failed regression because of low counts. (Relative to Fig.1C and Extended Data Fig. 1E).

Supplementary Figure S3

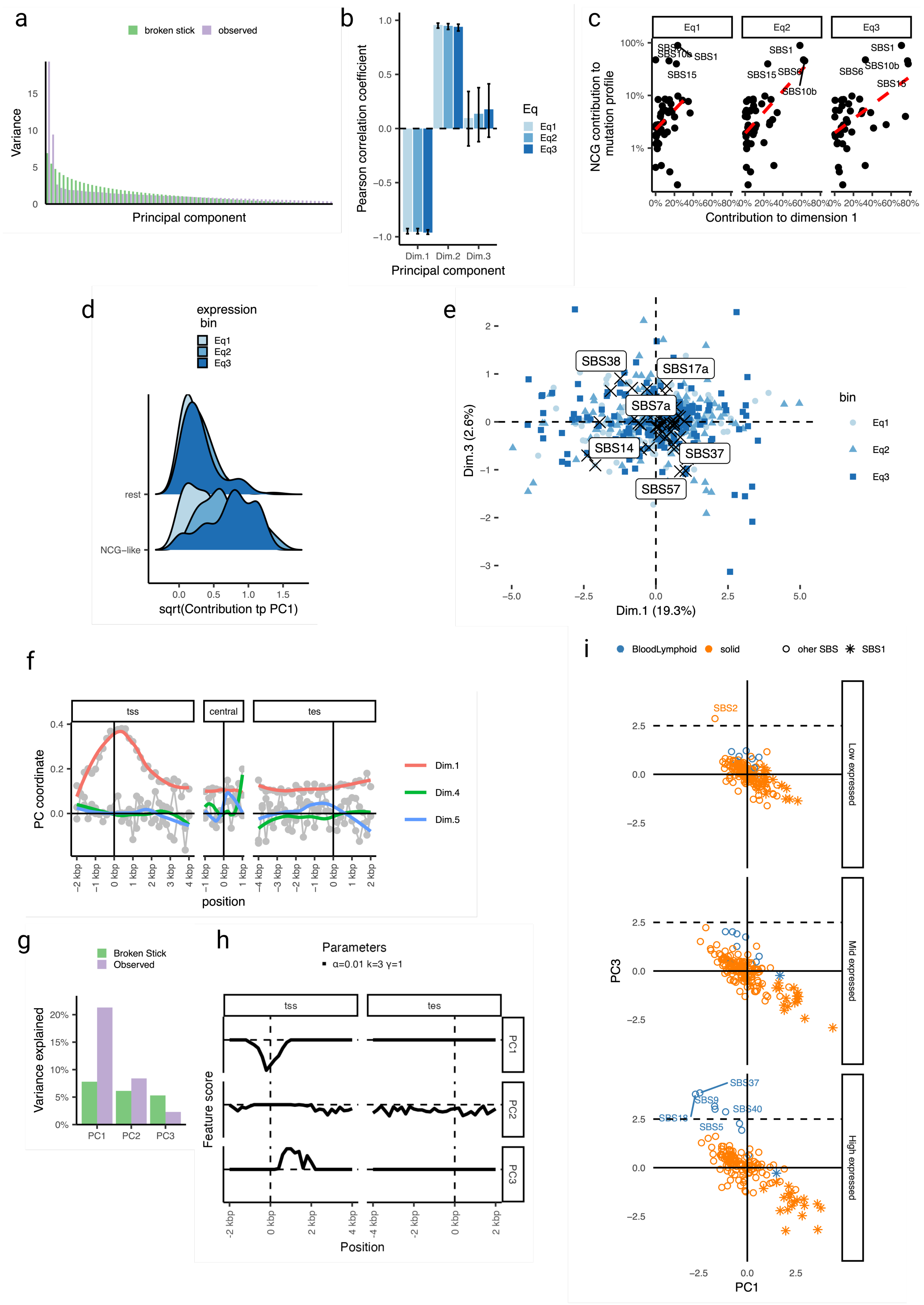

Systematic quantification of mutation gradients along gene bodies. (a) Scree plot from the gene gradient mutation rate PCA depicted in Fig.1. (b) Correlation of the percentage of CG trinucleotides in each signature compared to the total contribution to the first principal component. (c) Same as in (b) but instances are stratified by gene expression and signatures are classified in CG-like or rest according to the CG percentage in their profiles. (d) Signature contribution to PC1 as in (c) but grouping signatures according to high NCG (NCG-like) or low (rest) values. (e) Coordinates of mutation signature profiles in different sets, same as in Fig. 1C but with Dim.3. (f) Weight profiles of PC4 and PC5 (with PC1 as scale). (g) Scree plot for the sparse PCA performed in the mutational gradient matrix with 3 components. (h) Profiles of the 3 components extracted with a sparse PCA. (i) Coordinates of different signatures colored by tissue, blue for Blood Lymphoid samples and orange for others. Shape as an asterisk for SBS1 and an empty circle for other signatures.

Supplementary Figure S4

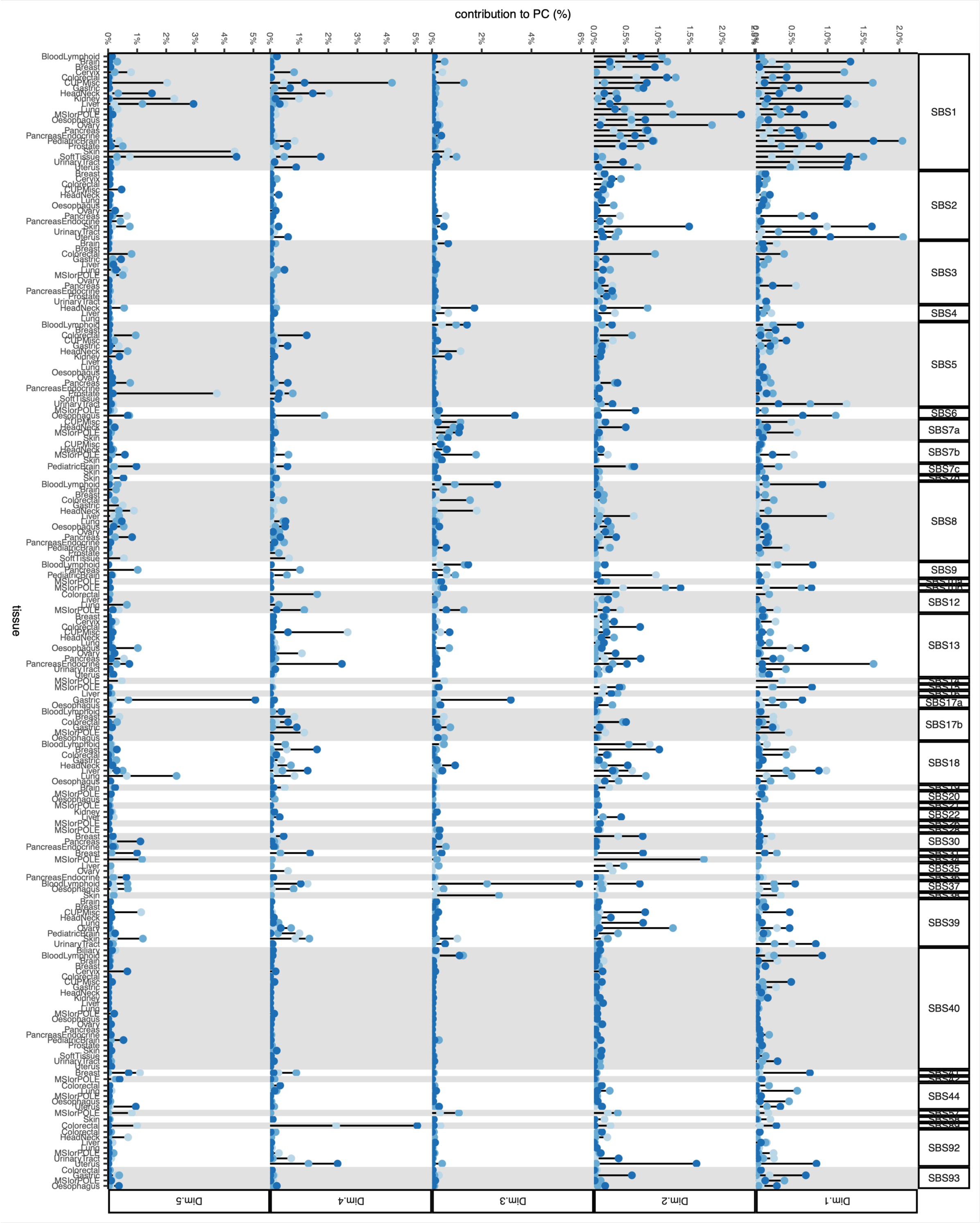

Contributions of each instance into the gradient PCA analysis. The contribution percentage of every instance in the PCA to the five top components. Contributions are represented as percentage so negative and positive coordinates can have both higher contributions although opposite trends. The total sum adds up to 100% for each component (Relative to Fig.1C and Extended Data Fig. 1E)

Supplementary Figure S5

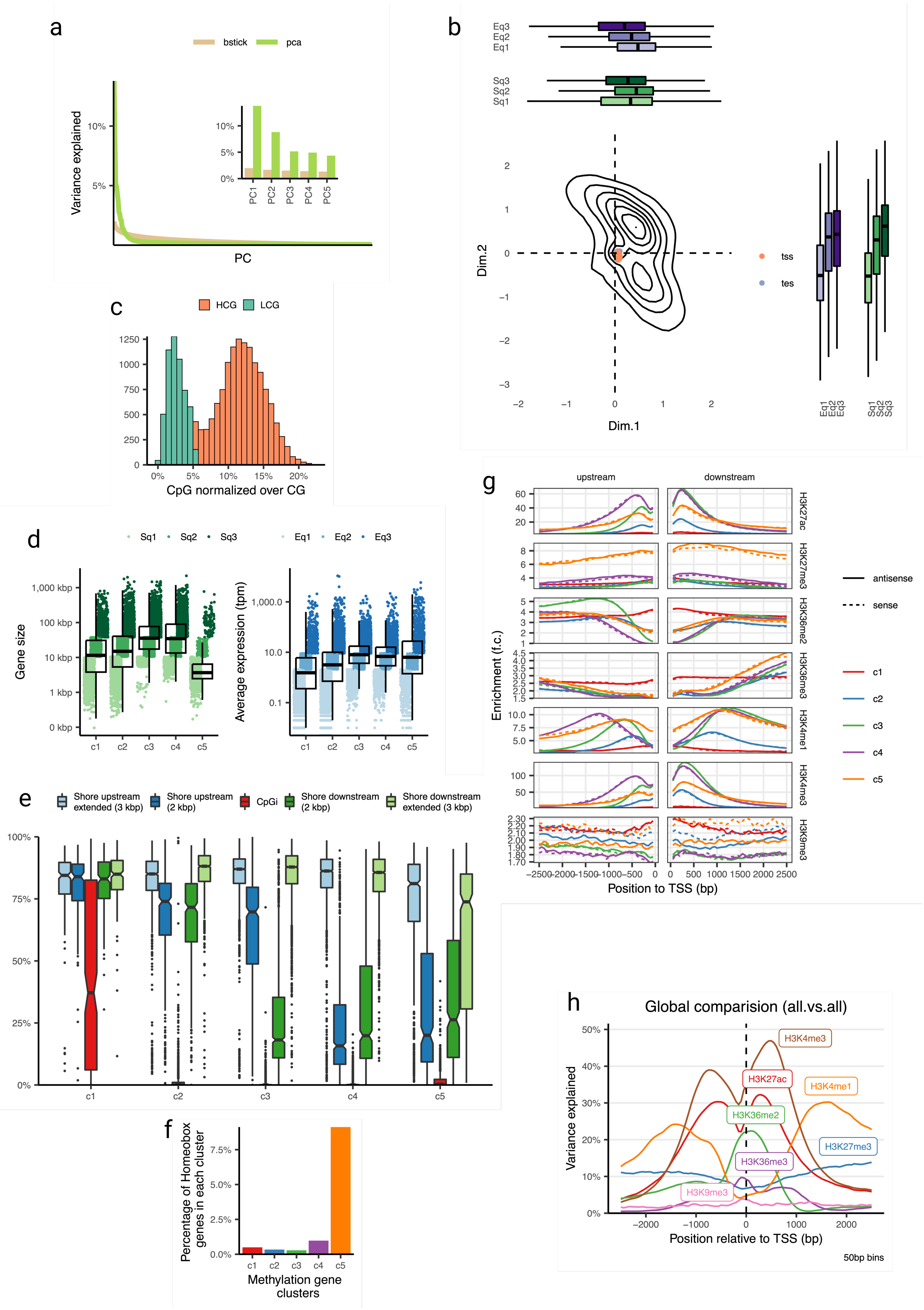

Clustering of genes according to their methylation profile. (a) Scree plot of the methylation profile PCA used to cluster genes. (b) PCA coordinates of each gene (represented as a 2D density plot) with the expression and size distribution for each principal component represented in boxplots. (c) Definition of the HCG genes according to their normalized CG values. A mixture modelling is used to define a numeric cut-off. (d) Expression and size terciles of each gene methylation class. (e) Enrichment of histone mark signal plots (fold change extracted from ENCODE ChIP-seq experiments) both for sense and antisense genes in each methylation aware group. (f) Proportion of Homeobox genes, as defined in ref89, for each methylation aware group. (g) Methylation levels across different methylation aware gene groups including their CpG island shores (2kb from the upstream and downstream end of the CpG island) and a extended region (h) Histone weights per position (relative to e) in a linear model to classify methylation aware gene groups.

Supplementary Figure S6

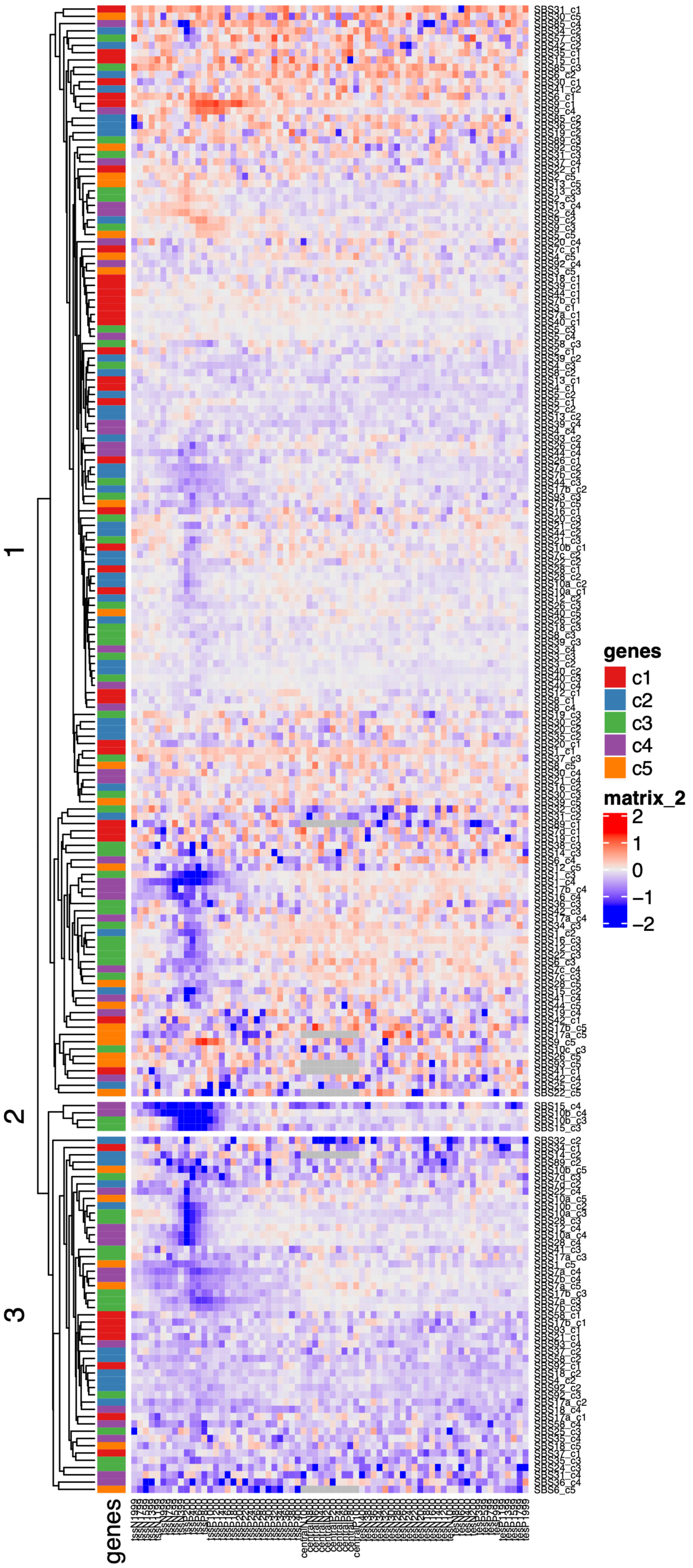

Gene gradient mutation rate coefficients for methylation aware gene groups. Mutation rate gene gradients (as in Fig. 1) were calculated for each mutational signature in groups of genes divided according to their methylation profiles (from Fig. 2). The heatmap shows the coefficient values (in ln scale) of the 5' end, central and 3' end gene sections. Rows are clustered and annotated with the methylation group of genes where the regression was performed.

Supplementary Figure S7

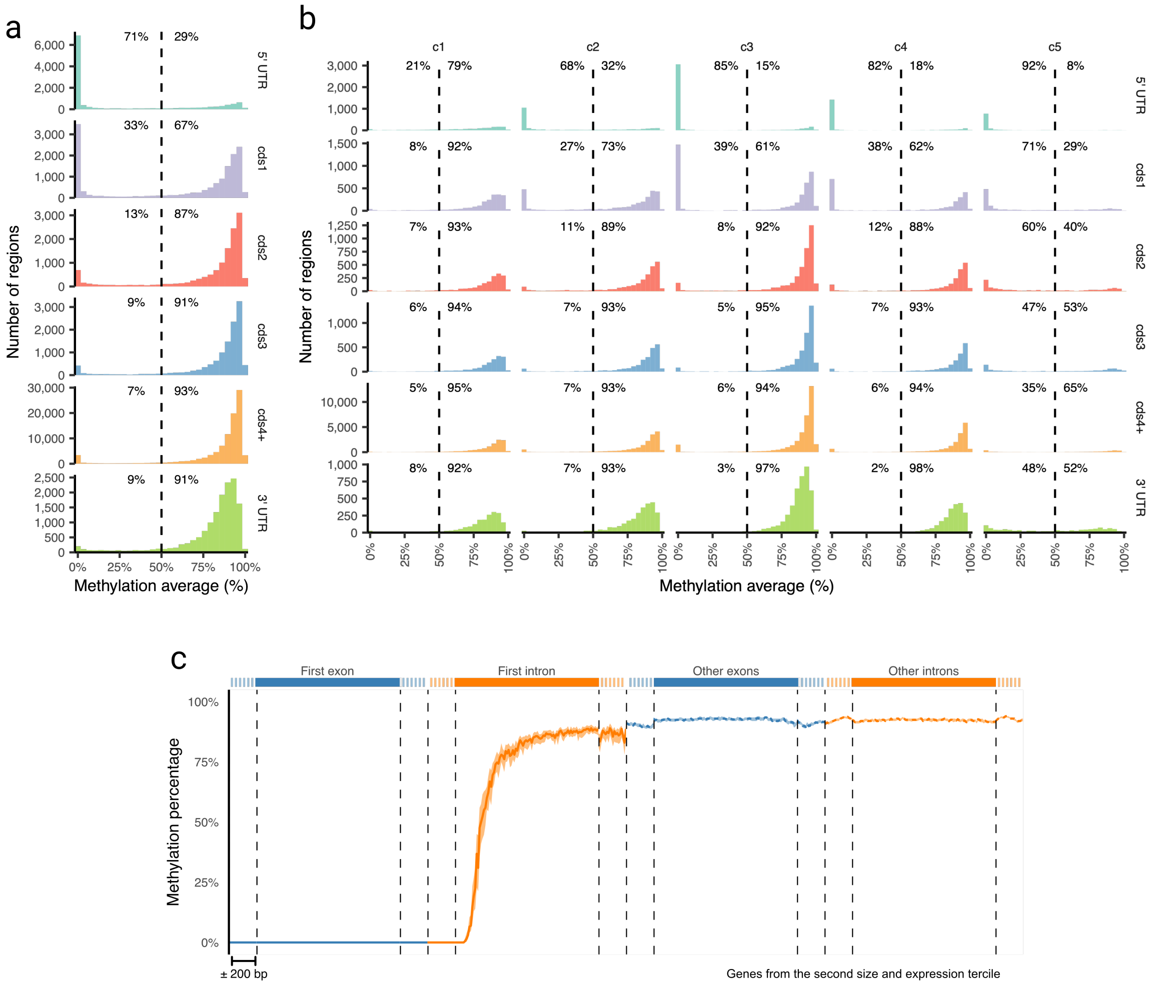

Histogram of average methylation across gene groups. (a), further subdivided in distinct gene sections (b), the 3' and 5' UTR exons and the first, second and third coding exons from the 5' end of the gene body. (c) Methylation average values of genes encompassed in the mid expression and mid size category subdivided by introns and exons. Dashed sections represent extended 200bp from the section end.

Supplementary Figure S8

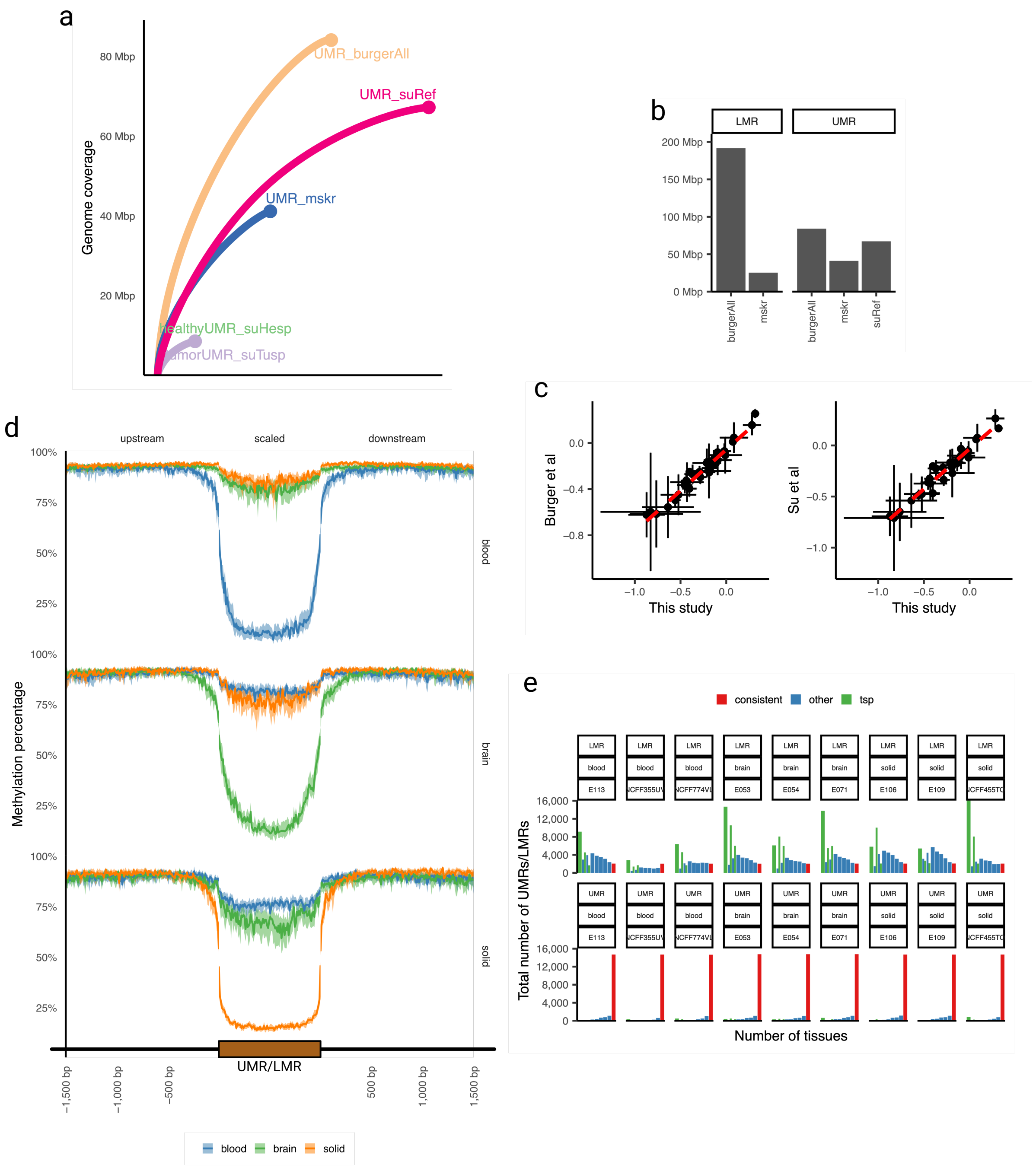

UMR and LMR. Genome-wide unmethylated regions and quantification of mutation rate. (a-b) Genomic coverage of the selected UMRs. (c) Correlation of the mutation rate estimations in different UMR sets. (d) Methylation levels in tissue specific UMRs. (e) Number of tissue specific (green), consistent (red) and other (blue) UMRs and LMRs from the comparison of 3 brain, blood and digestive datasets. In the x-axis, the number of sets that contain a given type of UMRs is represented, ranging from 1 to 9.

Supplementary Figure S9

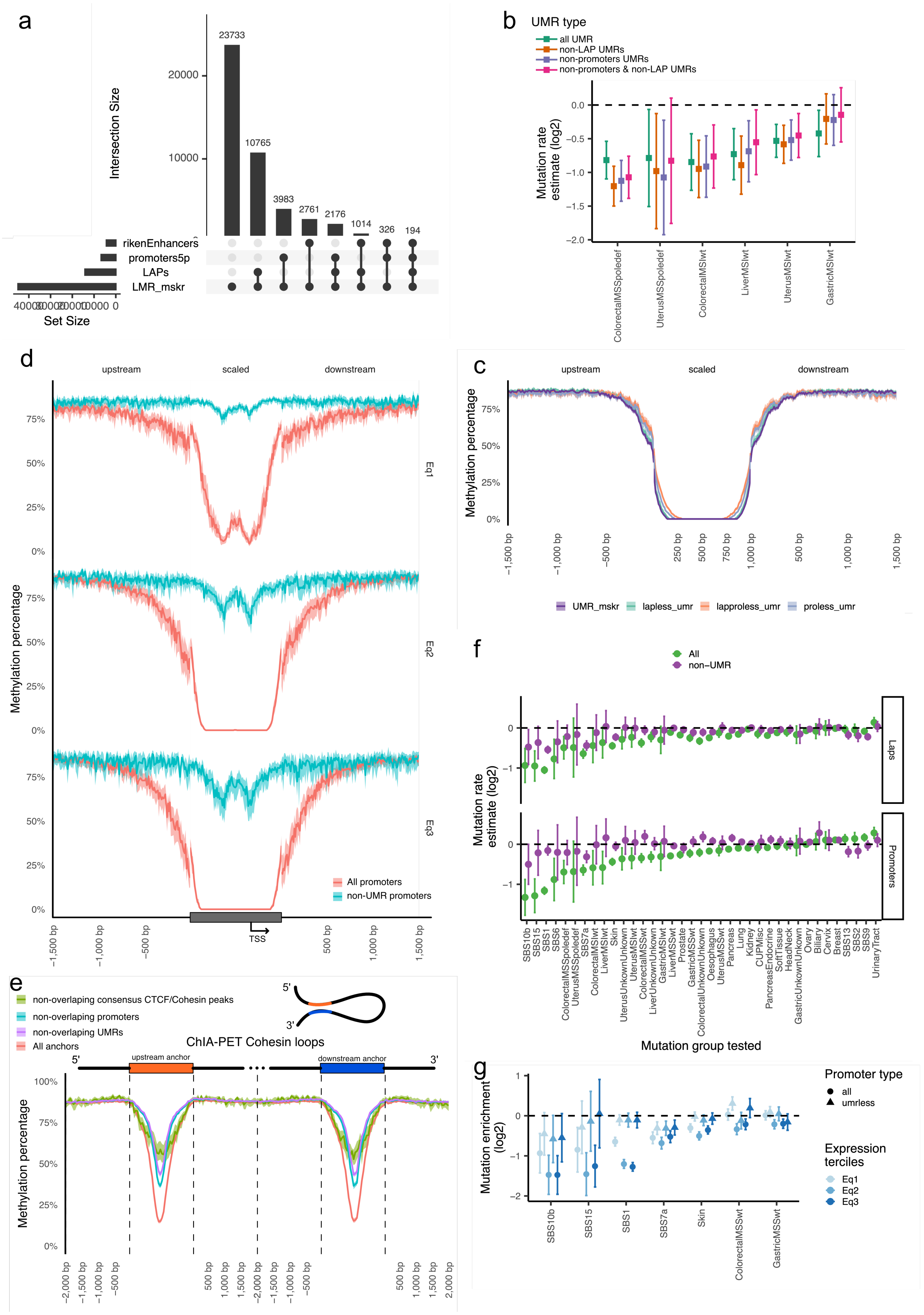

Functional elements associated with UMRs and LMRs. (a) Upset plot representing the overlap with functional elements and LMRs. Only functional elements that overlap with any LMR are included in the figure. (b) Mutation estimates in functional element free UMRs for DNA repair deficient tissues. (c) Methylation level of all UMRs and others that do not show overlap with promoters (proless) or loop anchors (lapless) or both (lapproless). (d, e) Methylation level of all promoters and loop anchors, overlapping and not overlapping UMRs and other functional elements for LAPs. Methylation level is represented as the median average value of all cases. Shadow represents the 95% binomial confidence interval of the median. (f) Mutation rate estimates in promoters that significantly overlap with a UMR (> 200bp) and all promoters. (g) Same as in f but for selected tissues and stratifying the promoters according to the expression bins.

Supplementary Figure S10

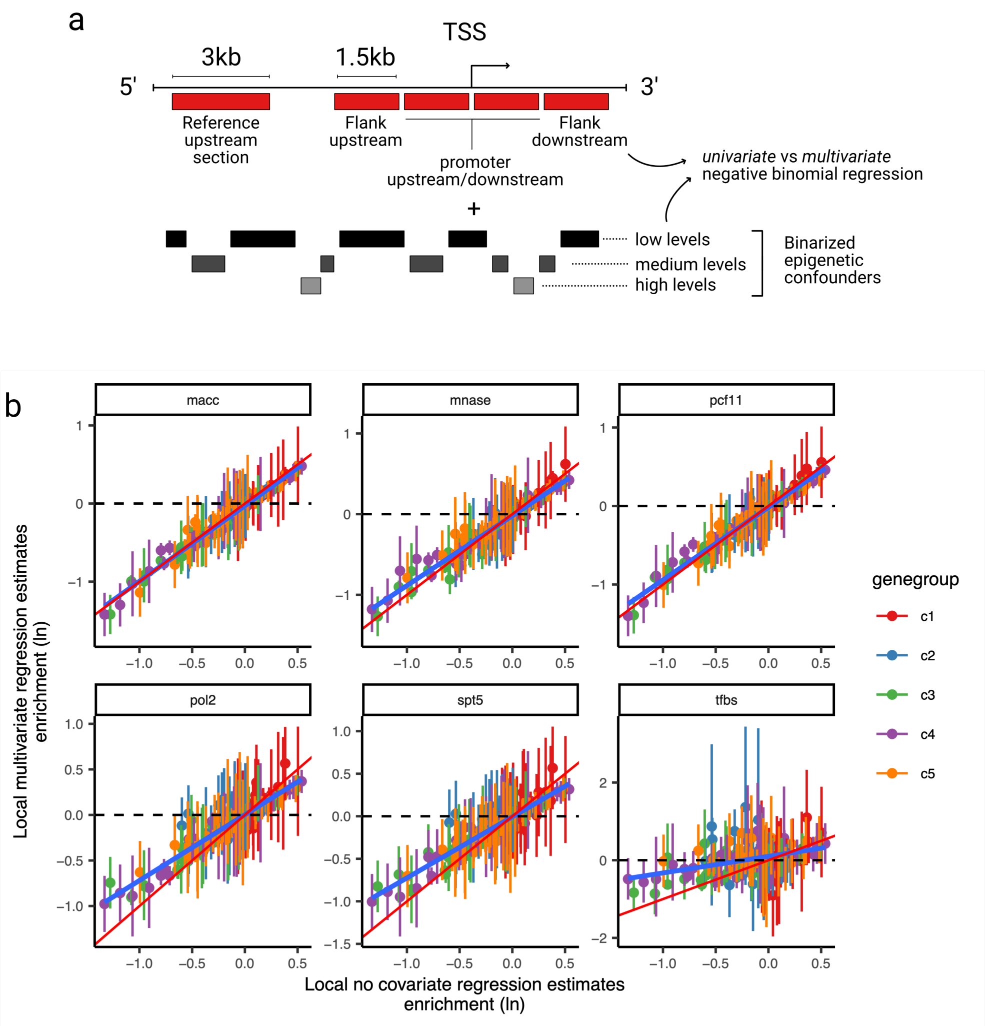

Role of additional epigenetic factors in the 5' gene end mutation rate depletion. (a) Diagram of the analysis. In brief, mutations are assessed per gene group at four 1,5 Kb wide bins compared to a 3kb reference bin located 12 kb bins upstream of the gene. The four regions are termed, flank upstream, promoter upstream, promoter downstream and flank downstream. (b) Correlation of the local 5' gene end gradient coefficients (mutational enrichments) for the selected signatures (SBS1, SBS10a, SBS10b, SBS7a, SBS7b, SBS2, SBS13, SBS15 and SBS6) in the univariate regression (x-axis) and the multiple variate regression including one of the additional epigenetic factors (y axis). Error bars represent the 95% confidence interval of the regression. Red line represents the 1:1 ratio and blue line the empirical correlation. The selected factors are: MACC, that represents nucleosome accessibility, MNase, subdivided in nucleosome free, nucleosome bound, nucleosome unbound and rest of the genome as reference group; pcf11 signal bins that represent early transcription termination; spt5 and pol2 signal bins that represent enrichment in polymerase levels due to transcriptional statement and transcription binding sites (TFBS) that correspond to predicted transcription binding sites along the genome and reference sequences located 1,000 bp downstream from the original sequence. We excluded the estimate for the upstream flank in the c2 gene group for signature SBS15 in the TFBS panel due to a large CI (1.46 to −4.75) that impeded the correct visualization of the trend.

Supplementary Figure S11

Supplementary Figure S11 is available as a separate PDF file

Role of additional epigenetic factors for all selected mutational signatures. Mutation enrichments (expressed in natural log coefficients of the negative binomial regression), for mutational signature SBS1and all other mutational signatures tested (SBS10a, SBS10b, SBS7a, SBS7b, SBS2, SBS13, SBS15 and SBS6) at different local bins around the transcription start site. Each group of genes is treated independently and here represented in different colors. In the left panel, we show the enrichments of local TSS bins for both the single variate regression (solid) and the multivariate regression (dashed) including the epigenetic cofactors: SPT5, POL2, PCF11, Transcription Factor Binding Sites (TFBS), MNase Accessibility (MACC) and direct MNase readouts (MNase). In the right panel, we show the mutational enrichment for the cofactors bins, including a univariate regression (square, gray point) and the multivariate for each group of genes (circle). Data values for the figure are available in Supplementary Table 5.

Supplementary Figure S12

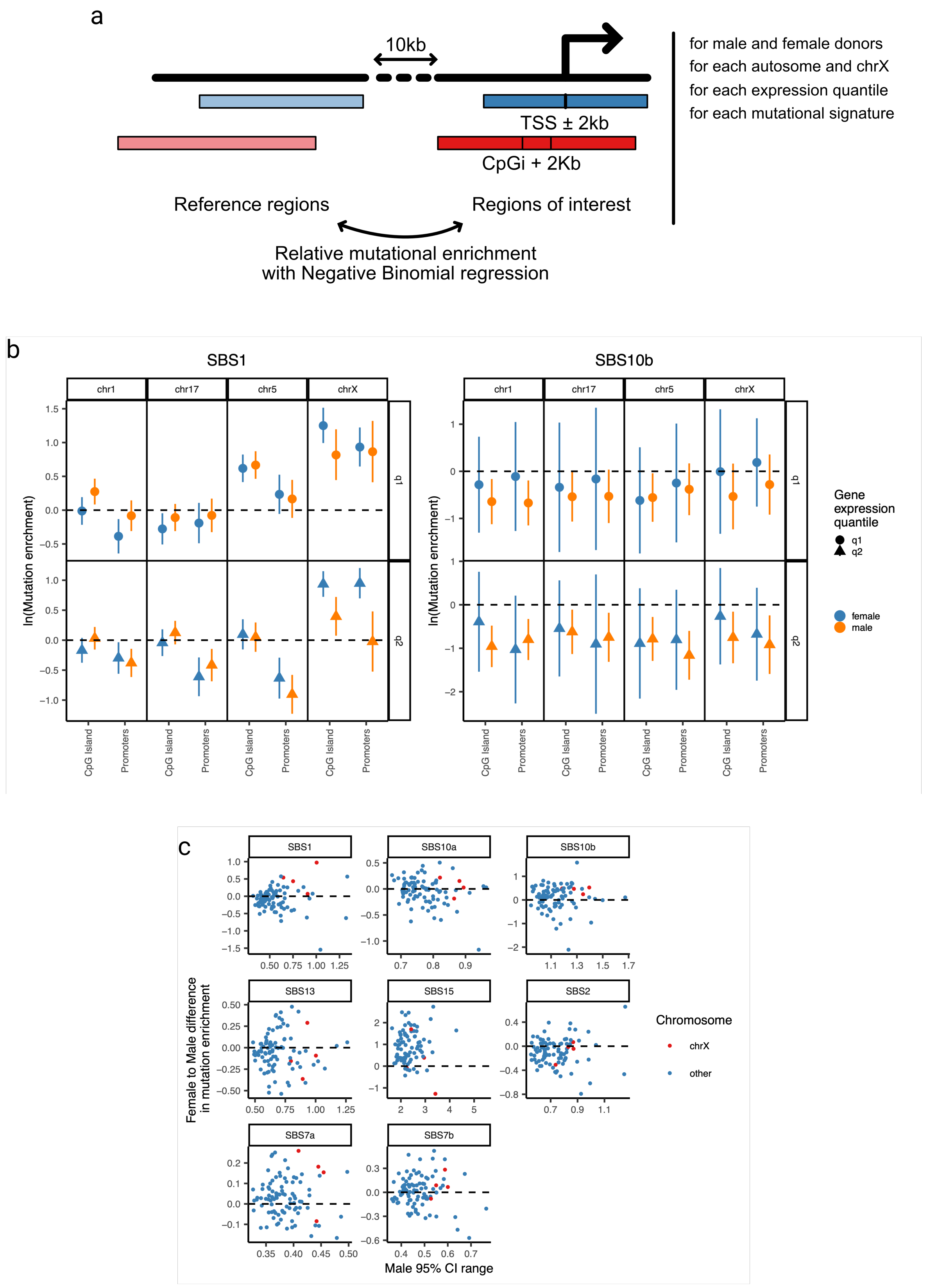

Sex-specific mutation enrichment in different chromosomes. (a) Schematic of the analysis performed to measure relative mutation rate enrichment on individual autosomes and chromosome X. See Supplementary Methods for extensive details. (b) Analysis of the relative mutation enrichment for SBS1 (left) and SBS10b (right) in 5' gene ends and CpG islands in a group of selected autosomes (chr1, chr5 and chr17) and chrX. Both 5' gene ends and CpG islands were divided into highly expressed (q2) and lowly expressed (q1) genes. The tested mutations were also divided according to the biological sex of the donor. Thus, each point and whisker represents a single negative binomial regression. Y axis is the coefficient of the negative binomial regression in natural logarithm units. (c) Female to male difference in the calculated mutation enrichment coefficient, higher values indicate female samples have an increased mutation enrichment compared to males (y-axis) and the 95% confidence interval in the regression coefficient from the male samples (x-axis). Regressions that correspond to the chrX are highlighted in red. All other tested signatures are available in Supplementary Table 6.

Supplementary Figure S13

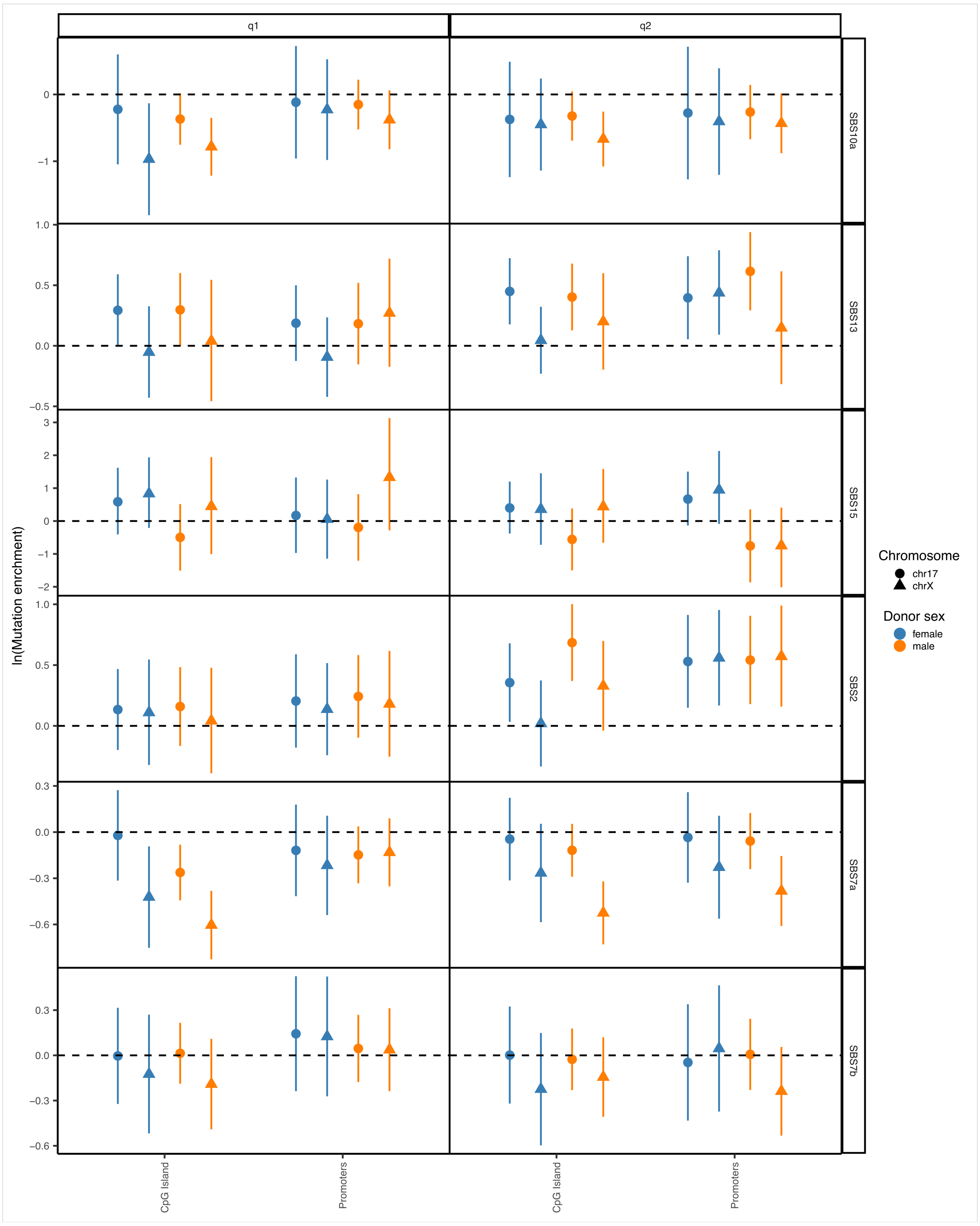

Additional sex-specific mutational enrichments. Same as Supplementary Fig. 12b but for the rest of selected signatures in chrX and chr17. All other autosomes are available in Supplementary Table 6.

Supplementary Figure S14

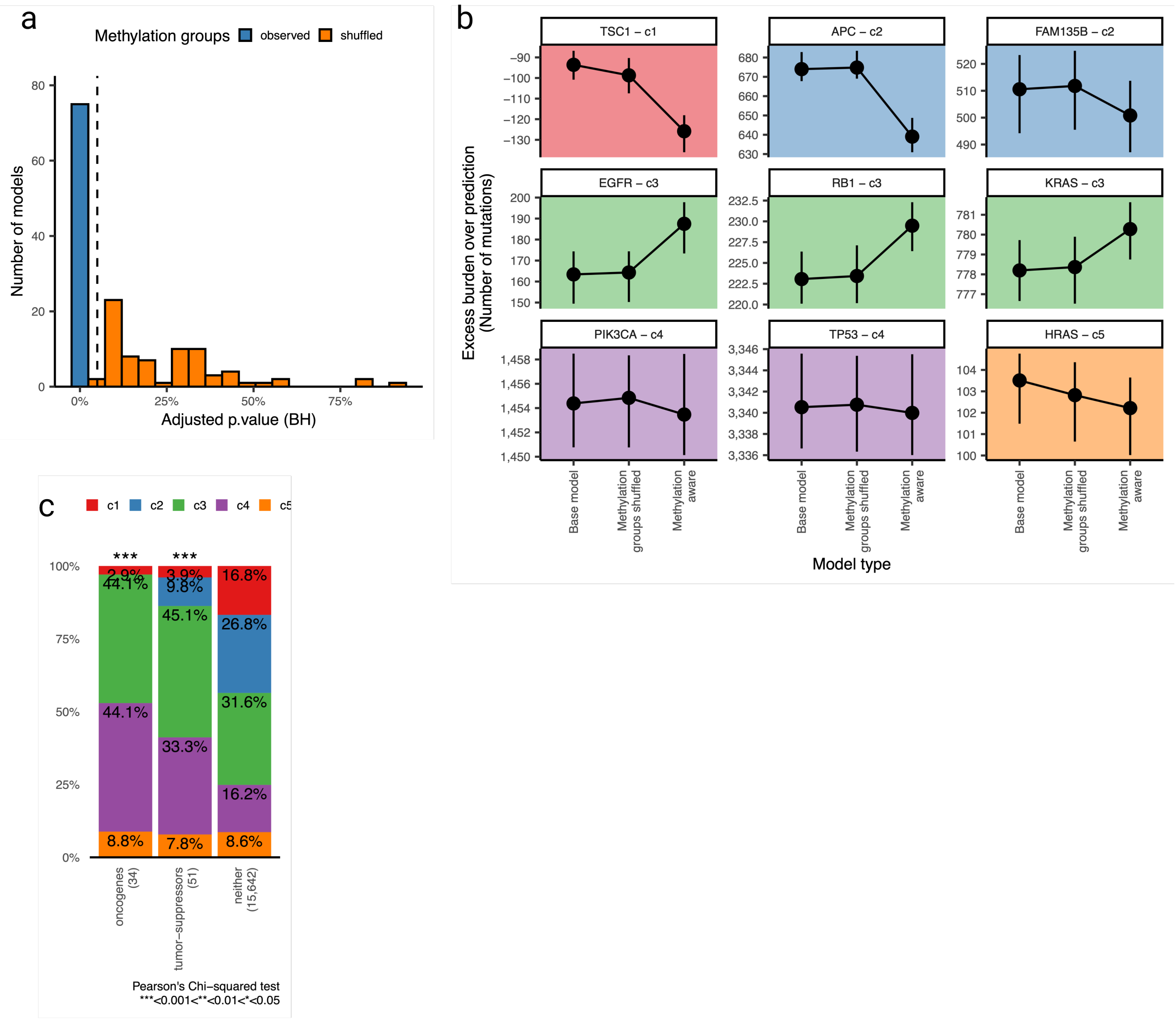

Gene mutation burden prediction with methylation aware gene classes. (a) Significance measured by log-ratio test (see Methods) of a model including the observed or a shuffled version of the methylation aware gene groups. Vertical dashed line indicates significance threshold (0.01). 6 out of 75 models with shuffled groups showed significance. (b) Selected examples of burden excess, mutations observed versus mutations expected, for some cancer driver genes. Colors of the box represent their gene group. Note axes are different in each box. (c) Proportion of methylation aware gene groups in cancer drivers (oncogenes and tumor-suppressors, left and center respectively) and all other genes (right).

Supplementary Figure S15

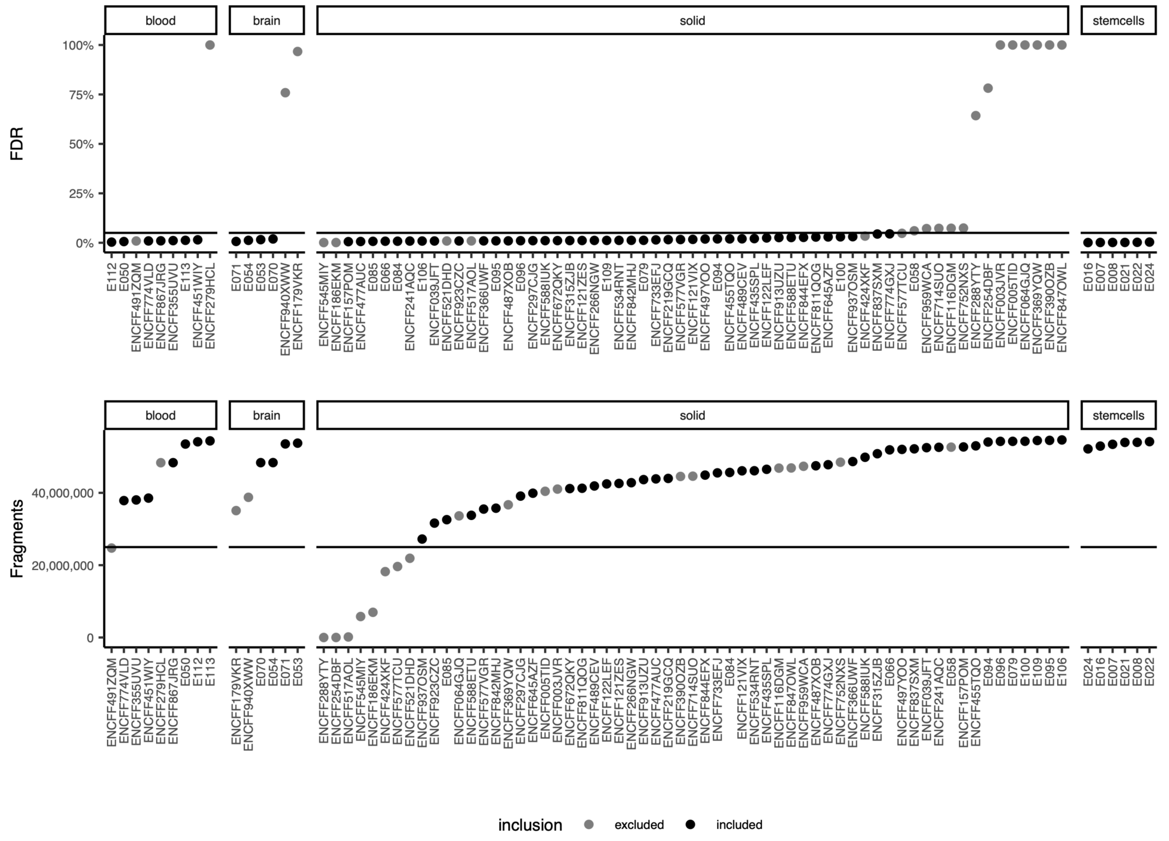

Excluded WGBS samples from MethylSeeker output. MethylSeeker FDR for UMR/LMR calling at predefined cutoffs (solid lines, see Methods). Each row contains a selected experiment. (top) Global false discovery rate for a single sample. (bottom) Total number of CpG fragments obtained from every sample. (Relative to Methods and Supplementary Table 2).

Supplementary Figure S16

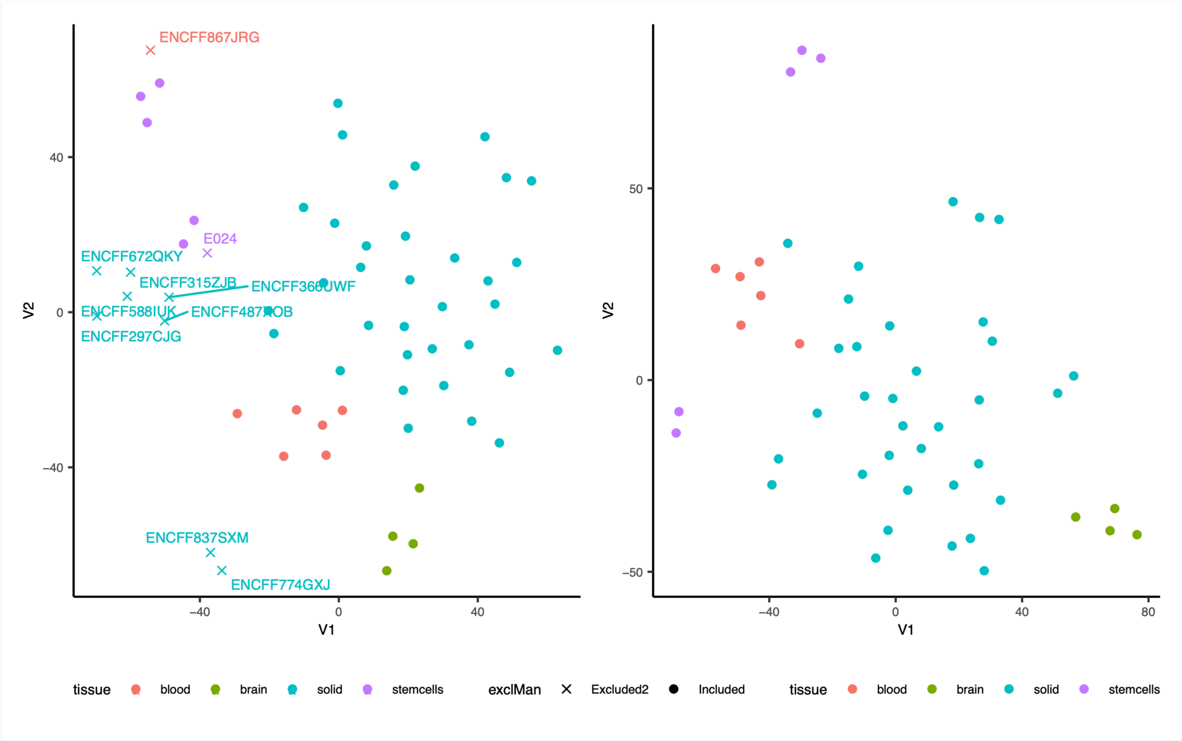

Manually excluded WGBS samples. tSNE plot from UMR/LMR detected loci in WGBS samples (see Online Methods). All samples showed had passed the previous thresholds in MethylSeeker. After manual inspection, samples that did not cluster with the intended tissue were manually excluded (marked with a cross) and removed from the analysis.

Supplementary Tables

Supplementary Tables S1 – S6 are available as external files.

Supplementary Table S1. Somatic mutation data sources.

Supplementary Table S2. WGBS data sources.

Supplementary Table S3. Methylation aware gene groups.

Supplementary Table S4. UMR and LMR literature extracted datasets.

Supplementary Table S5. Relative to Supplementary Figure S10 and 11. Relative mutational enrichments in the analysis of additional epigenetic factors for all tested signatures (SBS1, SBS10a, SBS10b, SBS7a, SBS7b, SBS2, SBS13, SBS15 and SBS6).

Supplementary Table S6. Relative to Supplementary Figure S12 and 13. Relative mutational enrichments in the analysis of 5' gene ends and CpG islands in individual autosomes and chrX for all tested signatures.
