# Supplementary material for: Mutation rate heterogeneity at the sub-gene scale due to local DNA hypomethylation
Source: Nucleic Acids Res. 2024 Apr 8;52(8):4393–408. doi: 10.1093/nar/gkae252 (PMC11077091; doi:10.1093/nar/gkae252)
Supplement: gkae252_Supplemental_Files [file gkae252_supplemental_files.zip › Supplementary_Figure_S1.pdf]

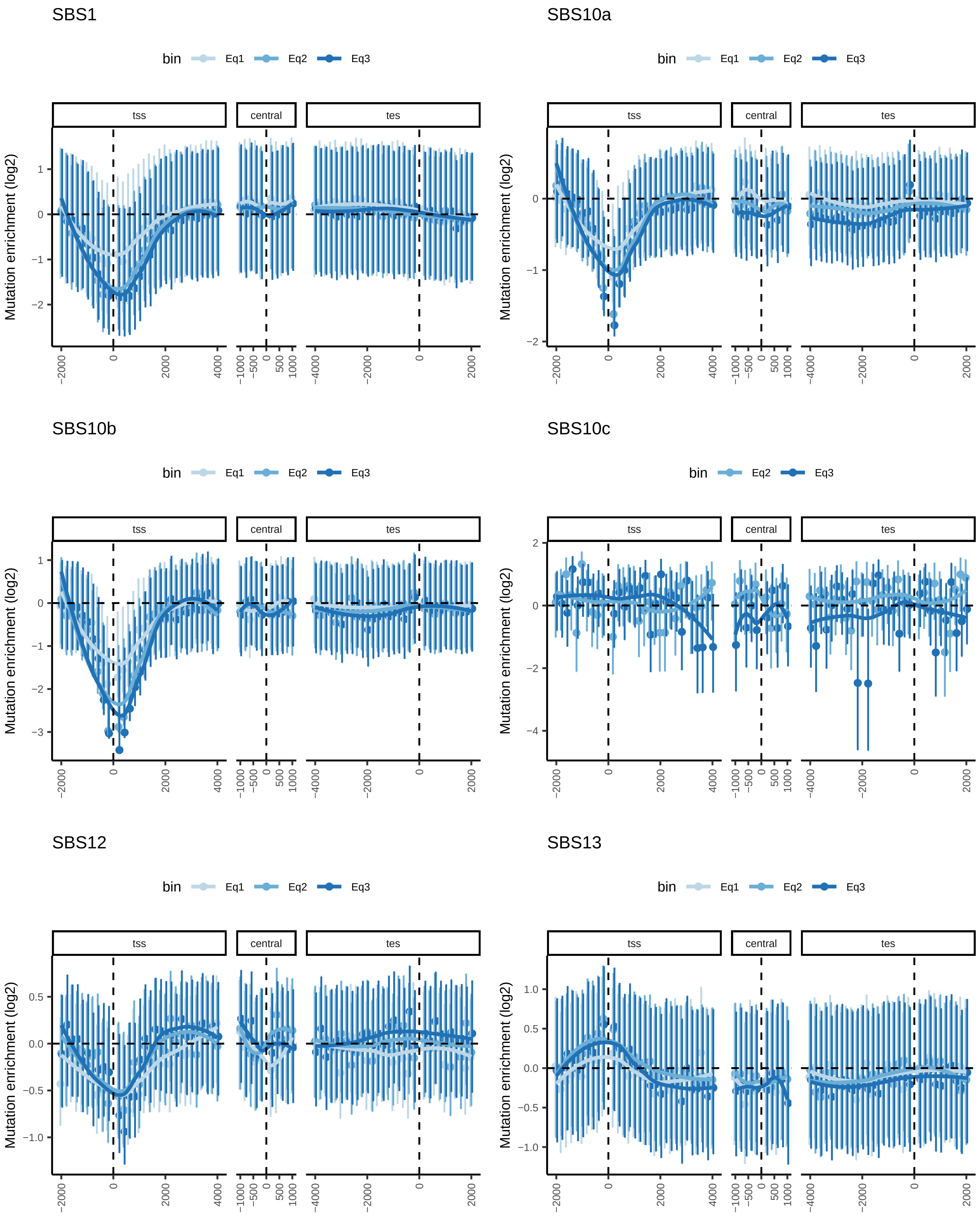

SBS1
SBS10a
SBS10b
SBS10c
SBS12
SBS13
bin
Eq1
Eq2
Eq3
tss
central
tes
Mutation enrichment (log2)
-2000
0
2000
4000
-1000
-500
500
1000
-4000

SBS14

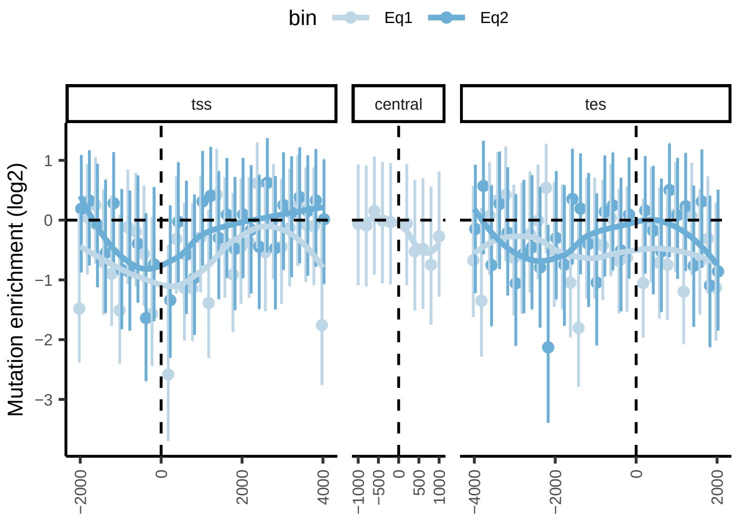

SBS15

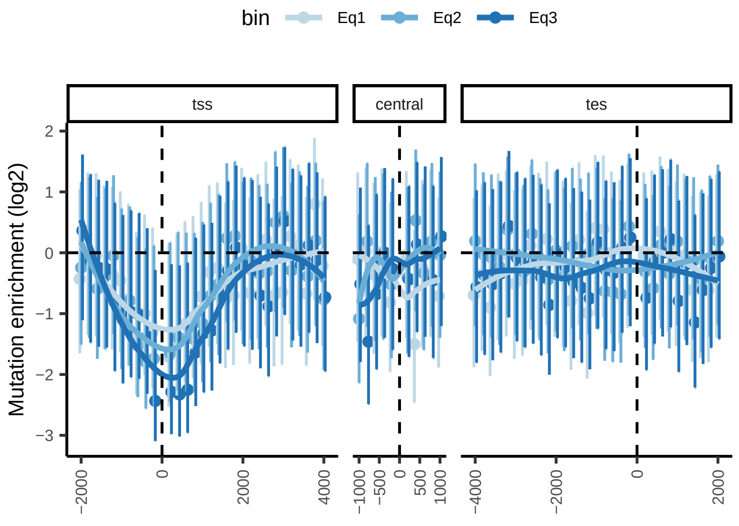

SBS16

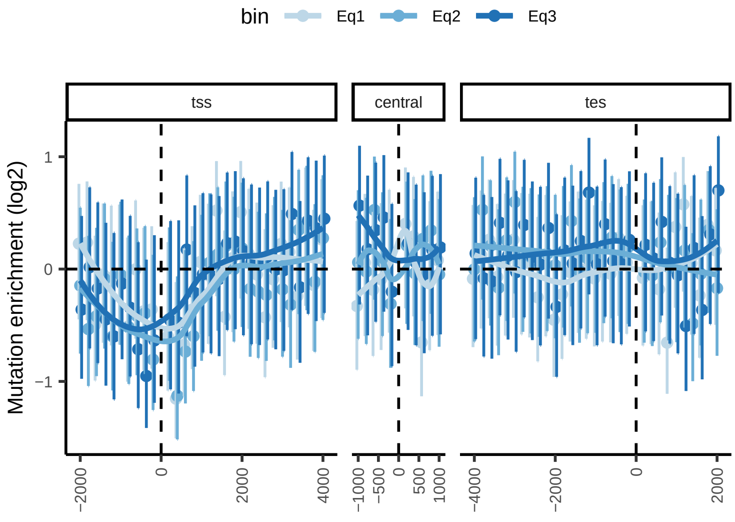

SBS17a

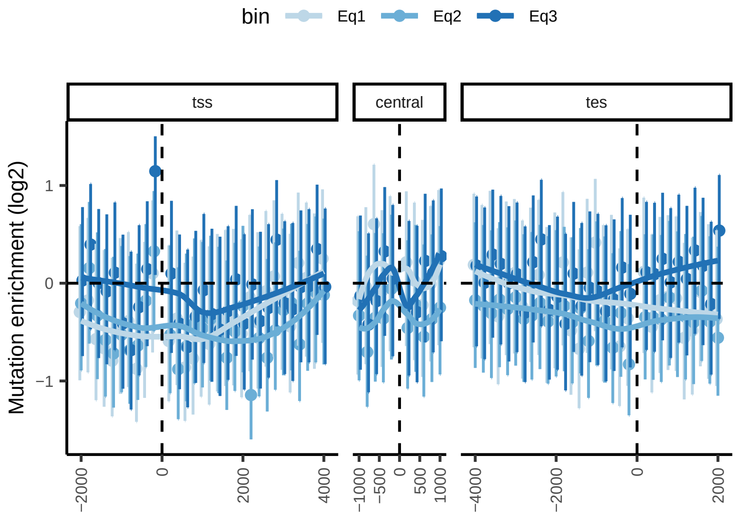

SBS17b

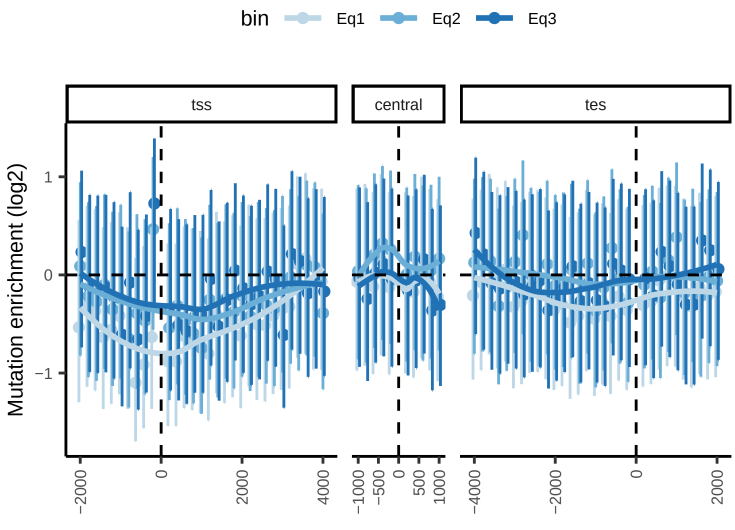

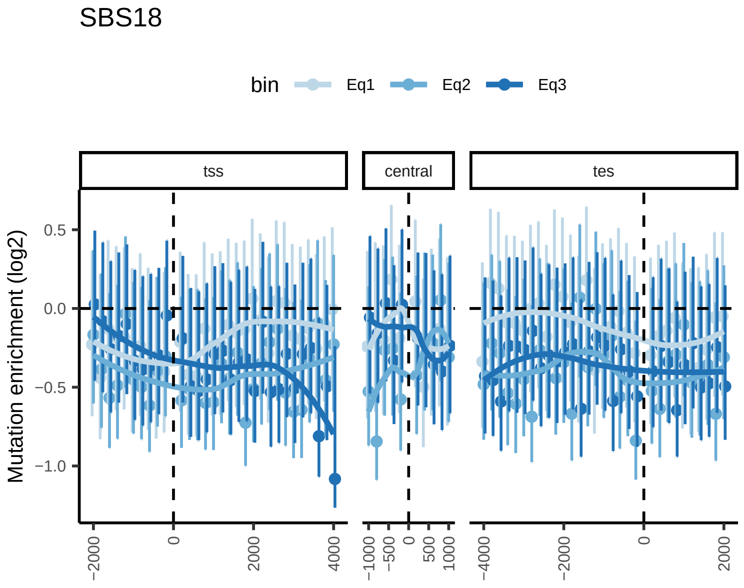
SBS18
bin
Eq1
Eq2
Eq3
tss
central
tes
Mutation enrichment (log2)
0.5
0.0
−0.5
−1.0
−2000
0
2000
4000
−1000
−500
0
500
1000
−4000
−2000
0
2000

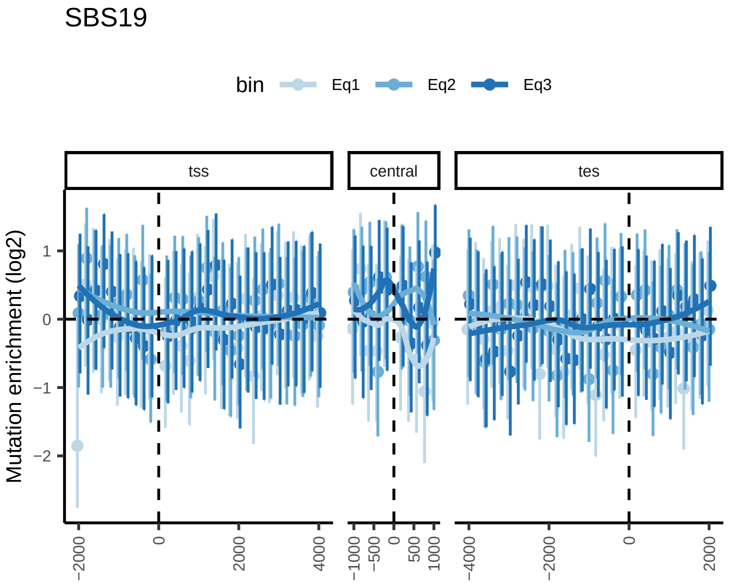
SBS19
bin
Eq1
Eq2
Eq3
tss
central
tes
Mutation enrichment (log2)
1
0
−1
−2
−2000
0
2000
4000
−1000
−500
0
500
1000
−4000
−2000
0
2000

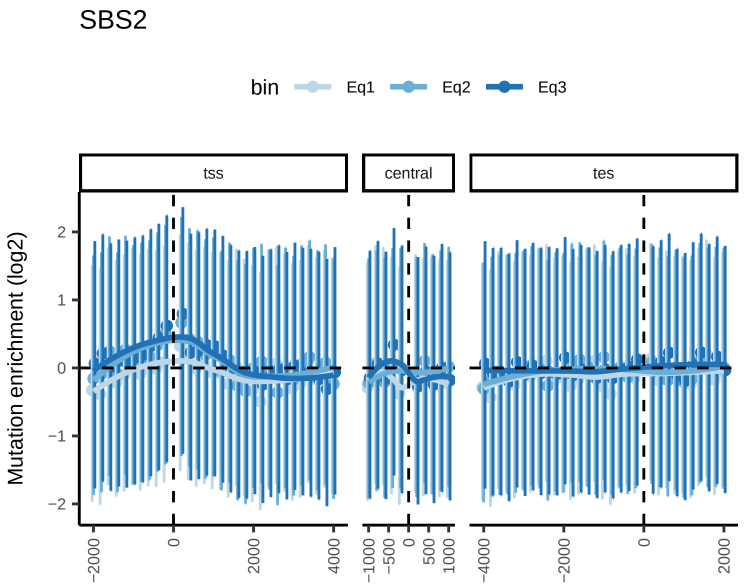
SBS2
bin
Eq1
Eq2
Eq3
tss
central
tes
Mutation enrichment (log2)
2
1
0
−1
−2
−2000
0
2000
4000
−1000
−500
0
500
1000
−4000
−2000
0
2000

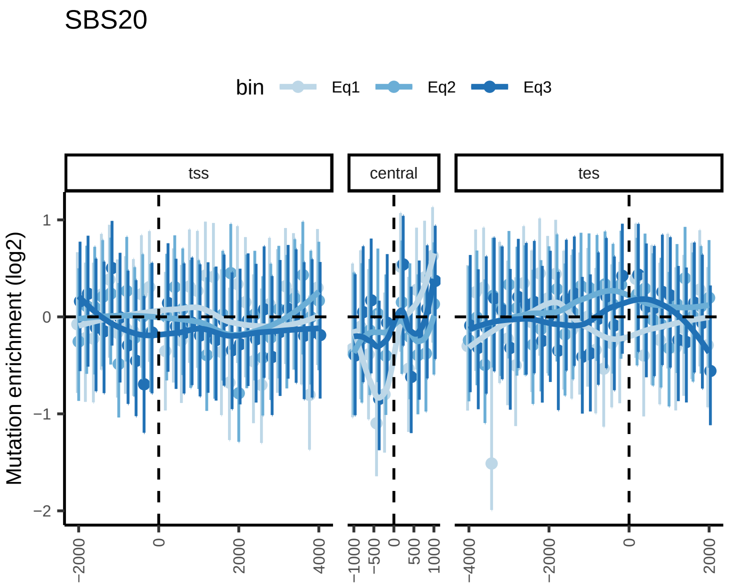
SBS20
bin
Eq1
Eq2
Eq3
tss
central
tes
Mutation enrichment (log2)
1
0
−1
−2
−2000
0
2000
4000
−1000
−500
0
500
1000
−4000
−2000
0
2000

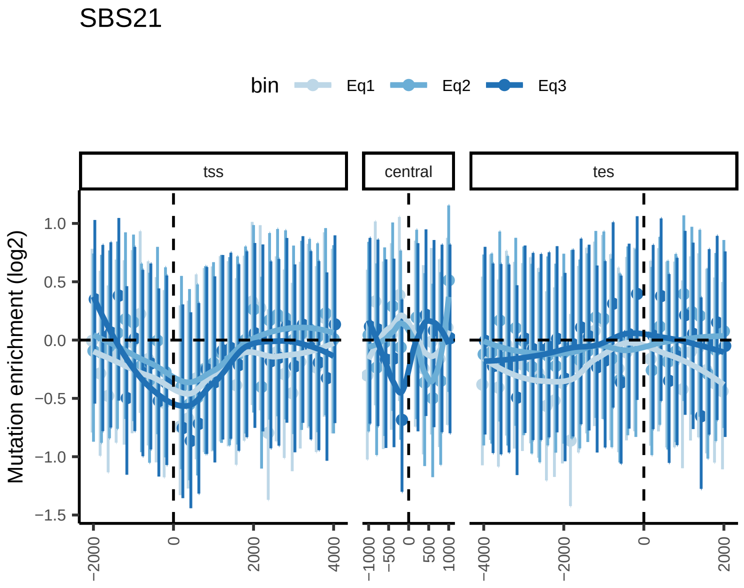
SBS21
bin
Eq1
Eq2
Eq3
tss
central
tes
Mutation enrichment (log2)
1.0
0.5
0.0
−0.5
−1.0
−1.5
−2000
0
2000
4000
−1000
−500
0
500
1000
−4000
−2000
0
2000

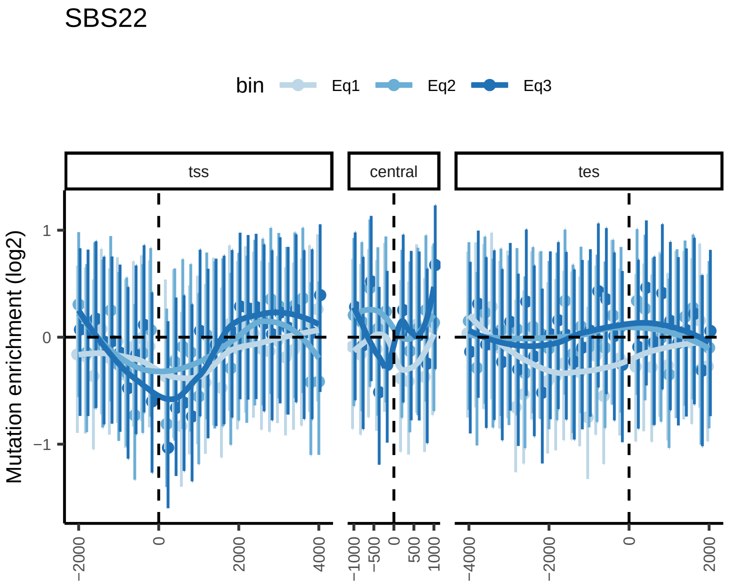
SBS22
bin
Eq1
Eq2
Eq3
tss
central
tes
Mutation enrichment (log2)
1
0
−1
−2000
0
2000
4000
−1000
−500
0
500
1000
−4000
−2000
0
2000

SBS24

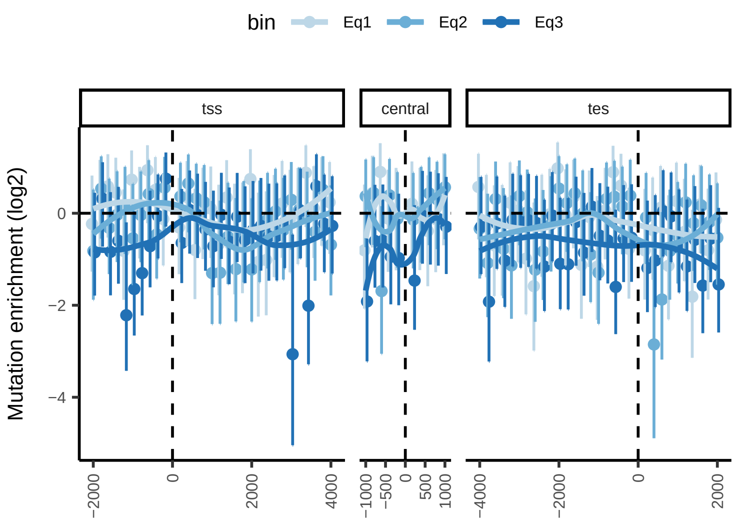

SBS25

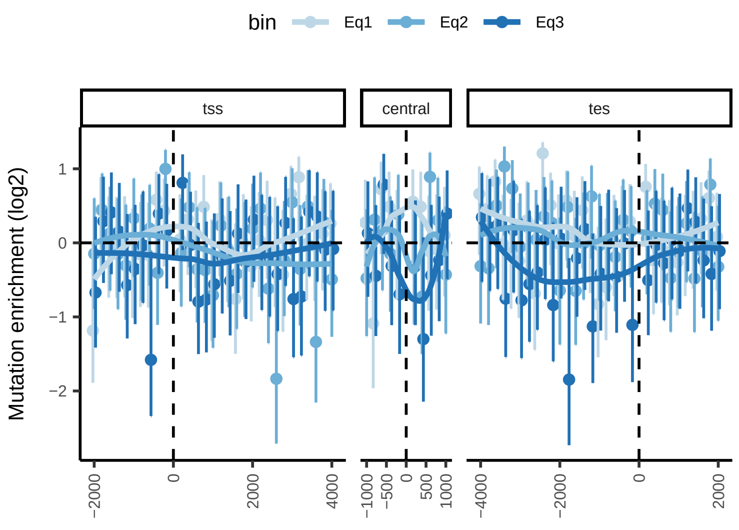

SBS26

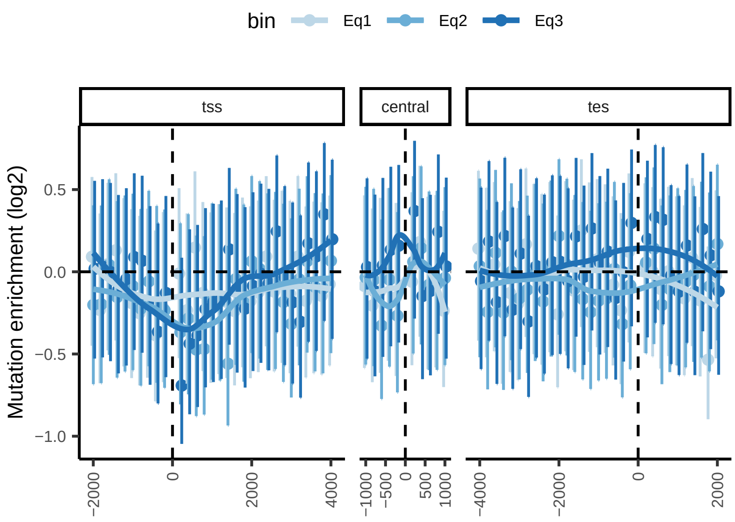

SBS28

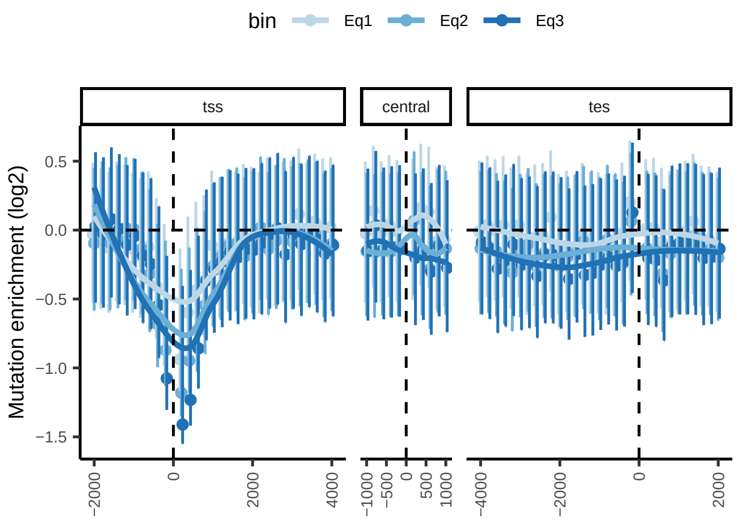

SBS3

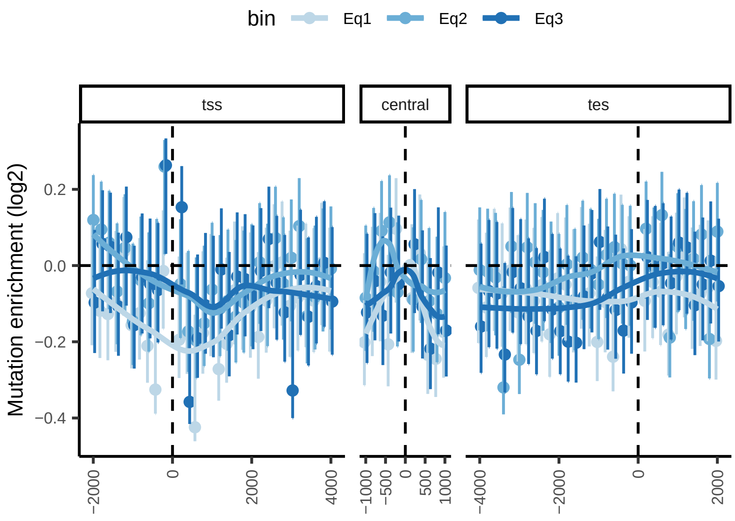

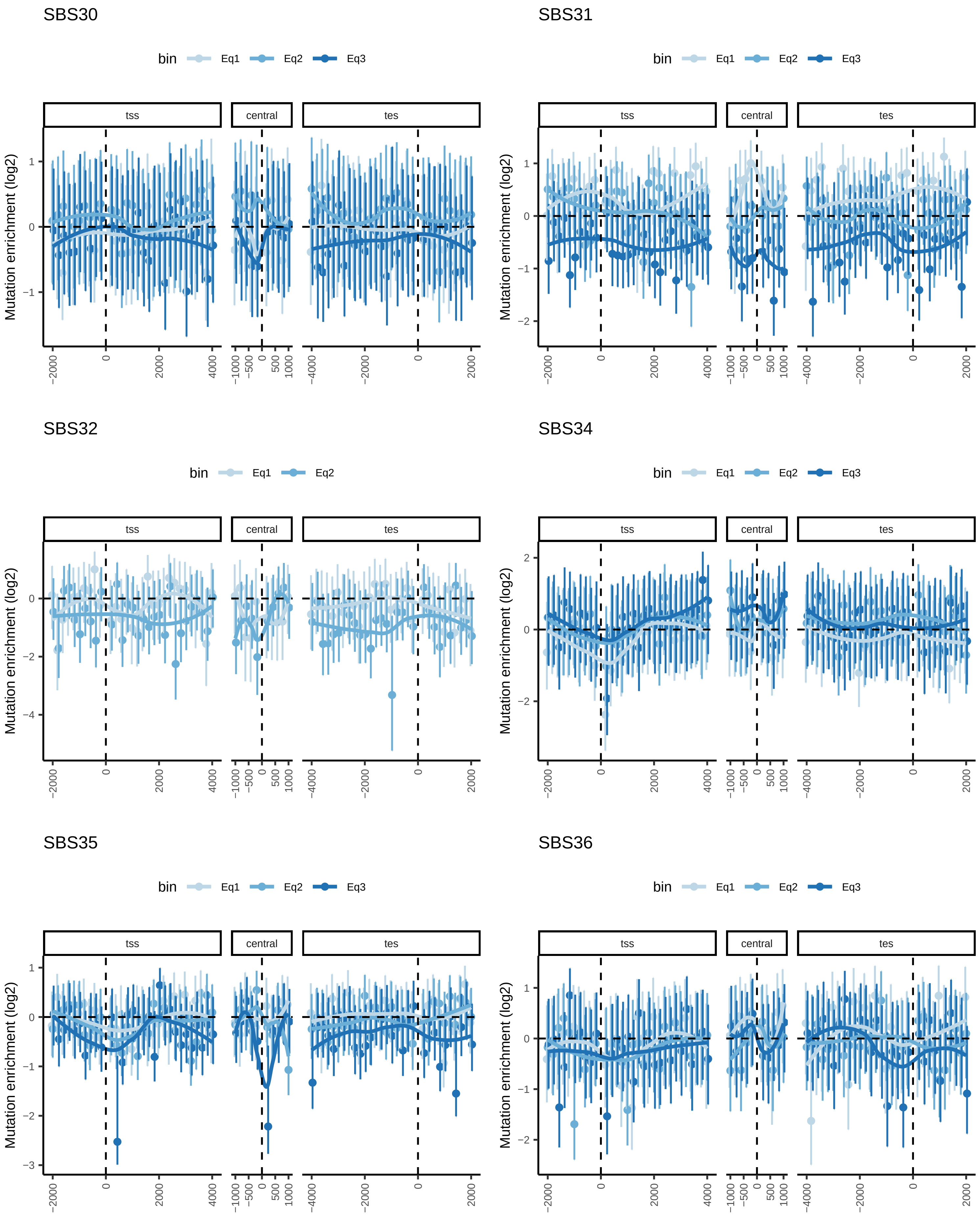
SBS30
bin Eq1 Eq2 Eq3
tss
central
tes
Mutation enrichment (log2)
SBS31
bin Eq1 Eq2 Eq3
tss
central
tes
Mutation enrichment (log2)
SBS32
bin Eq1 Eq2
tss
central
tes
Mutation enrichment (log2)
SBS34
bin Eq1 Eq2 Eq3
tss
central
tes
Mutation enrichment (log2)
SBS35
bin Eq1 Eq2 Eq3
tss
central
tes
Mutation enrichment (log2)
SBS36
bin Eq1 Eq2 Eq3
tss
central
tes
Mutation enrichment (log2)

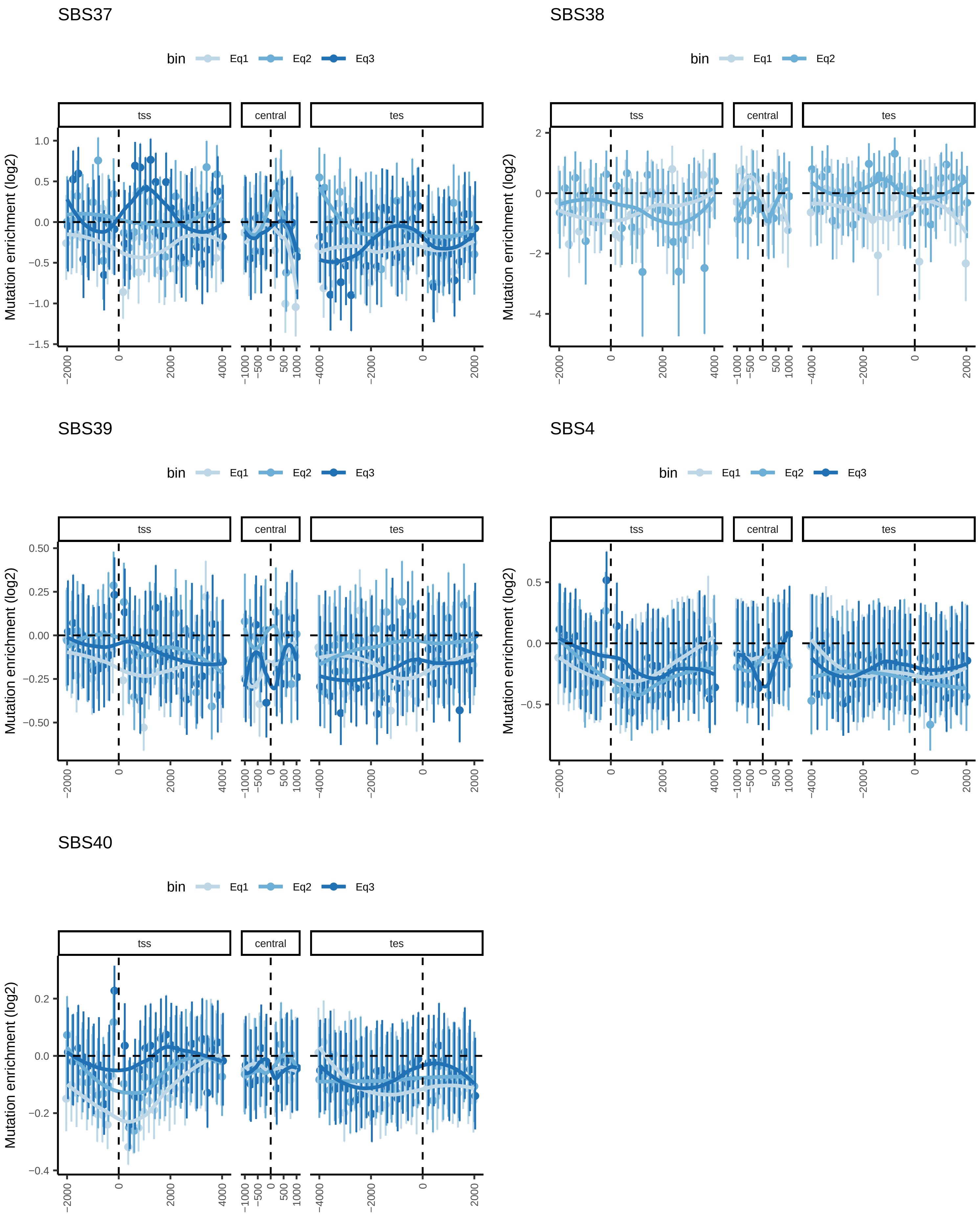

SBS37
bin Eq1 Eq2 Eq3
tss
central
tes
Mutation enrichment (log2)
SBS38
bin Eq1 Eq2
tss
central
tes
Mutation enrichment (log2)
SBS39
bin Eq1 Eq2 Eq3
tss
central
tes
Mutation enrichment (log2)
SBS4
bin Eq1 Eq2 Eq3
tss
central
tes
Mutation enrichment (log2)
SBS40
bin Eq1 Eq2 Eq3
tss
central
tes
Mutation enrichment (log2)

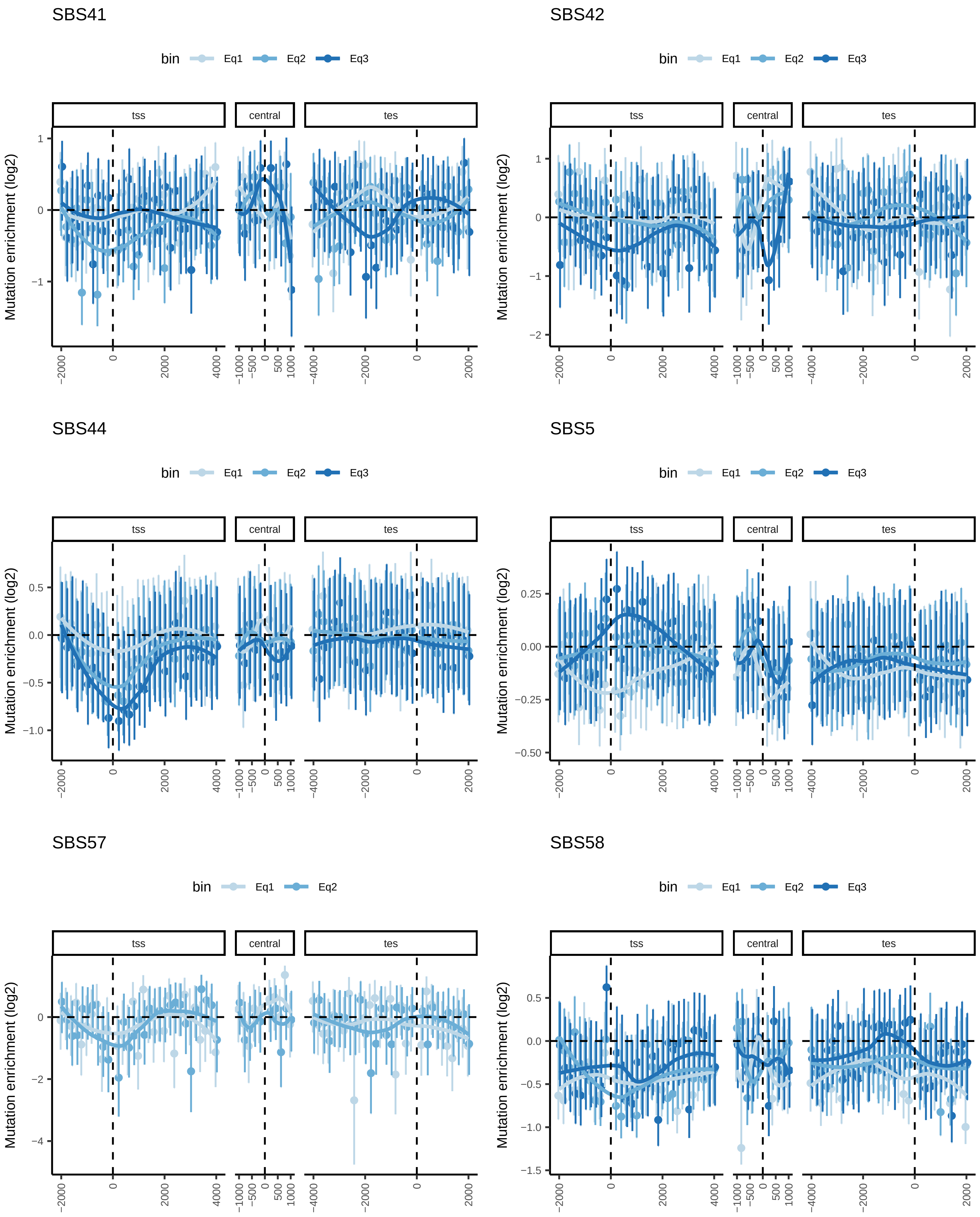
SBS41
bin Eq1 Eq2 Eq3
tss
central
tes
Mutation enrichment (log2)
1
0
−1
−2000
0
2000
4000
−1000
−500
0
500
1000
−4000
−2000
0
2000
SBS42
bin Eq1 Eq2 Eq3
tss
central
tes
Mutation enrichment (log2)
1
0
−1
−2
SBS44
bin Eq1 Eq2 Eq3
tss
central
tes
Mutation enrichment (log2)
0.5
0.0
−0.5
−1.0
SBS5
bin Eq1 Eq2 Eq3
tss
central
tes
Mutation enrichment (log2)
0.25
0.00
−0.25
−0.50
SBS57
bin Eq1 Eq2
tss
central
tes
Mutation enrichment (log2)
0
−2
−4
SBS58
bin Eq1 Eq2 Eq3
tss
central
tes
Mutation enrichment (log2)
0.5
0.0
−0.5
−1.0
−1.5

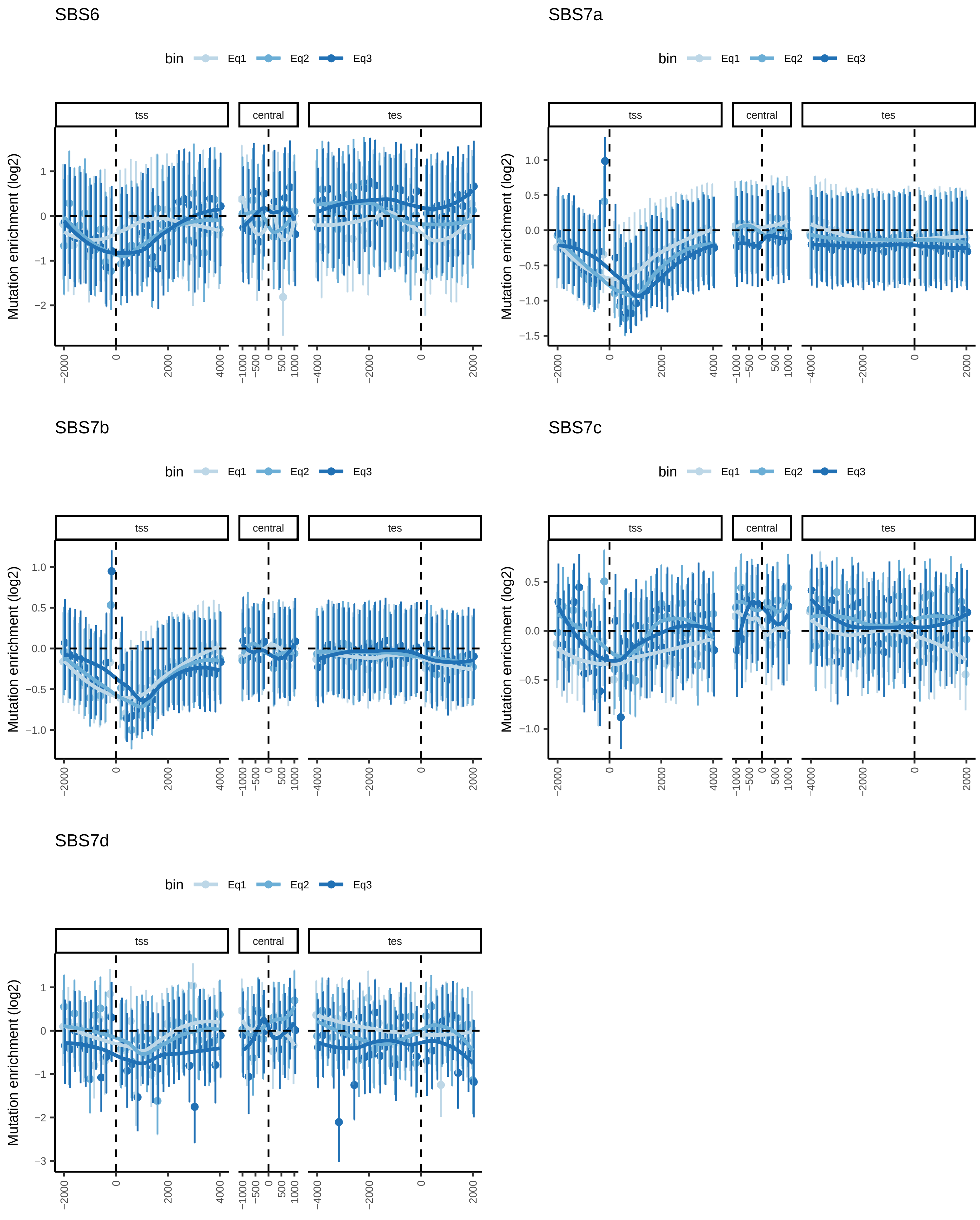
SBS6
SBS7a
SBS7b
SBS7c
SBS7d
bin
Eq1
Eq2
Eq3
tss
central
tes
Mutation enrichment (log2)
−2000
0
2000
4000
−1000
−500
500
1000
−4000
1.0
0.5
0.0
−0.5
−1.0
−1.5
1
−1
−2
−3

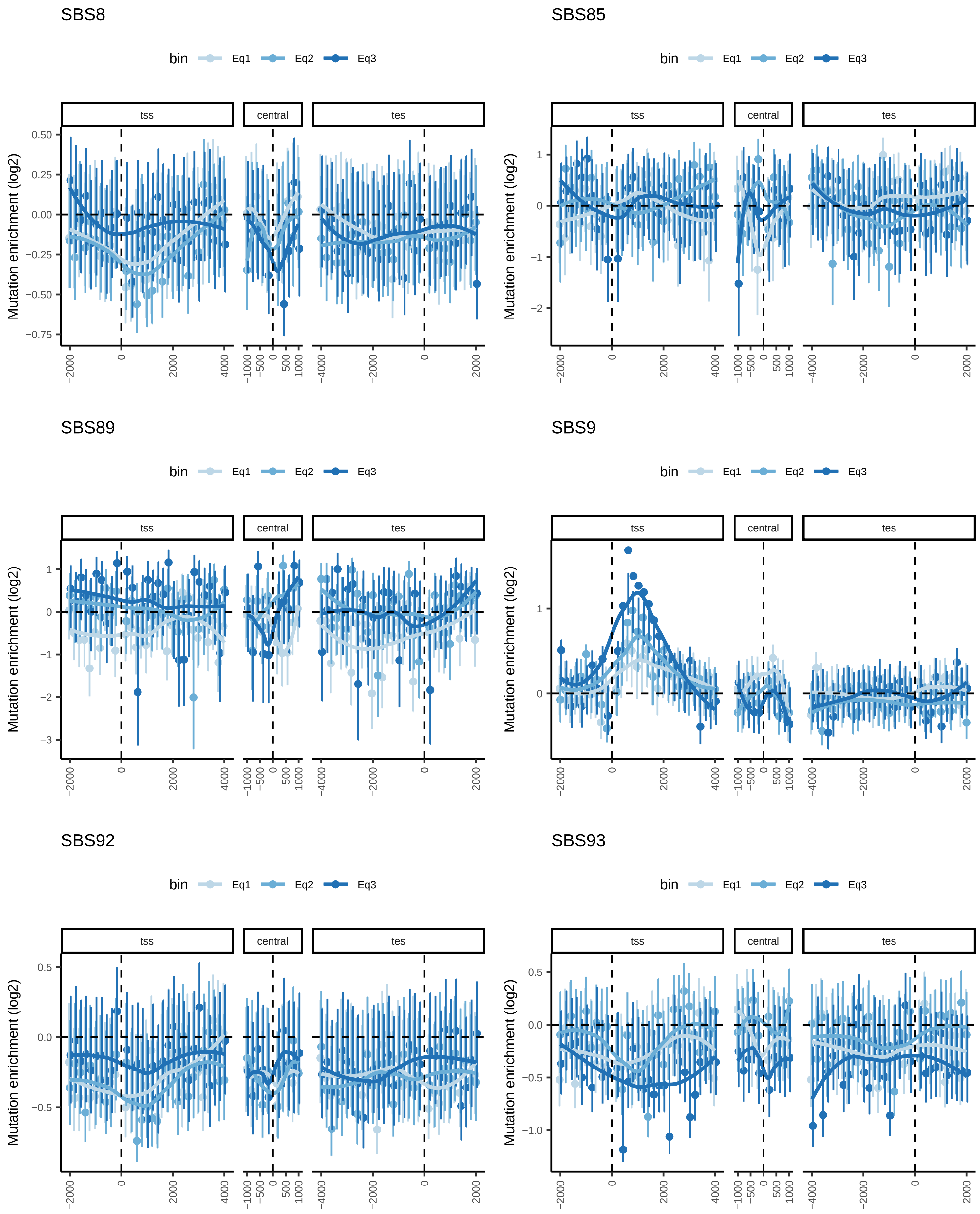

SBS8
SBS85
SBS89
SBS9
SBS92
SBS93
bin
Eq1
Eq2
Eq3
tss
central
tes
Mutation enrichment (log2)
0.50
0.25
0.00
−0.25
−0.50
−0.75
1
0
−1
−2
−3
0.5
0.0
−0.5
−1.0
−2000
0
2000
4000
−1000
−500
500
1000
−4000
