# Supplementary material for: Mutation rate heterogeneity at the sub-gene scale due to local DNA hypomethylation
Source: Nucleic Acids Res. 2024 Apr 8;52(8):4393–408. doi: 10.1093/nar/gkae252 (PMC11077091; doi:10.1093/nar/gkae252)
Supplement: gkae252_Supplemental_Files [file gkae252_supplemental_files.zip › Supplementary_Figure_S11.pdf]

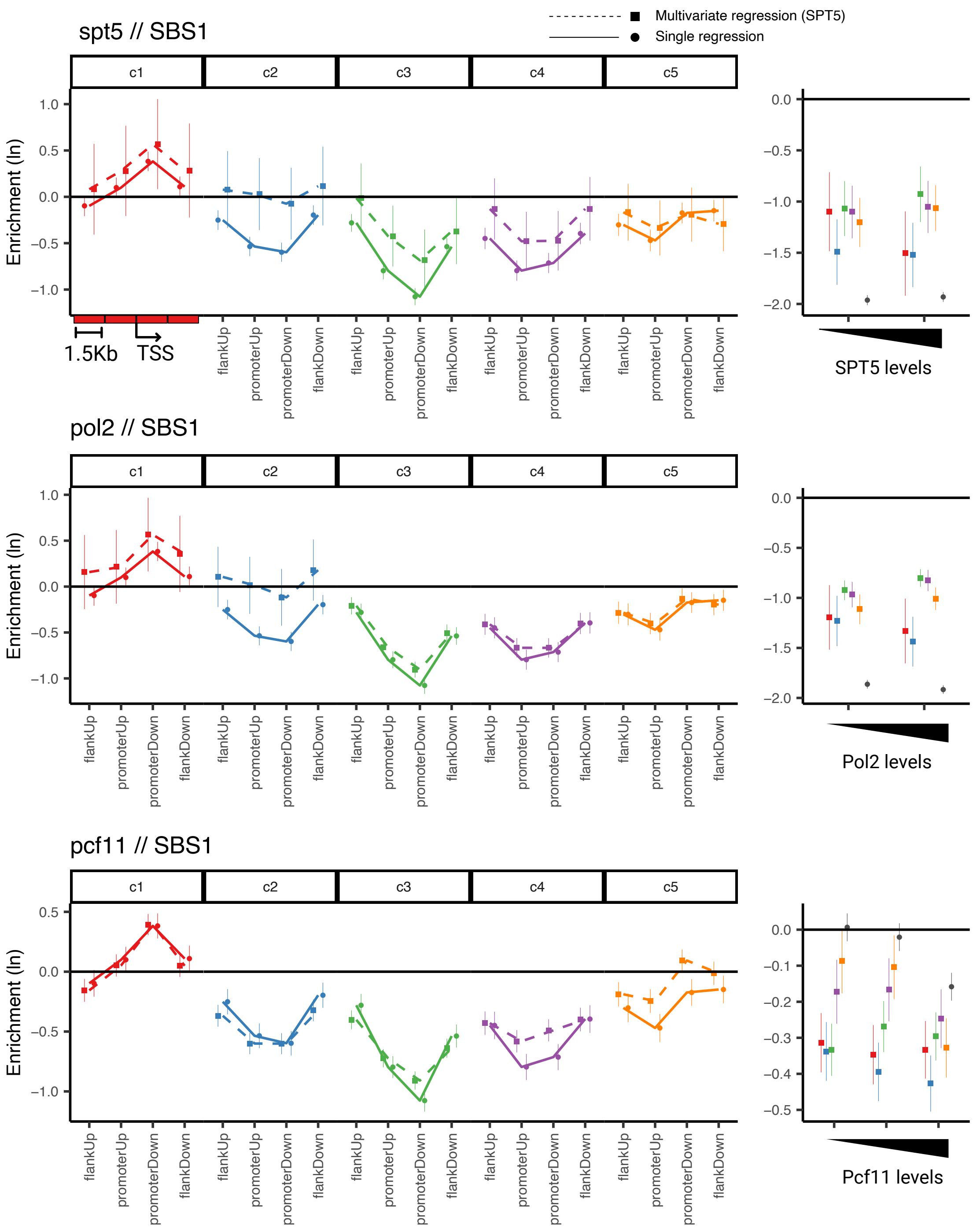

spt5 // SBS1
Multivariate regression (SPT5)
Single regression
c1
c2
c3
c4
c5
Enrichment (ln)
1.5Kb
TSS
flankUp
promoterUp
promoterDown
flankDown
SPT5 levels
pol2 // SBS1
Pol2 levels
pcf11 // SBS1
Pcf11 levels

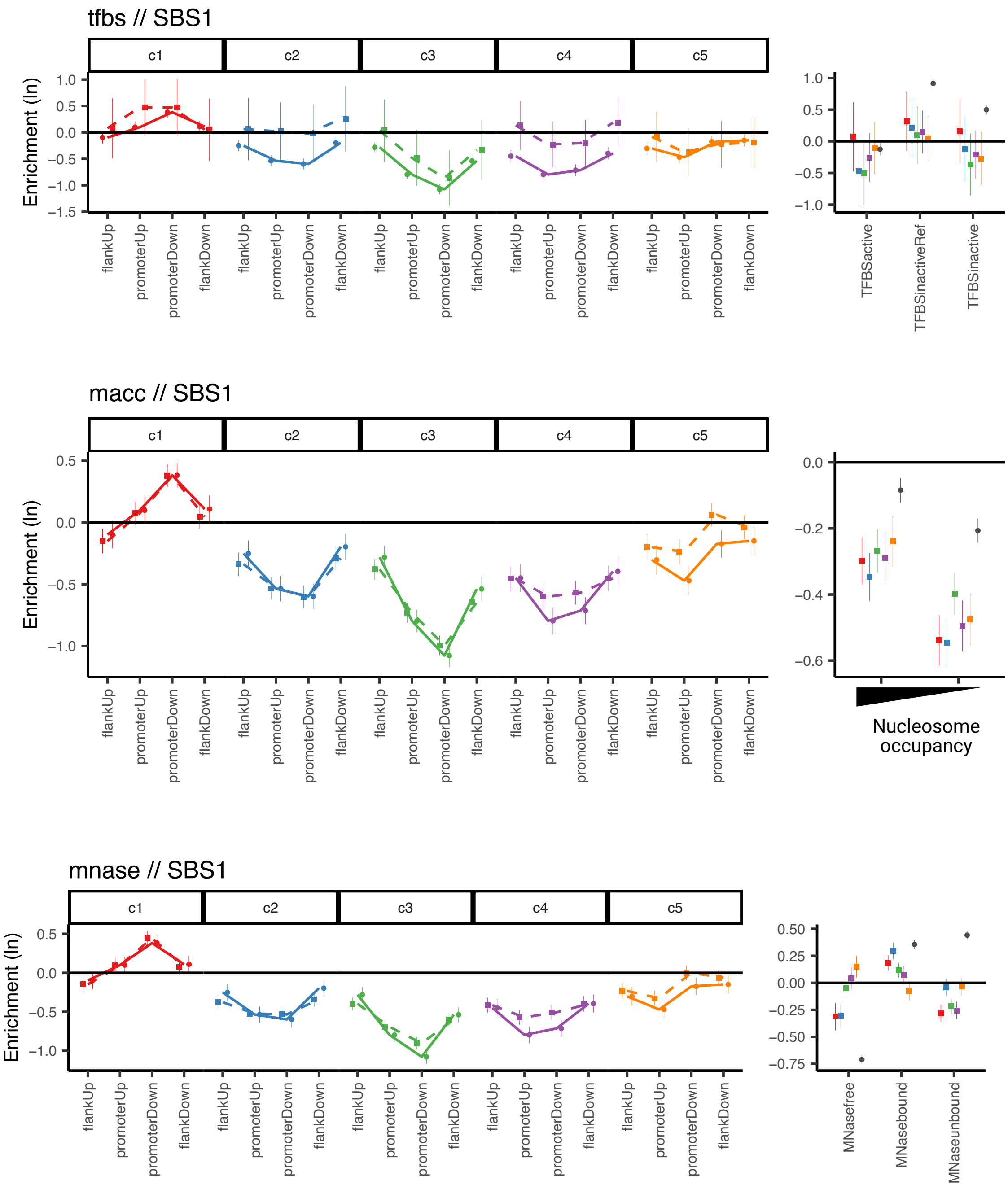

tfbs // SBS1
c1
c2
c3
c4
c5
Enrichment (ln)
flankUp
promoterUp
promoterDown
flankDown
TFBSactive
TFBSinactiveRef
TFBSinactive
macc // SBS1
Nucleosome occupancy
mnase // SBS1
MNasefree
MNasebound
MNaseunbound

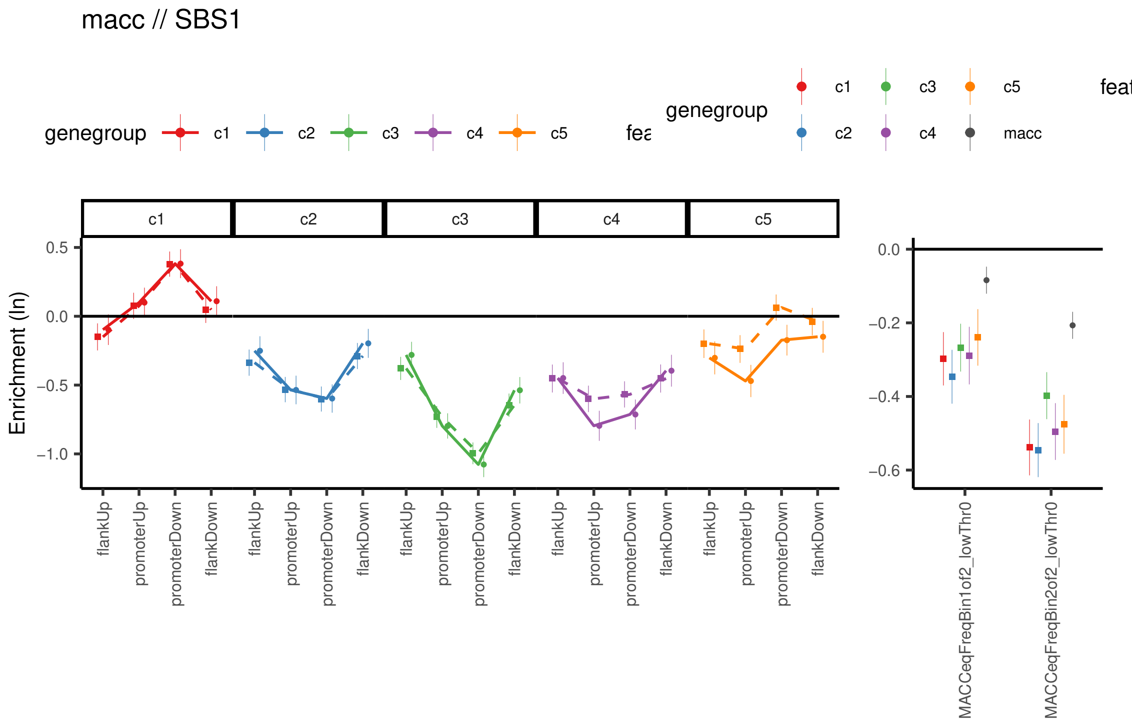

macc // SBS1
genegroup c1 c2 c3 c4 c5
fea
genegroup c1 c3 c5 c2 c4 macc
feat
c1
c2
c3
c4
c5
Enrichment (ln)
0.5
0.0
−0.5
−1.0
flankUp
promoterUp
promoterDown
flankDown
0.0
−0.2
−0.4
−0.6
MACCeqFreqBin1of2_lowThr0
MACCeqFreqBin2of2_lowThr0

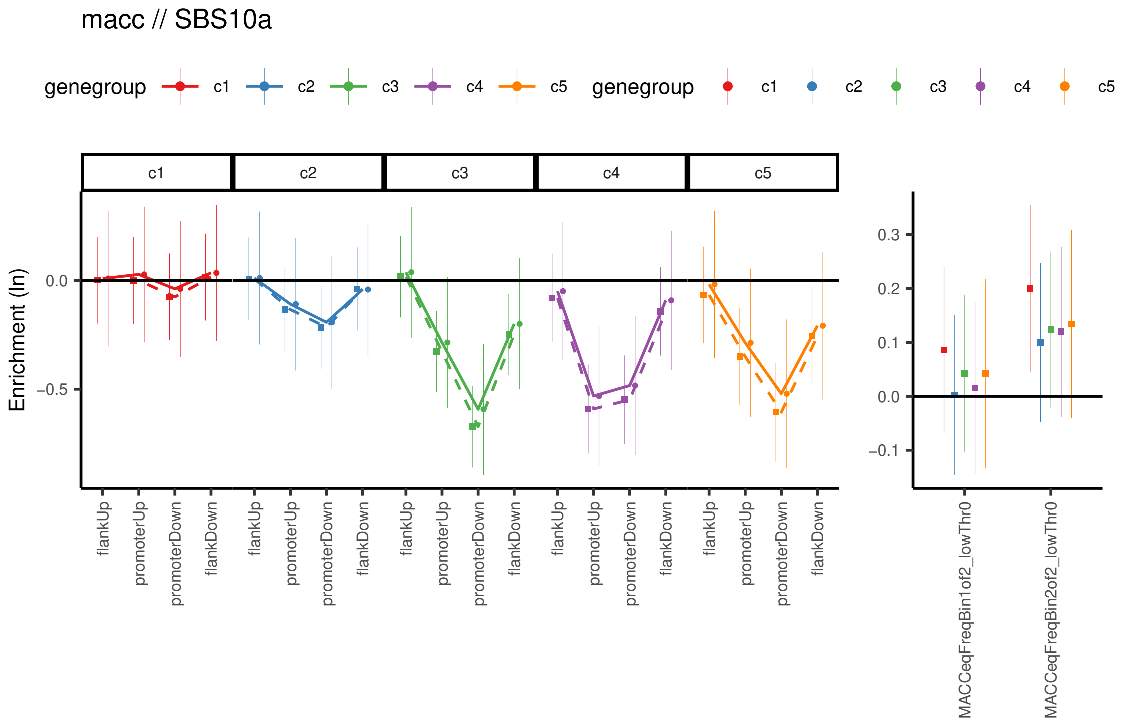

macc // SBS10a
genegroup
c1
c2
c3
c4
c5
genegroup
c1
c2
c3
c4
c5
Enrichment (ln)
0.0
−0.5
flankUp
promoterUp
promoterDown
flankDown
0.3
0.2
0.1
0.0
−0.1
MACCeqFreqBin1of2_lowThr0
MACCeqFreqBin2of2_lowThr0

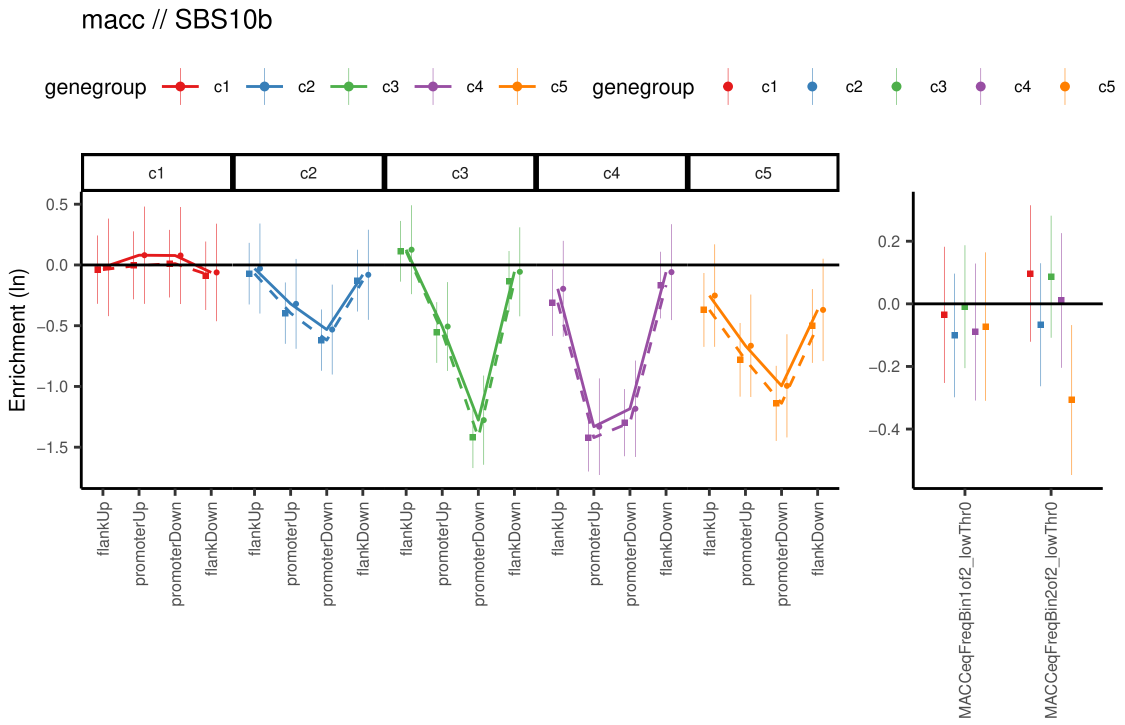

macc // SBS10b
genegroup
c1
c2
c3
c4
c5
genegroup
c1
c2
c3
c4
c5
c1
c2
c3
c4
c5
Enrichment (ln)
0.5
0.0
−0.5
−1.0
−1.5
flankUp
promoterUp
promoterDown
flankDown
0.2
0.0
−0.2
−0.4
MACCeqFreqBin1of2_lowThr0
MACCeqFreqBin2of2_lowThr0

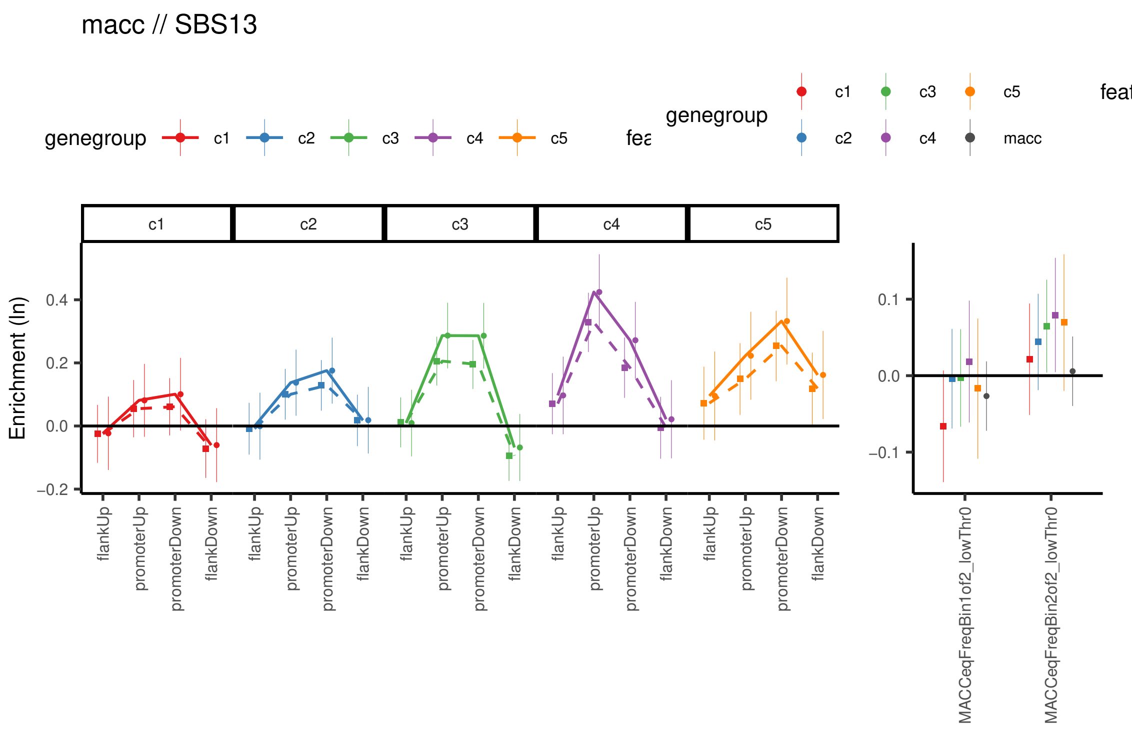

macc // SBS13
genegroup
c1
c2
c3
c4
c5
fea
genegroup
c1
c3
c5
c2
c4
macc
feat
Enrichment (ln)
0.4
0.2
0.0
−0.2
0.1
0.0
−0.1
flankUp
promoterUp
promoterDown
flankDown
MACCeqFreqBin1of2_lowThr0
MACCeqFreqBin2of2_lowThr0

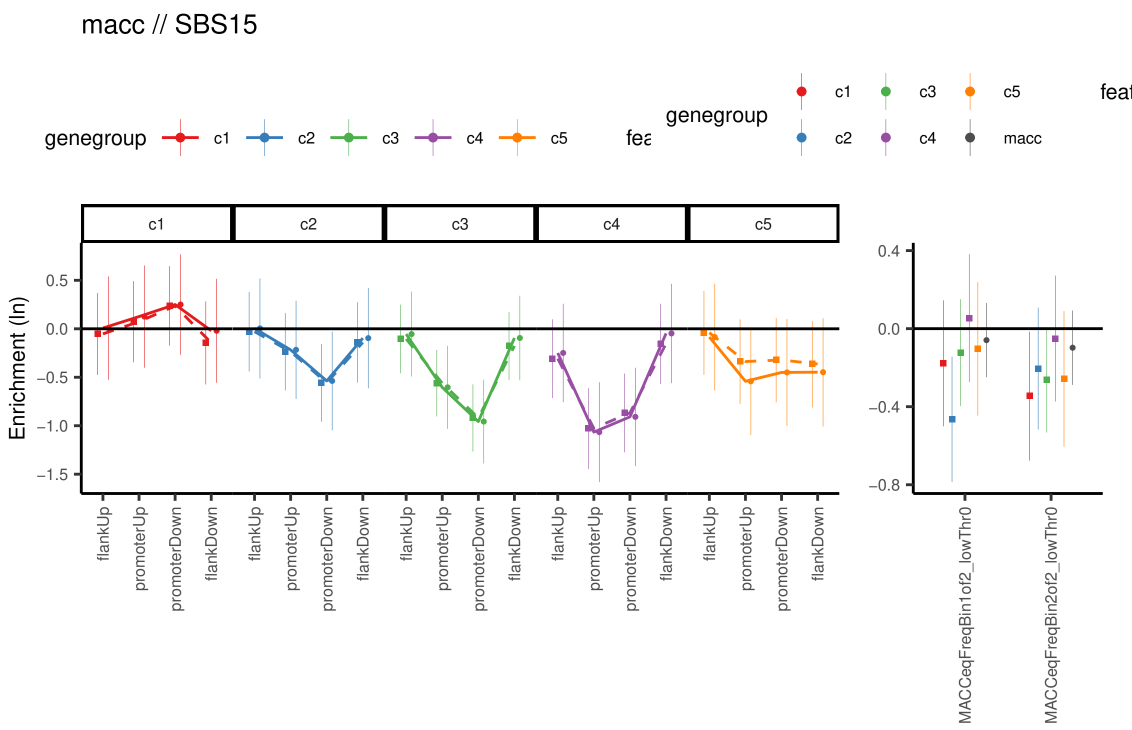

macc // SBS15
genegroup
c1
c2
c3
c4
c5
fea
genegroup
c1
c3
c5
c2
c4
macc
feat
Enrichment (ln)
0.5
0.0
−0.5
−1.0
−1.5
flankUp
promoterUp
promoterDown
flankDown
0.4
0.0
−0.4
−0.8
MACCeqFreqBin1of2_lowThr0
MACCeqFreqBin2of2_lowThr0

macc // SBS2

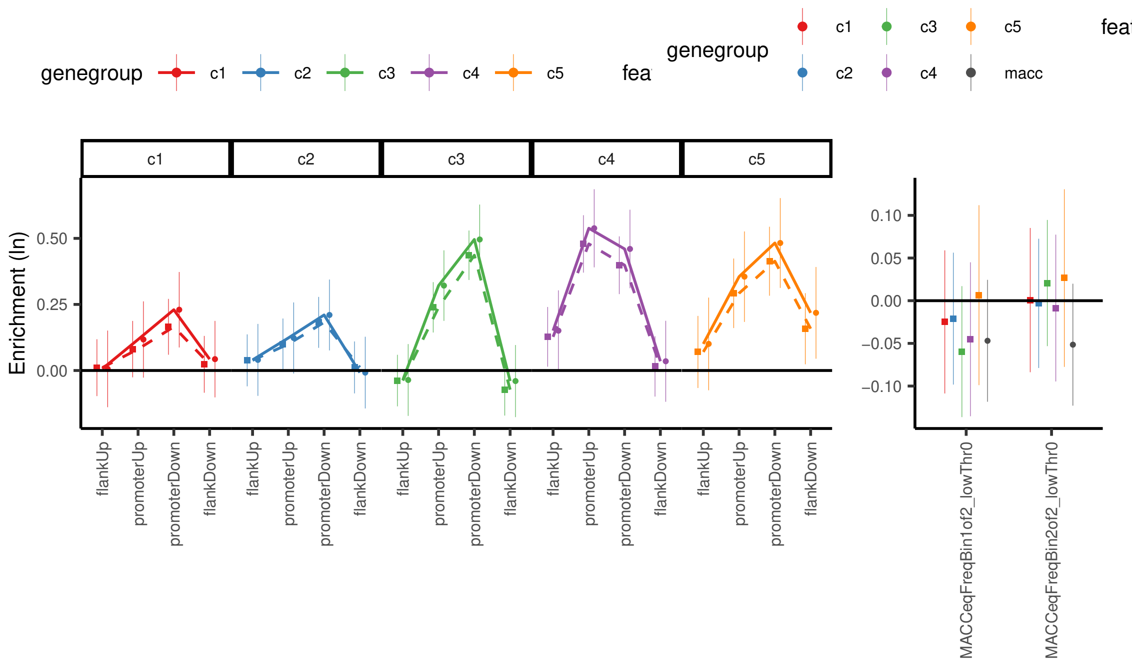

genegroup
c1
c2
c3
c4
c5
fea
genegroup
c1
c3
c5
c2
c4
macc
fea
Enrichment (ln)
0.50
0.25
0.00
c1
c2
c3
c4
c5
flankUp
promoterUp
promoterDown
flankDown
0.10
0.05
0.00
−0.05
−0.10
MACCeqFreqBin1of2_lowThr0
MACCeqFreqBin2of2_lowThr0

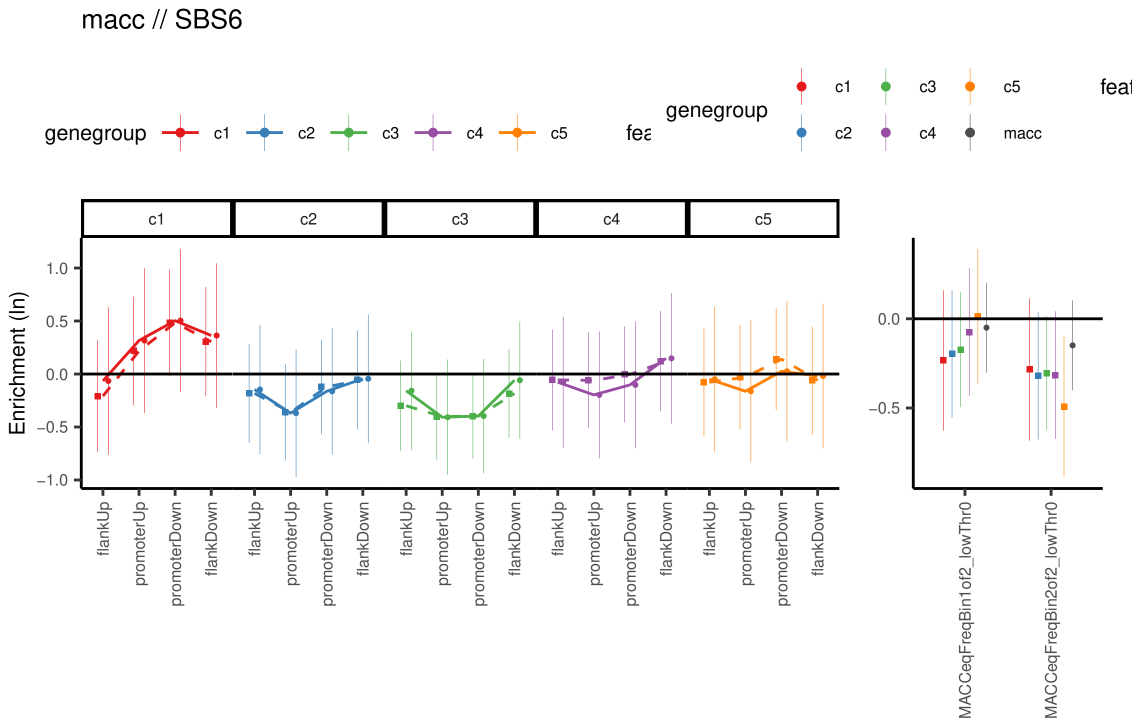
macc // SBS6
genegroup
c1
c2
c3
c4
c5
fea
genegroup
c1
c3
c5
c2
c4
macc
feat
c1
c2
c3
c4
c5
Enrichment (ln)
1.0
0.5
0.0
−0.5
−1.0
flankUp
promoterUp
promoterDown
flankDown
0.0
−0.5
MACCeqFreqBin1of2_lowThr0
MACCeqFreqBin2of2_lowThr0

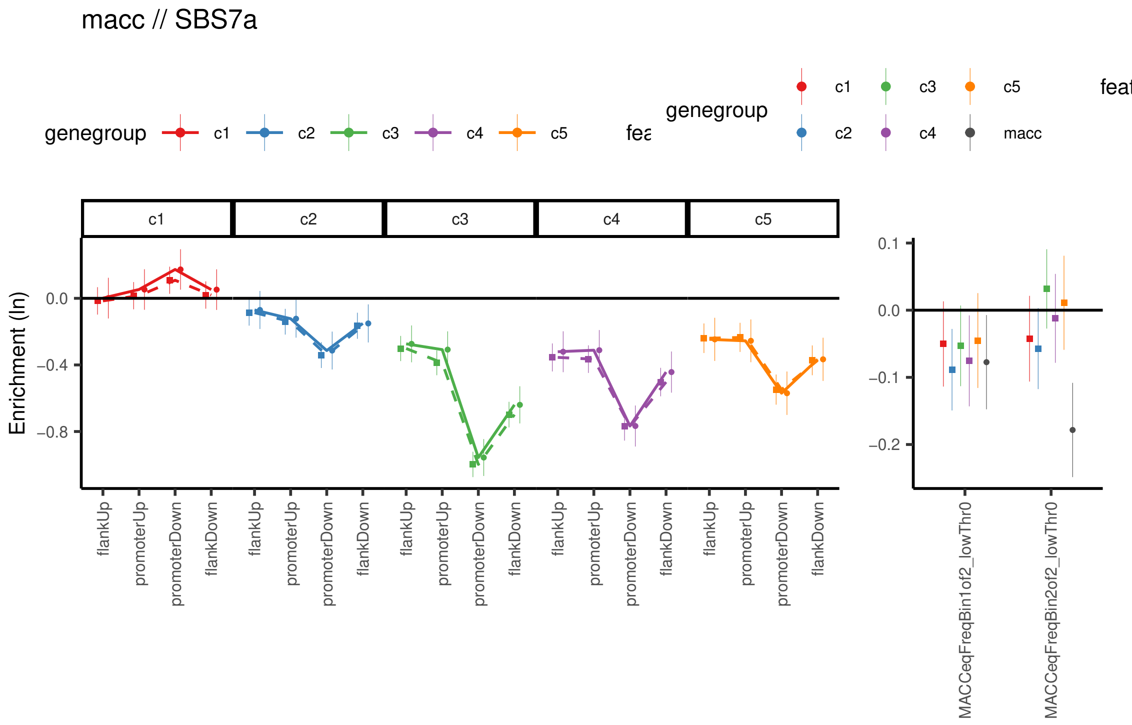

macc // SBS7a
genegroup
c1
c2
c3
c4
c5
fea
genegroup
c1
c3
c5
c2
c4
macc
feat
Enrichment (ln)
0.0
−0.4
−0.8
c1
c2
c3
c4
c5
flankUp
promoterUp
promoterDown
flankDown
0.1
0.0
−0.1
−0.2
MACCeqFreqBin1of2_lowThr0
MACCeqFreqBin2of2_lowThr0

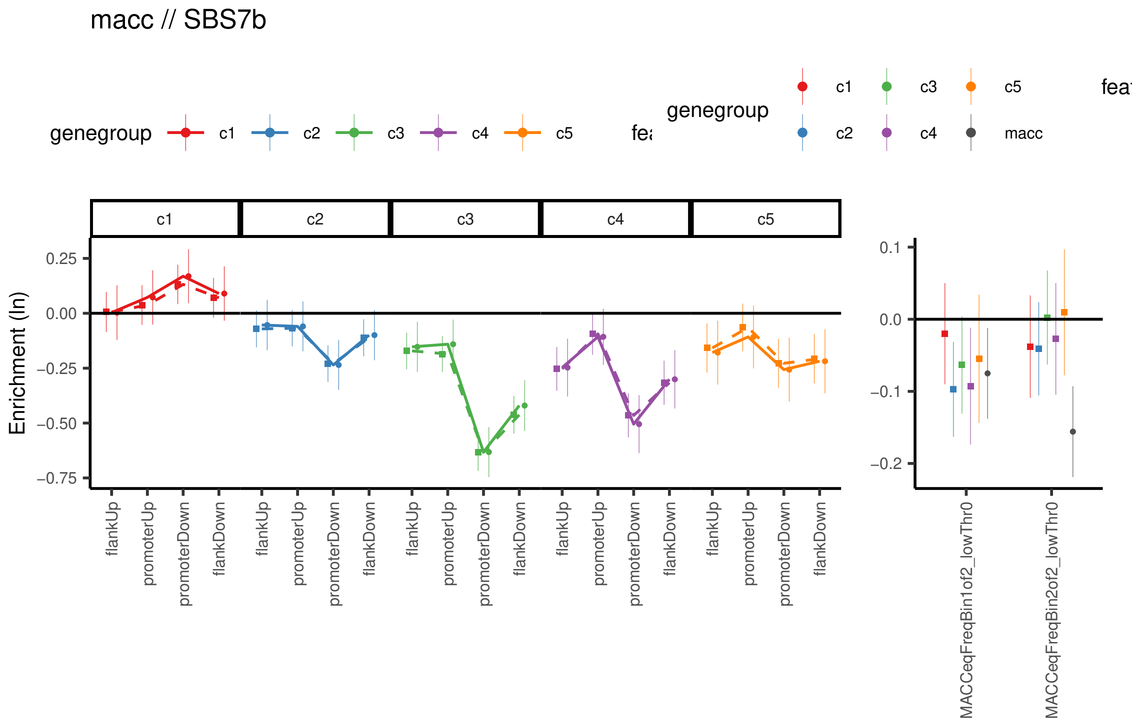

macc // SBS7b
genegroup
c1
c2
c3
c4
c5
genegroup
c1
c3
c5
c2
c4
macc
c1
c2
c3
c4
c5
Enrichment (ln)
0.25
0.00
−0.25
−0.50
−0.75
flankUp
promoterUp
promoterDown
flankDown
0.1
0.0
−0.1
−0.2
MACCeqFreqBin1of2_lowThr0
MACCeqFreqBin2of2_lowThr0

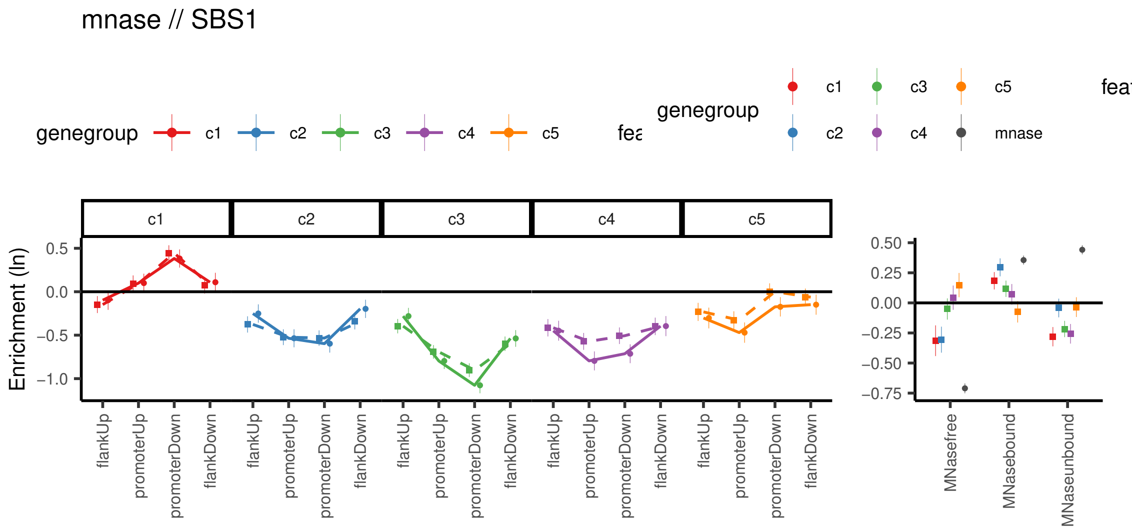
mnase // SBS1
genegroup c1 c2 c3 c4 c5
genegroup c1 c2 c3 c4 c5 mnase
c1
c2
c3
c4
c5
Enrichment (ln)
0.5
0.0
−0.5
−1.0
flankUp
promoterUp
promoterDown
flankDown
0.50
0.25
0.00
−0.25
−0.50
−0.75
MNasefree
MNasebound
MNaseunbound

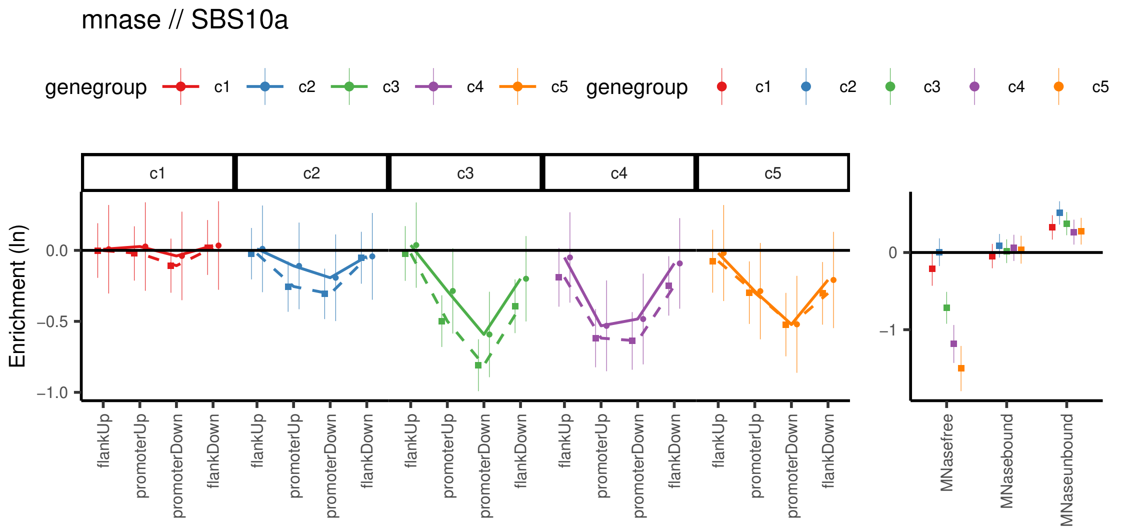
mnase // SBS10a
genegroup
c1
c2
c3
c4
c5
genegroup
c1
c2
c3
c4
c5
c1
c2
c3
c4
c5
Enrichment (ln)
0.0
−0.5
−1.0
0
−1
flankUp
promoterUp
promoterDown
flankDown
MNasefree
MNasebound
MNaseunbound

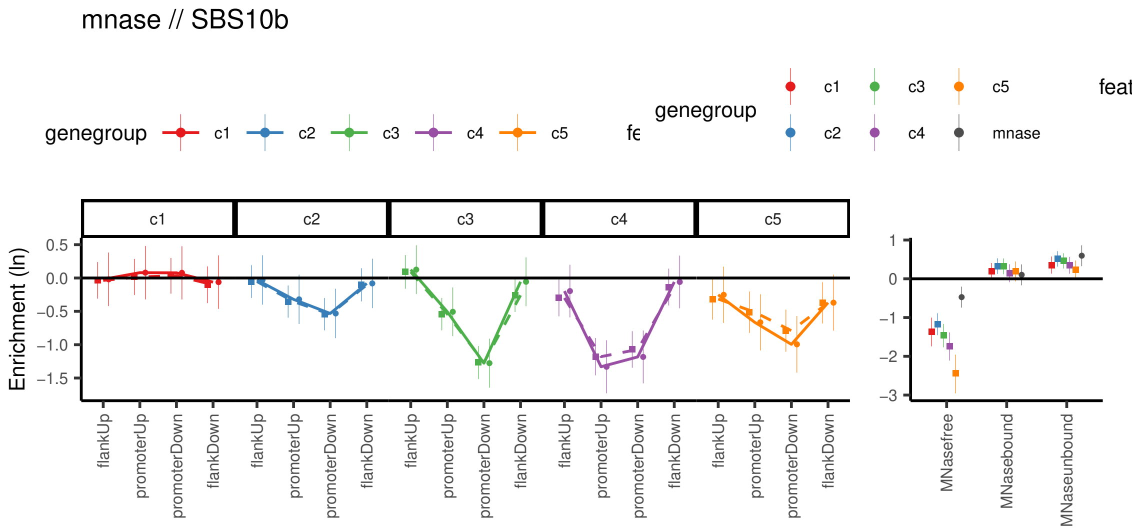
mnase // SBS10b
genegroup
c1
c2
c3
c4
c5
genegroup
c1
c3
c5
c2
c4
mnase
c1
c2
c3
c4
c5
Enrichment (ln)
0.5
0.0
−0.5
−1.0
−1.5
flankUp
promoterUp
promoterDown
flankDown
1
0
−1
−2
−3
MNasefree
MNasebound
MNaseunbound

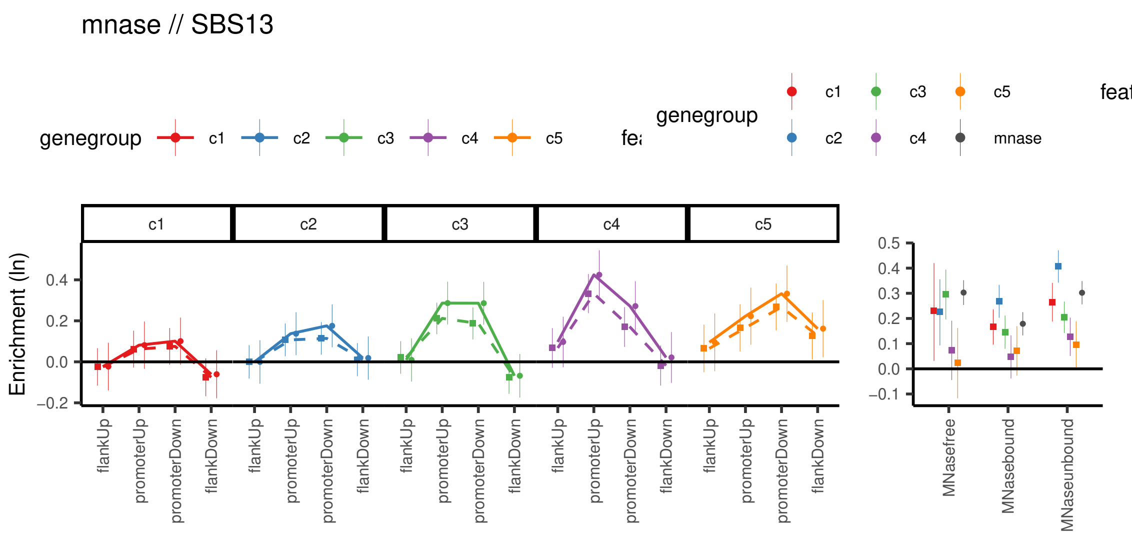
mnase // SBS13
genegroup
c1
c2
c3
c4
c5
fea
genegroup
c1
c3
c5
c2
c4
mnase
feat
Enrichment (ln)
0.4
0.2
0.0
−0.2
flankUp
promoterUp
promoterDown
flankDown
0.5
0.4
0.3
0.2
0.1
0.0
−0.1
MNasefree
MNasebound
MNaseunbound

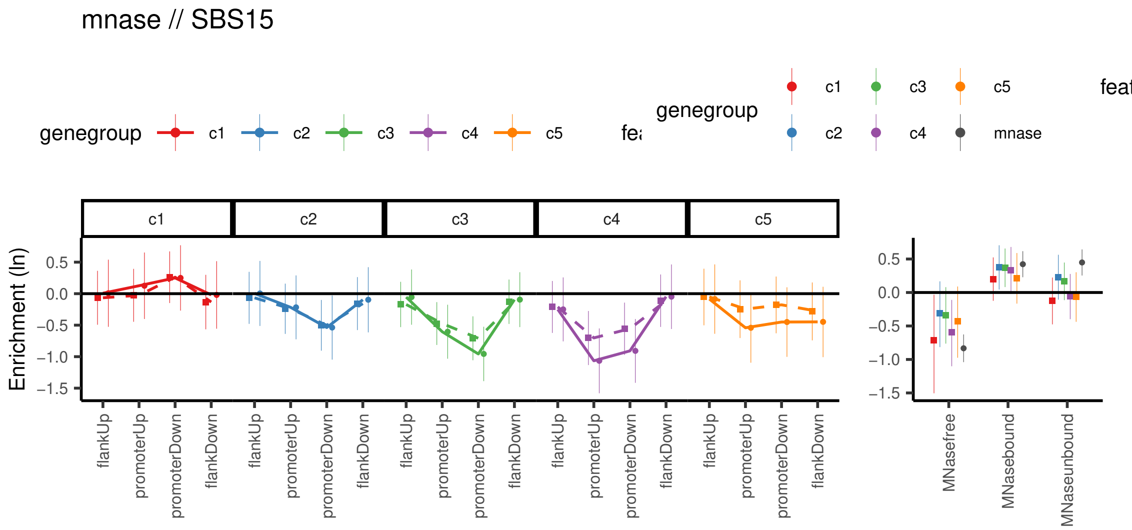

mnase // SBS15
genegroup
c1
c2
c3
c4
c5
fea
genegroup
c1
c3
c5
c2
c4
mnase
feat
c1
c2
c3
c4
c5
Enrichment (ln)
0.5
0.0
−0.5
−1.0
−1.5
flankUp
promoterUp
promoterDown
flankDown
MNasefree
MNasebound
MNaseunbound

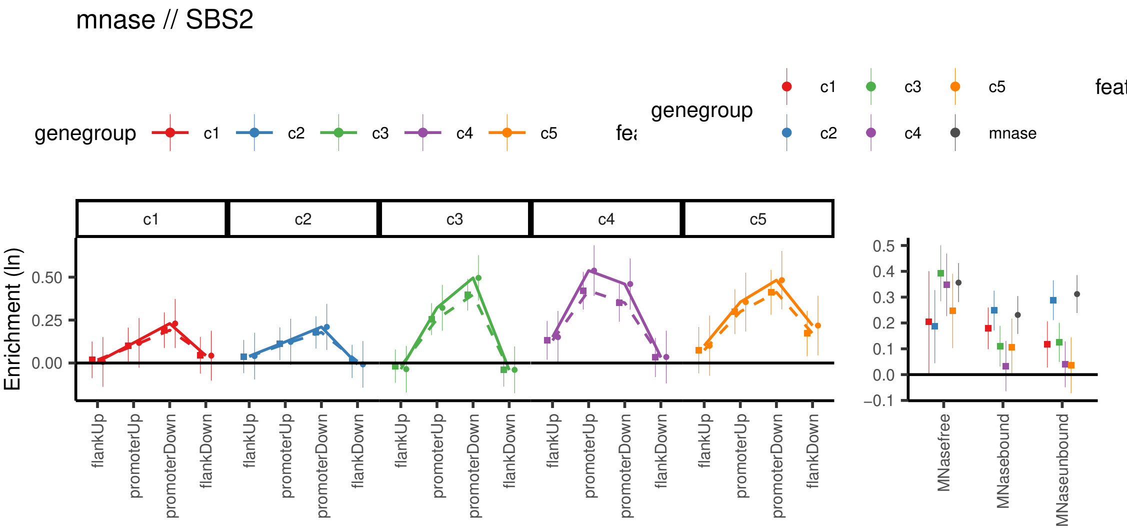

mnase // SBS2
genegroup
c1
c2
c3
c4
c5
fea
genegroup
c1
c3
c5
c2
c4
mnase
feat
Enrichment (ln)
0.50
0.25
0.00
flankUp
promoterUp
promoterDown
flankDown
0.5
0.4
0.3
0.2
0.1
0.0
−0.1
MNasefree
MNasebound
MNaseunbound

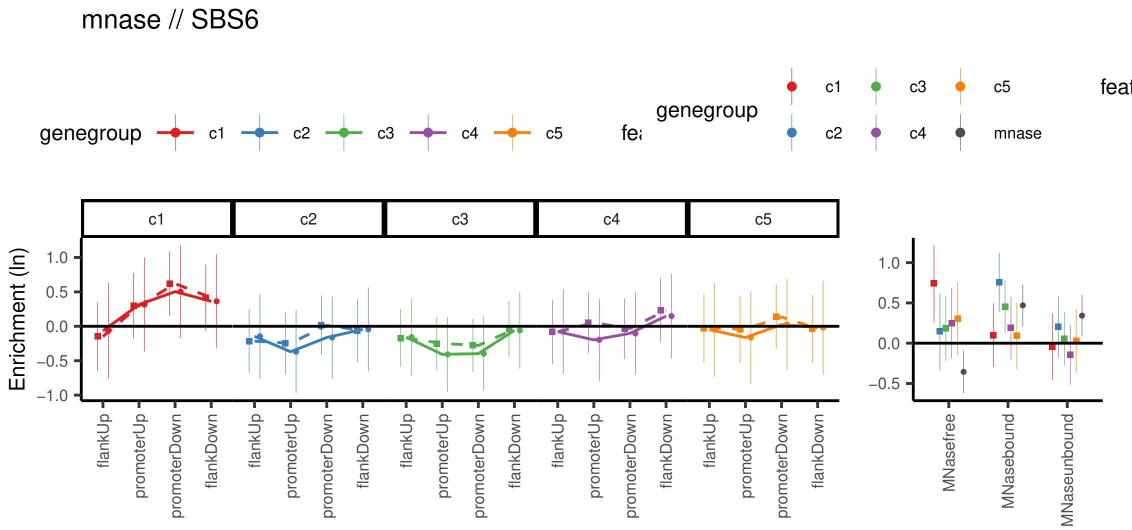

mnase // SBS6
genegroup
c1
c2
c3
c4
c5
fea
genegroup
c1
c3
c5
c2
c4
mnase
feat
Enrichment (ln)
1.0
0.5
0.0
−0.5
−1.0
flankUp
promoterUp
promoterDown
flankDown
MNasefree
MNasebound
MNaseunbound

mnase // SBS7a

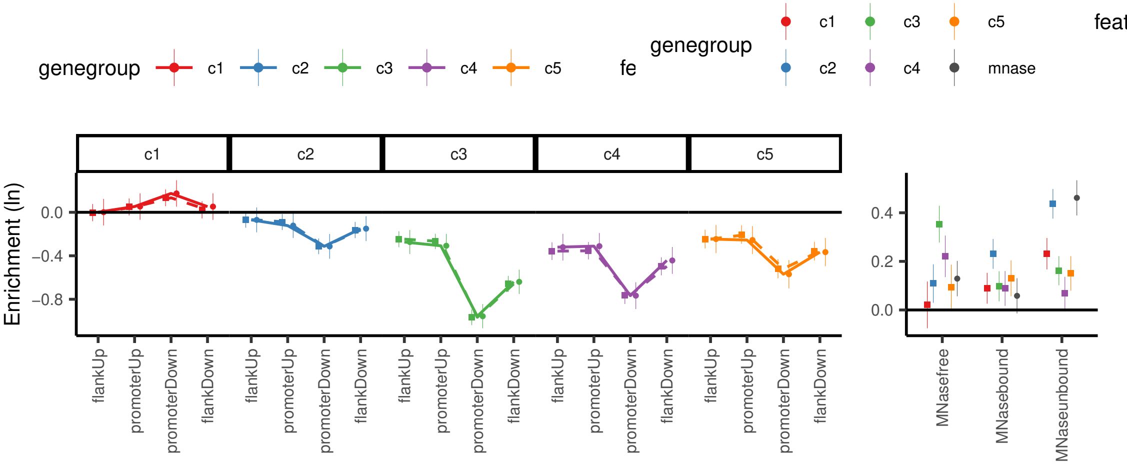
genegroup
c1
c2
c3
c4
c5
fe
genegroup
c1
c2
c3
c4
c5
mnase
feat
c1
c2
c3
c4
c5
Enrichment (ln)
0.0
−0.4
−0.8
flankUp
promoterUp
promoterDown
flankDown
0.4
0.2
0.0
MNasefree
MNasebound
MNaseunbound

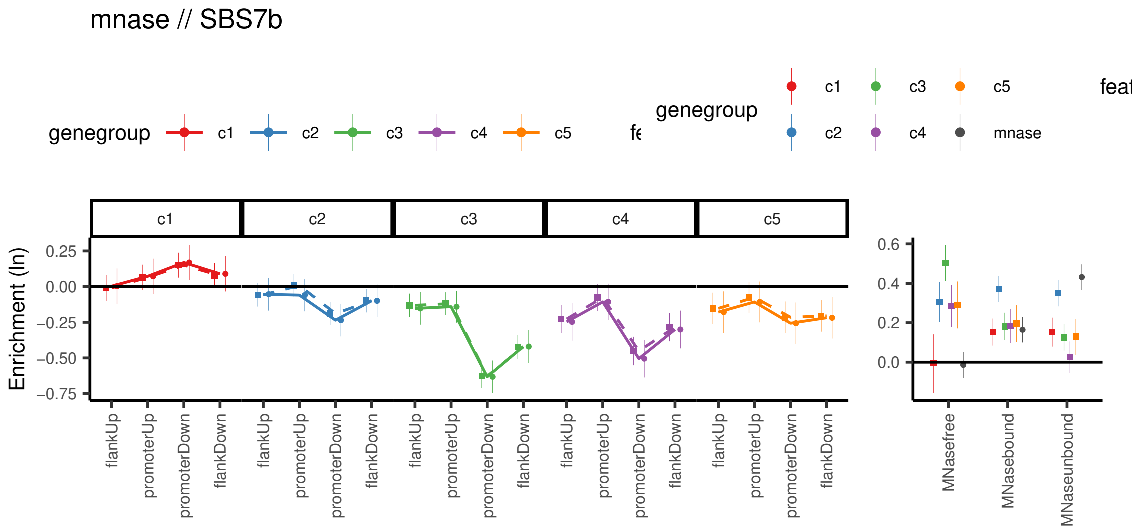

mnase // SBS7b
genegroup
c1
c2
c3
c4
c5
fe
genegroup
c1
c3
c5
c2
c4
mnase
feat
Enrichment (ln)
0.25
0.00
−0.25
−0.50
−0.75
flankUp
promoterUp
promoterDown
flankDown
0.6
0.4
0.2
0.0
MNasefree
MNasebound
MNaseunbound

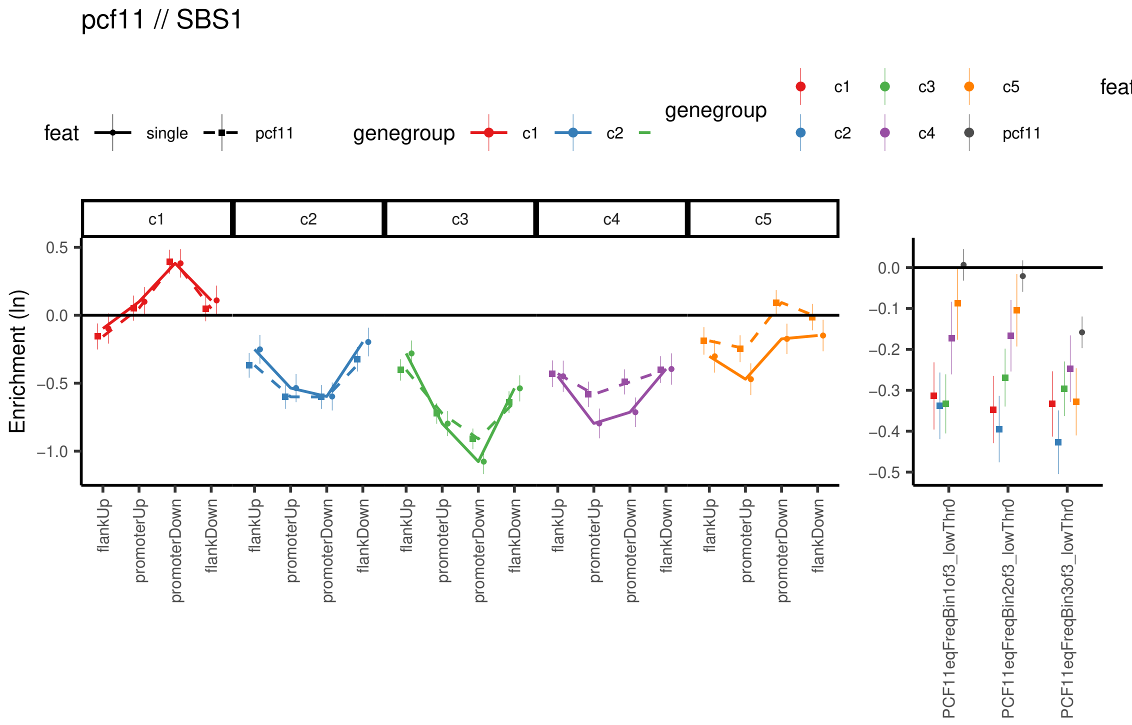
pcf11 // SBS1
feat
single
pcf11
genegroup
c1
c2
genegroup
c1
c3
c5
c2
c4
pcf11
feat
c1
c2
c3
c4
c5
Enrichment (ln)
0.5
0.0
−0.5
−1.0
flankUp
promoterUp
promoterDown
flankDown
0.0
−0.1
−0.2
−0.3
−0.4
−0.5
PCF11eqFreqBin1of3_lowThr0
PCF11eqFreqBin2of3_lowThr0
PCF11eqFreqBin3of3_lowThr0

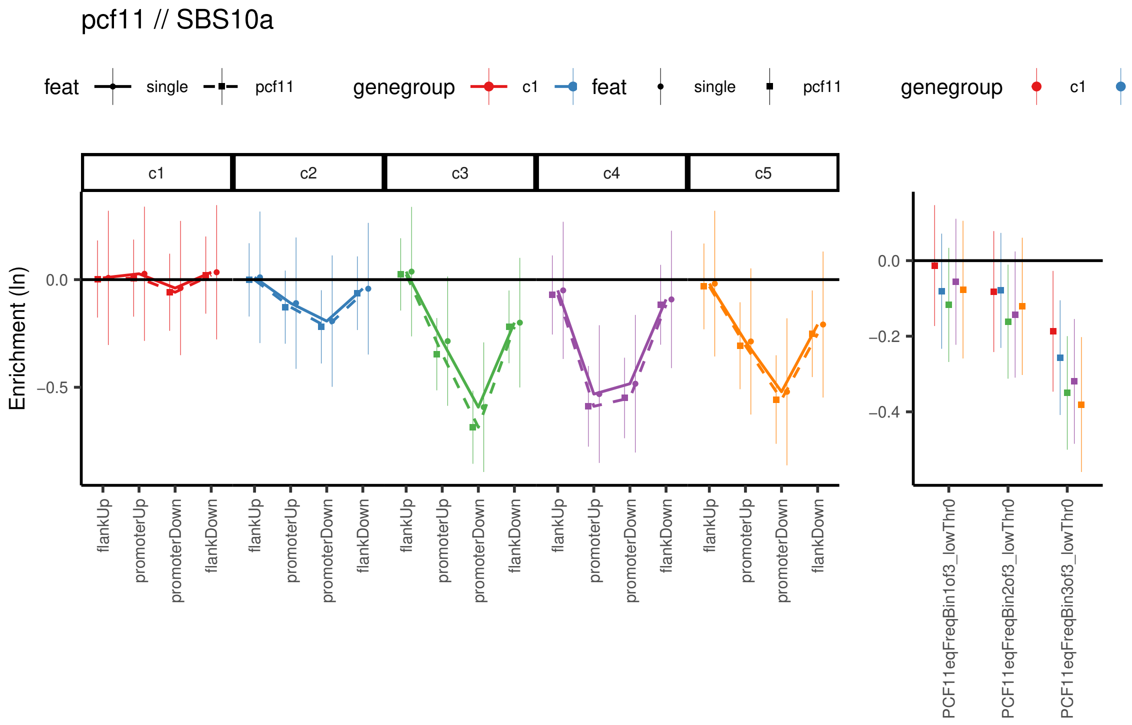
pcf11 // SBS10a
feat
single
pcf11
genegroup
c1
feat
single
pcf11
genegroup
c1
c1
c2
c3
c4
c5
Enrichment (ln)
0.0
−0.5
0.0
−0.2
−0.4
flankUp
promoterUp
promoterDown
flankDown
PCF11eqFreqBin1of3_lowThr0
PCF11eqFreqBin2of3_lowThr0
PCF11eqFreqBin3of3_lowThr0

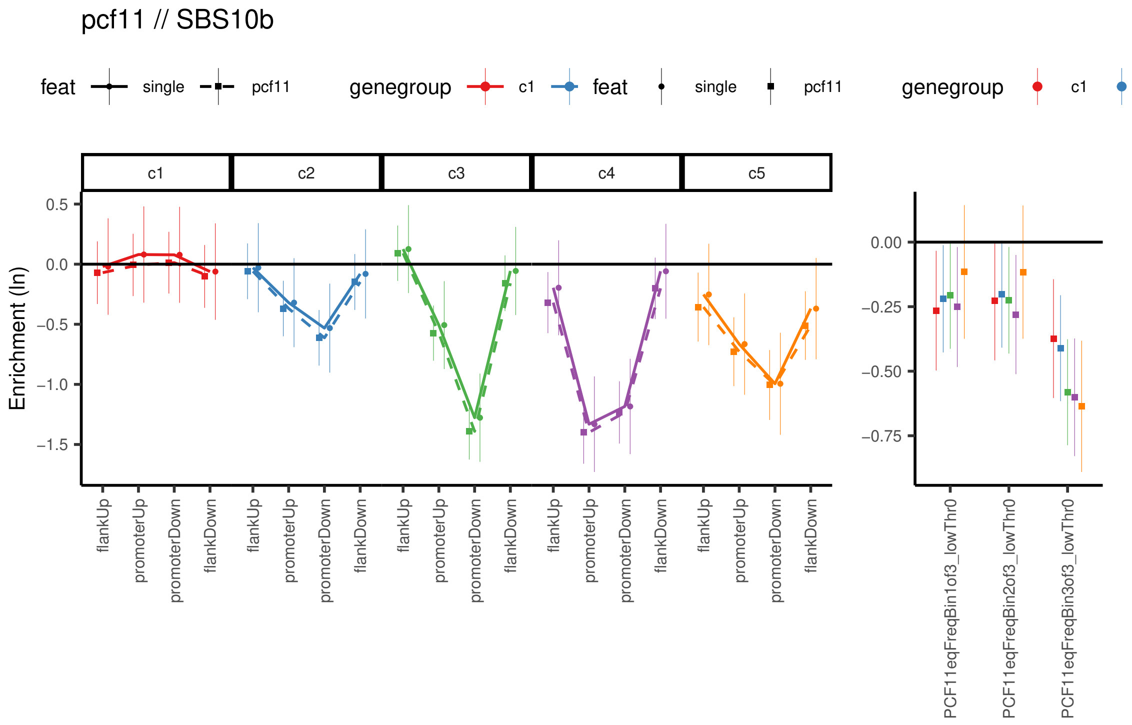

pcf11 // SBS10b
feat
single
pcf11
genegroup
c1
feat
single
pcf11
genegroup
c1
c1
c2
c3
c4
c5
Enrichment (ln)
0.5
0.0
−0.5
−1.0
−1.5
flankUp
promoterUp
promoterDown
flankDown
0.00
−0.25
−0.50
−0.75
PCF11eqFreqBin1of3_lowThr0
PCF11eqFreqBin2of3_lowThr0
PCF11eqFreqBin3of3_lowThr0

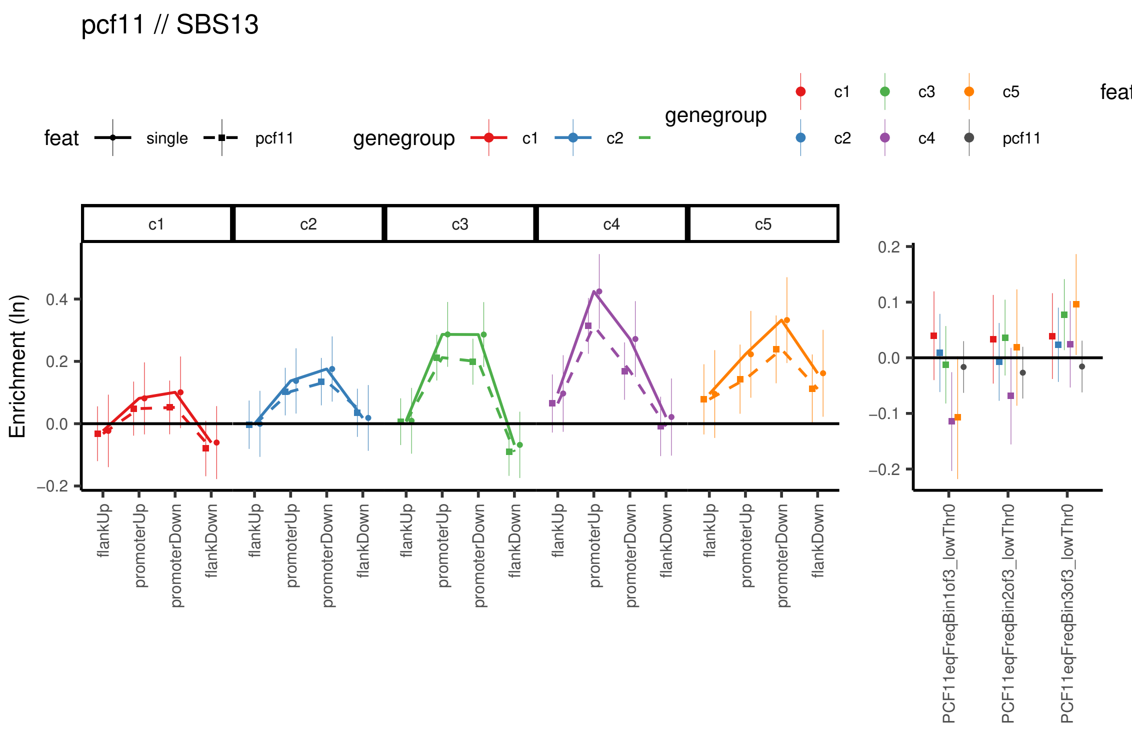
pcf11 // SBS13
feat
single
pcf11
genegroup
c1
c2
genegroup
c1
c3
c5
c2
c4
pcf11
feat
c1
c2
c3
c4
c5
Enrichment (ln)
0.4
0.2
0.0
−0.2
flankUp
promoterUp
promoterDown
flankDown
0.2
0.1
0.0
−0.1
−0.2
PCF11eqFreqBin1of3_lowThr0
PCF11eqFreqBin2of3_lowThr0
PCF11eqFreqBin3of3_lowThr0

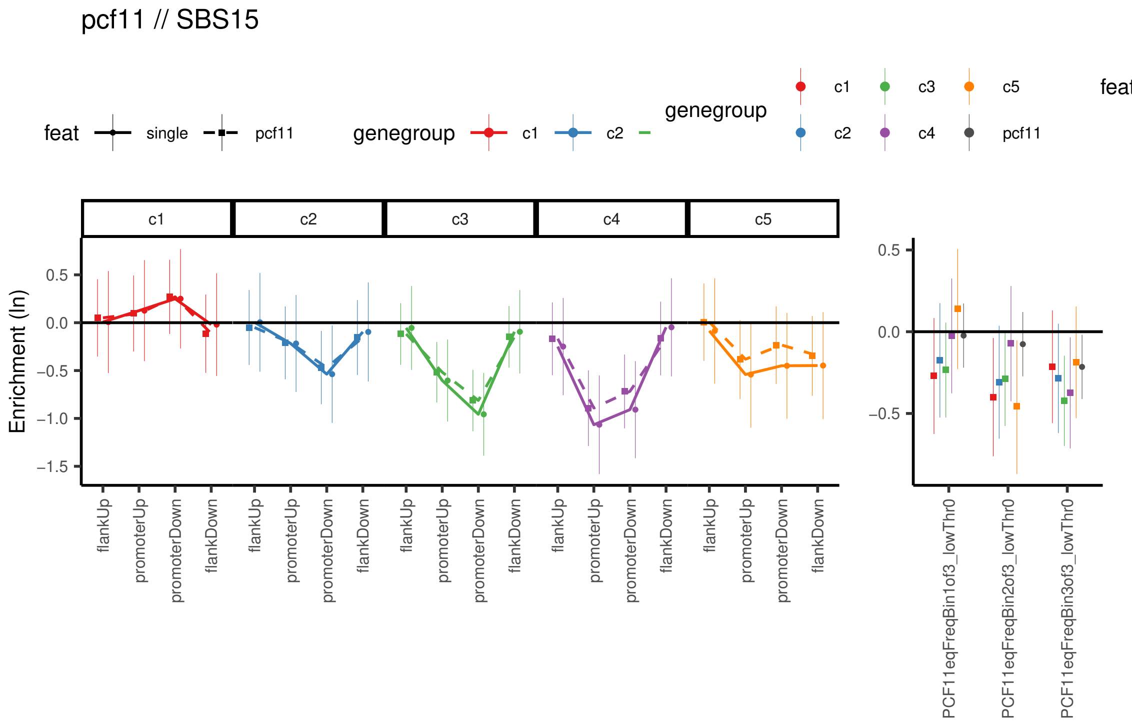
pcf11 // SBS15
feat
single
pcf11
genegroup
c1
c2
genegroup
c1
c2
c3
c4
c5
pcf11
feat
c1
c2
c3
c4
c5
Enrichment (ln)
0.5
0.0
−0.5
−1.0
−1.5
flankUp
promoterUp
promoterDown
flankDown
0.5
0.0
−0.5
PCF11eqFreqBin1of3_lowThr0
PCF11eqFreqBin2of3_lowThr0
PCF11eqFreqBin3of3_lowThr0

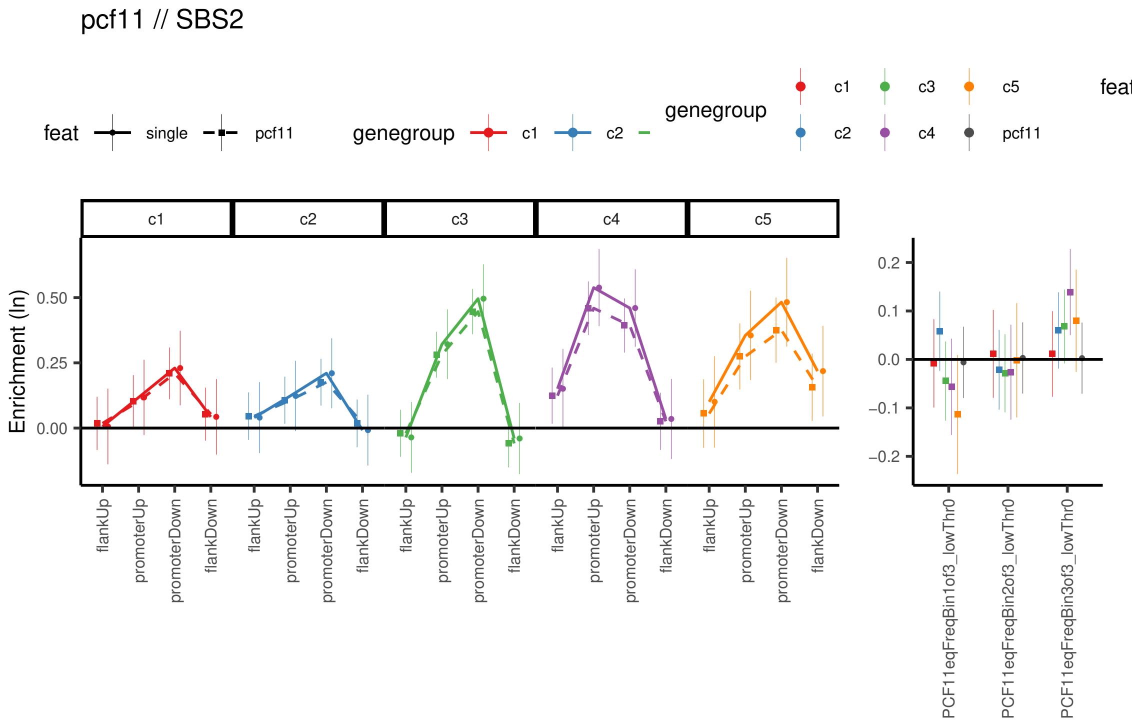
pcf11 // SBS2
feat
single
pcf11
genegroup
c1
c2
genegroup
c1
c2
c3
c4
c5
pcf11
feat
Enrichment (ln)
0.50
0.25
0.00
c1
c2
c3
c4
c5
flankUp
promoterUp
promoterDown
flankDown
0.2
0.1
0.0
−0.1
−0.2
PCF11eqFreqBin1of3_lowThr0
PCF11eqFreqBin2of3_lowThr0
PCF11eqFreqBin3of3_lowThr0

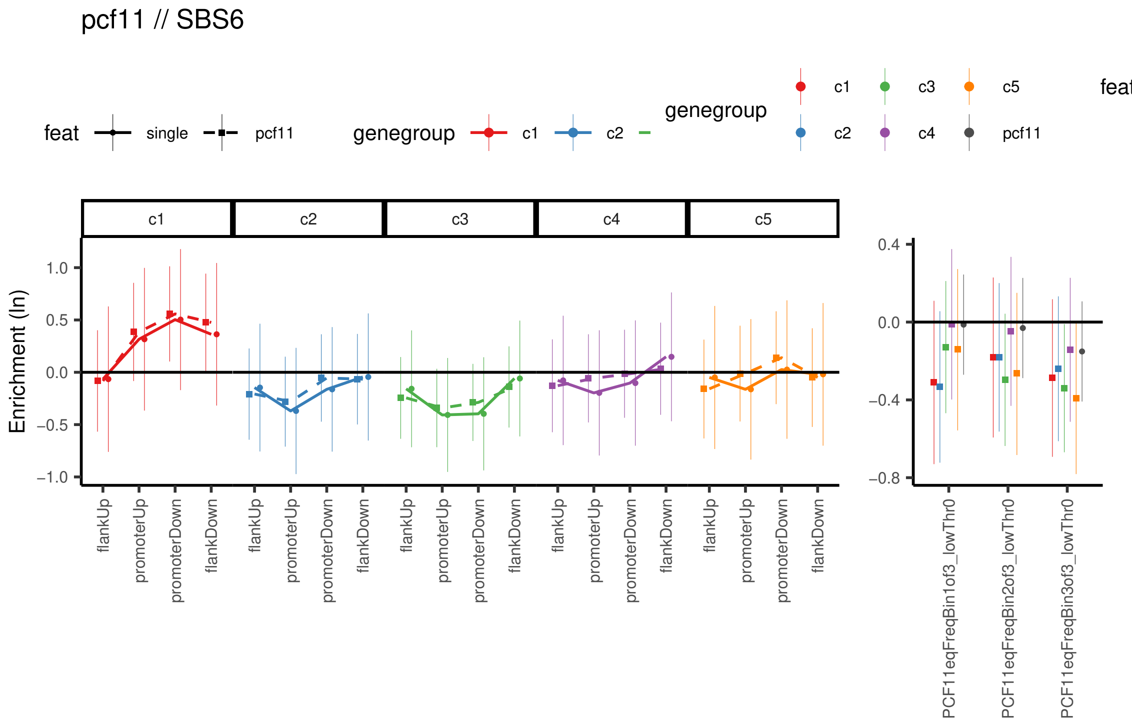

pcf11 // SBS6
feat
single
pcf11
genegroup
c1
c2
genegroup
c1
c3
c5
c2
c4
pcf11
feat
c1
c2
c3
c4
c5
Enrichment (ln)
1.0
0.5
0.0
−0.5
−1.0
flankUp
promoterUp
promoterDown
flankDown
0.4
0.0
−0.4
−0.8
PCF11eqFreqBin1of3_lowThr0
PCF11eqFreqBin2of3_lowThr0
PCF11eqFreqBin3of3_lowThr0

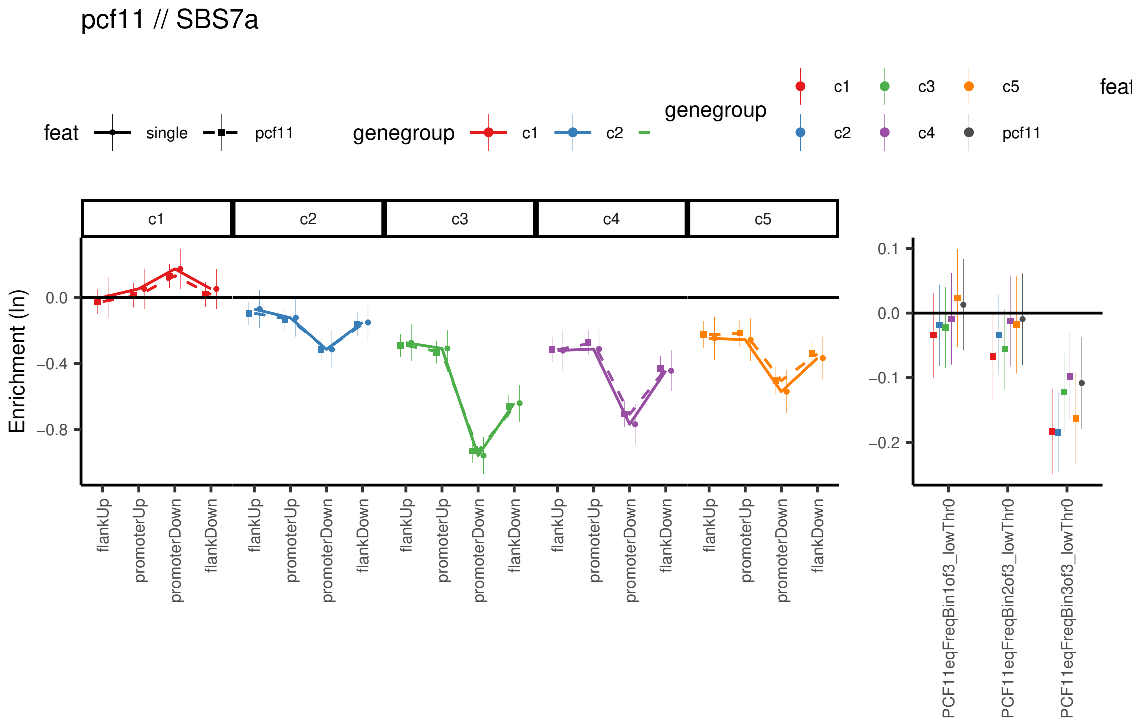
pcf11 // SBS7a
feat
single
pcf11
genegroup
c1
c2
genegroup
c1
c3
c5
c2
c4
pcf11
feat
c1
c2
c3
c4
c5
Enrichment (ln)
0.0
−0.4
−0.8
flankUp
promoterUp
promoterDown
flankDown
0.1
0.0
−0.1
−0.2
PCF11eqFreqBin1of3_lowThr0
PCF11eqFreqBin2of3_lowThr0
PCF11eqFreqBin3of3_lowThr0

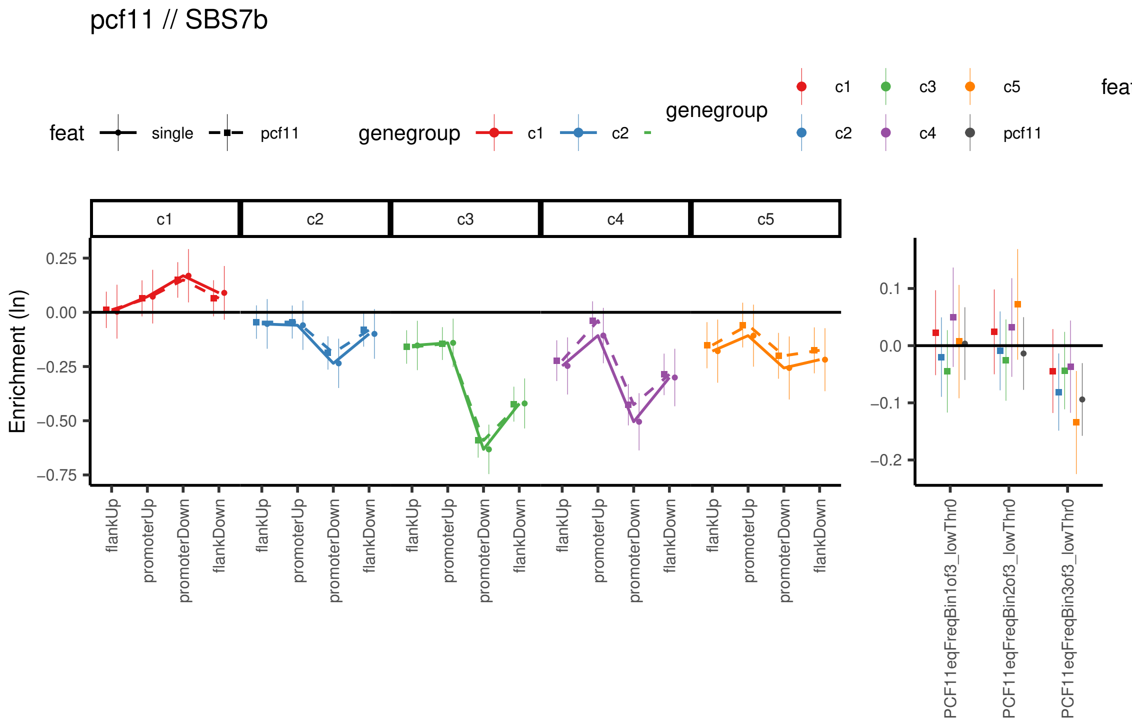

pcf11 // SBS7b
feat
single
pcf11
genegroup
c1
c2
c3
c4
c5
pcf11
Enrichment (ln)
0.25
0.00
−0.25
−0.50
−0.75
flankUp
promoterUp
promoterDown
flankDown
0.1
0.0
−0.1
−0.2
PCF11eqFreqBin1of3_lowThr0
PCF11eqFreqBin2of3_lowThr0
PCF11eqFreqBin3of3_lowThr0

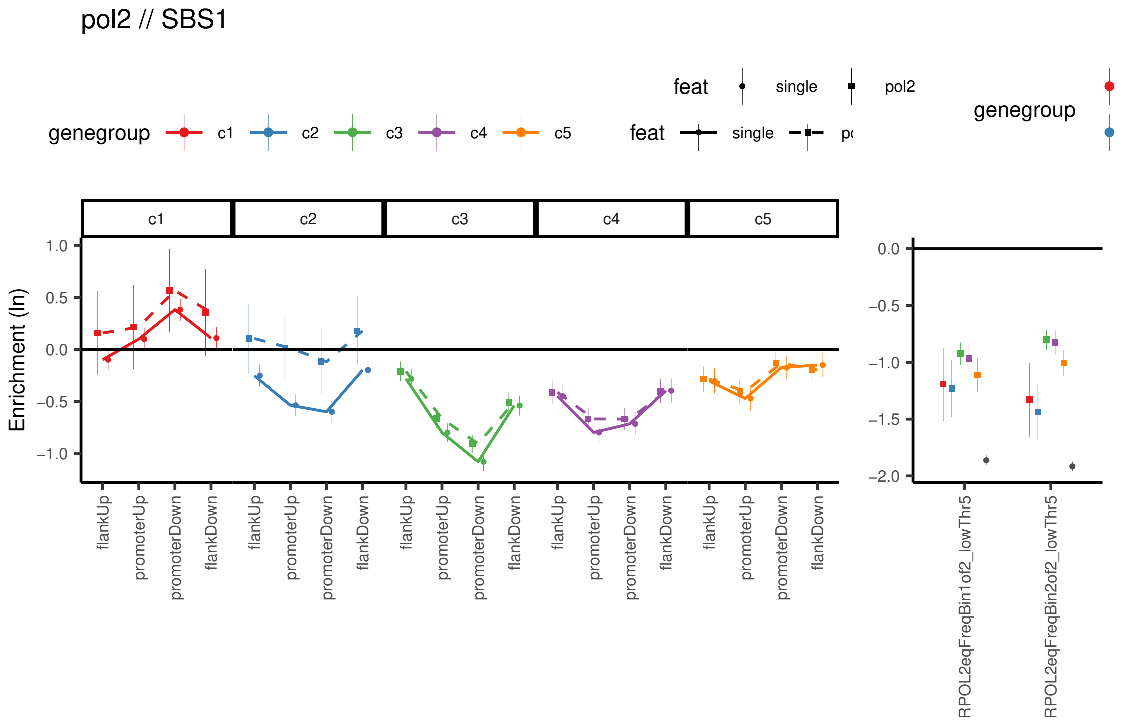

pol2 // SBS1
feat
single
pol2
genegroup
c1
c2
c3
c4
c5
feat
single
genegroup
Enrichment (ln)
1.0
0.5
0.0
−0.5
−1.0
flankUp
promoterUp
promoterDown
flankDown
0.0
−0.5
−1.0
−1.5
−2.0
RPOL2eqFreqBin1of2_lowThr5
RPOL2eqFreqBin2of2_lowThr5

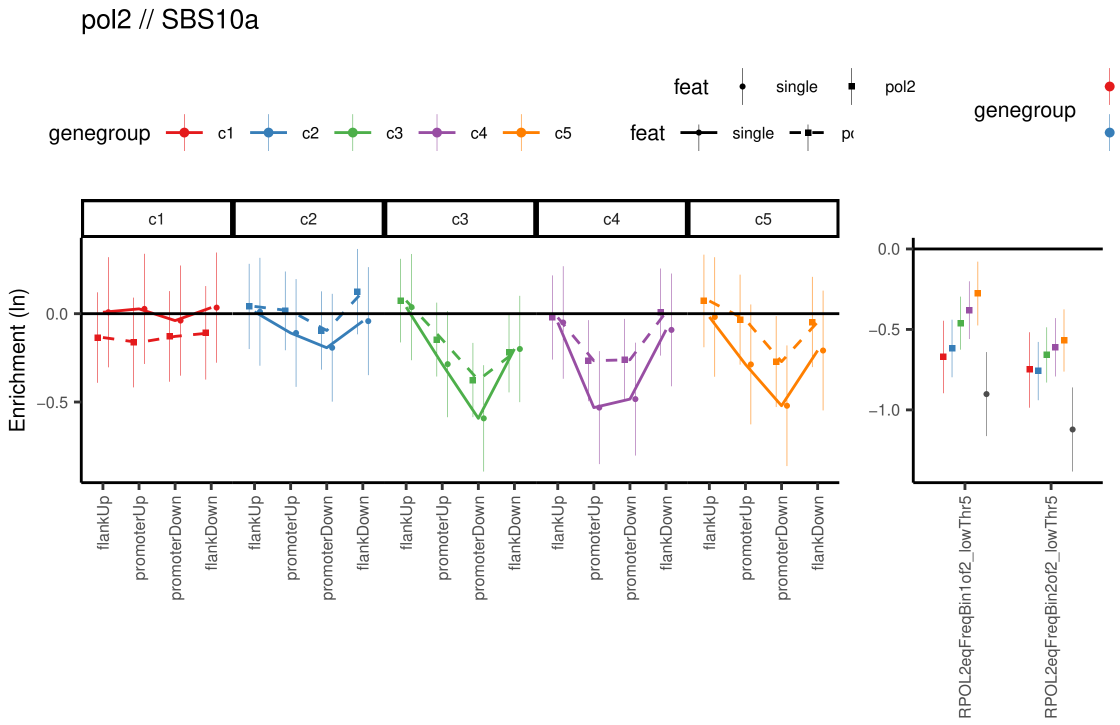

pol2 // SBS10a
feat
single
pol2
genegroup
genegroup
c1
c2
c3
c4
c5
feat
single
c1
c2
c3
c4
c5
Enrichment (ln)
0.0
−0.5
flankUp
promoterUp
promoterDown
flankDown
0.0
−0.5
−1.0
RPOL2eqFreqBin1of2_lowThr5
RPOL2eqFreqBin2of2_lowThr5

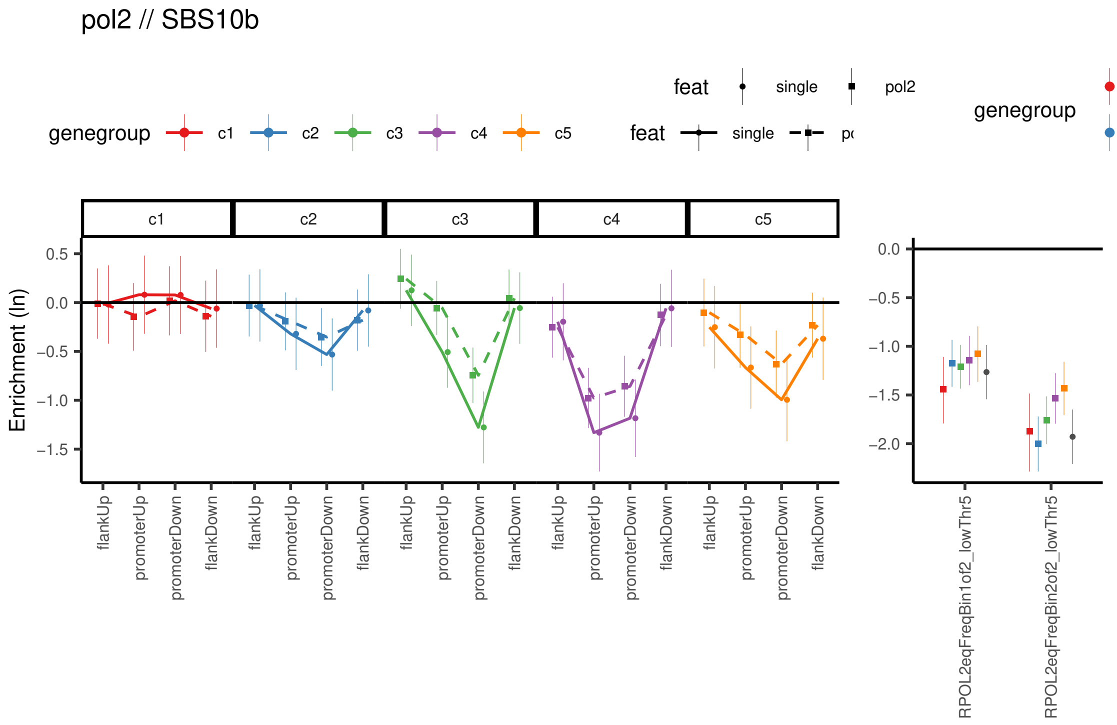

pol2 // SBS10b
feat
single
pol2
genegroup
c1
c2
c3
c4
c5
Enrichment (ln)
0.5
0.0
−0.5
−1.0
−1.5
−2.0
flankUp
promoterUp
promoterDown
flankDown
RPOL2eqFreqBin1of2_lowThr5
RPOL2eqFreqBin2of2_lowThr5

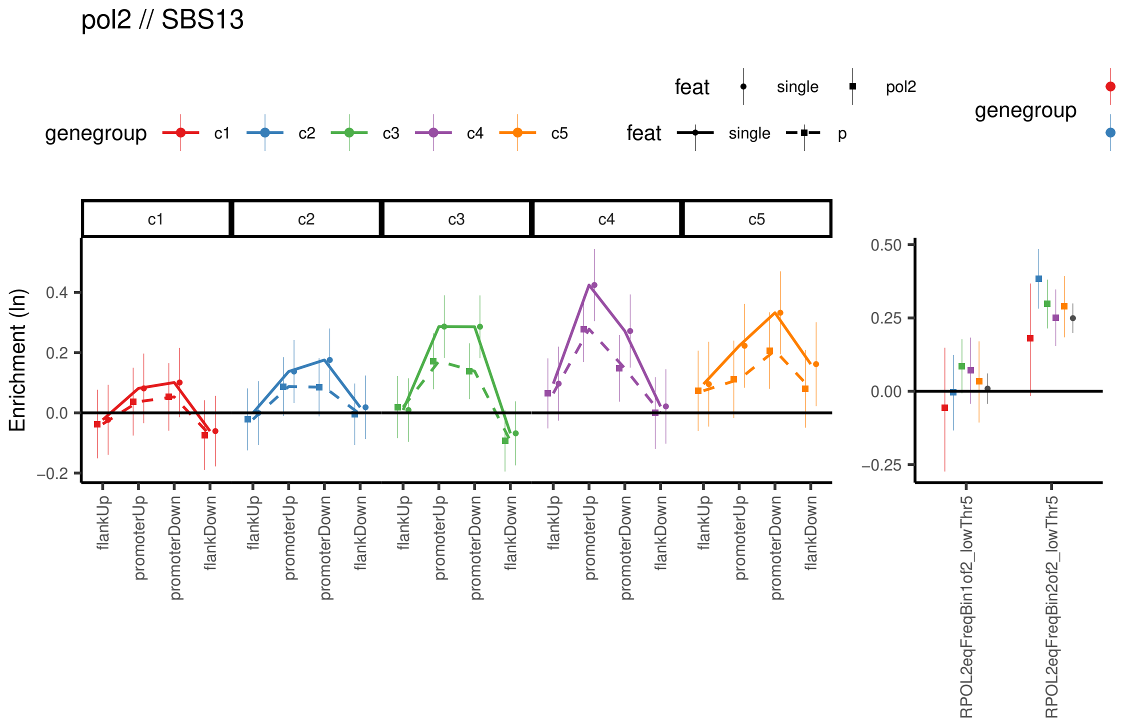
pol2 // SBS13
feat
single
pol2
genegroup
c1
c2
c3
c4
c5
feat
single
p
genegroup
c1
c2
c3
c4
c5
Enrichment (ln)
0.4
0.2
0.0
−0.2
flankUp
promoterUp
promoterDown
flankDown
0.50
0.25
0.00
−0.25
RPOL2eqFreqBin1of2_lowThr5
RPOL2eqFreqBin2of2_lowThr5

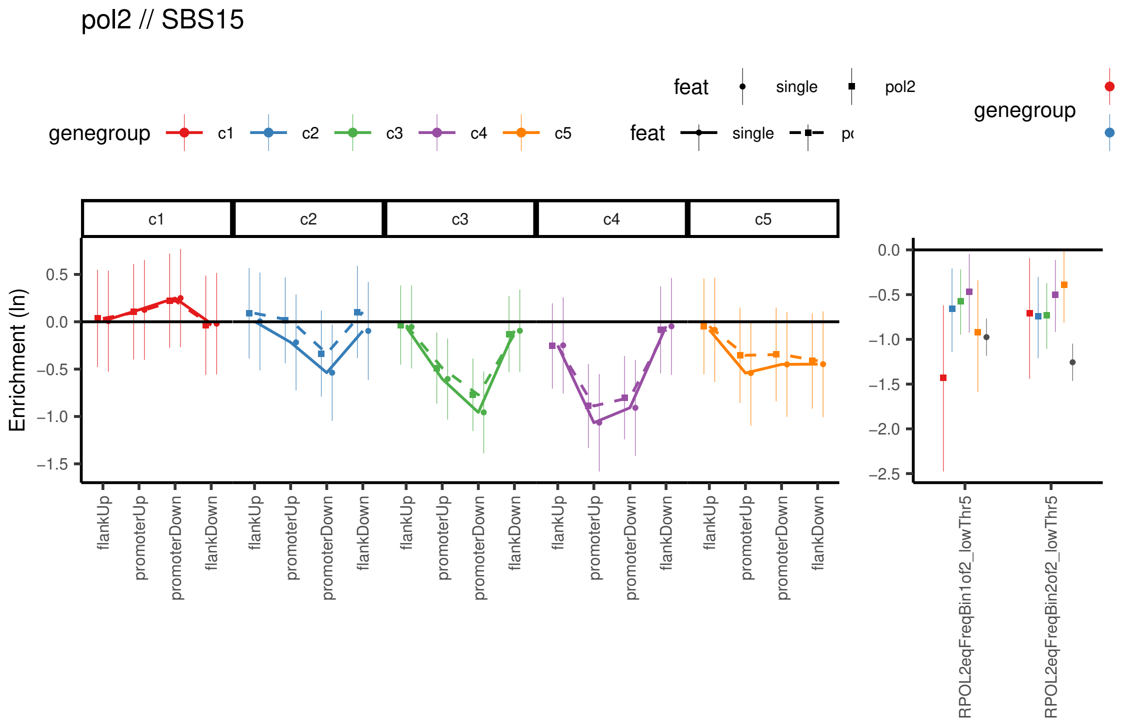

pol2 // SBS15
feat
single
pol2
genegroup
c1
c2
c3
c4
c5
Enrichment (ln)
0.5
0.0
−0.5
−1.0
−1.5
−2.0
−2.5
flankUp
promoterUp
promoterDown
flankDown
RPOL2eqFreqBin1of2_lowThr5
RPOL2eqFreqBin2of2_lowThr5

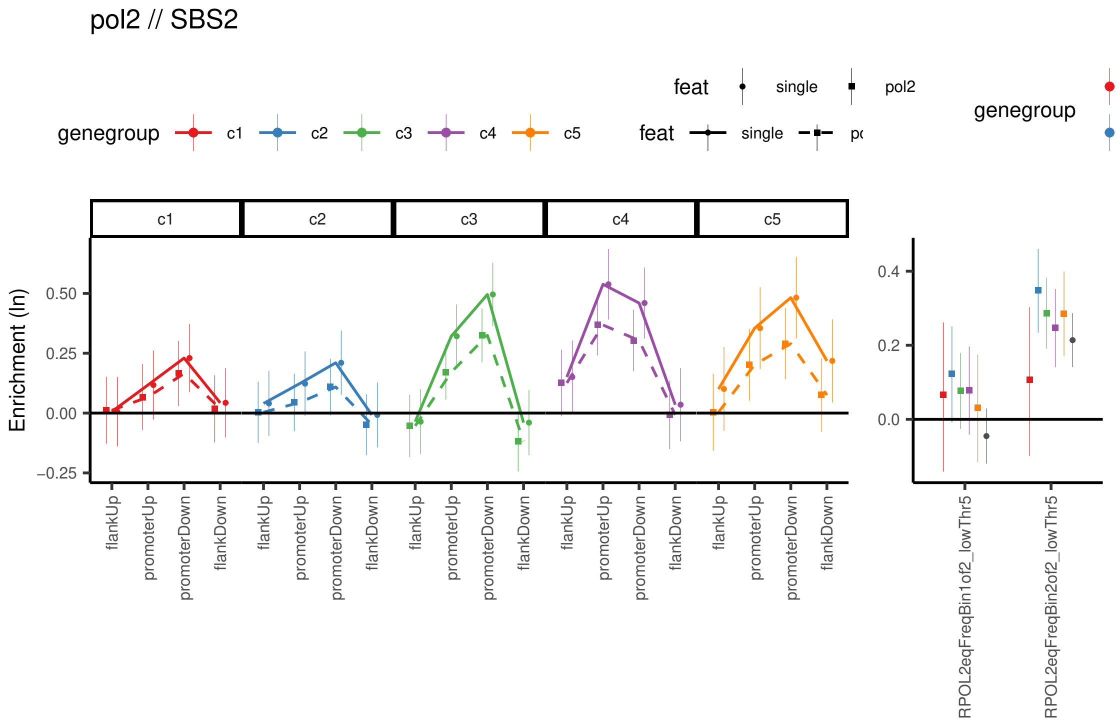

pol2 // SBS2
feat
single
pol2
genegroup
c1
c2
c3
c4
c5
feat
single
po
genegroup
c1
c2
c3
c4
c5
Enrichment (ln)
0.50
0.25
0.00
−0.25
flankUp
promoterUp
promoterDown
flankDown
0.4
0.2
0.0
RPOL2eqFreqBin1of2_lowThr5
RPOL2eqFreqBin2of2_lowThr5

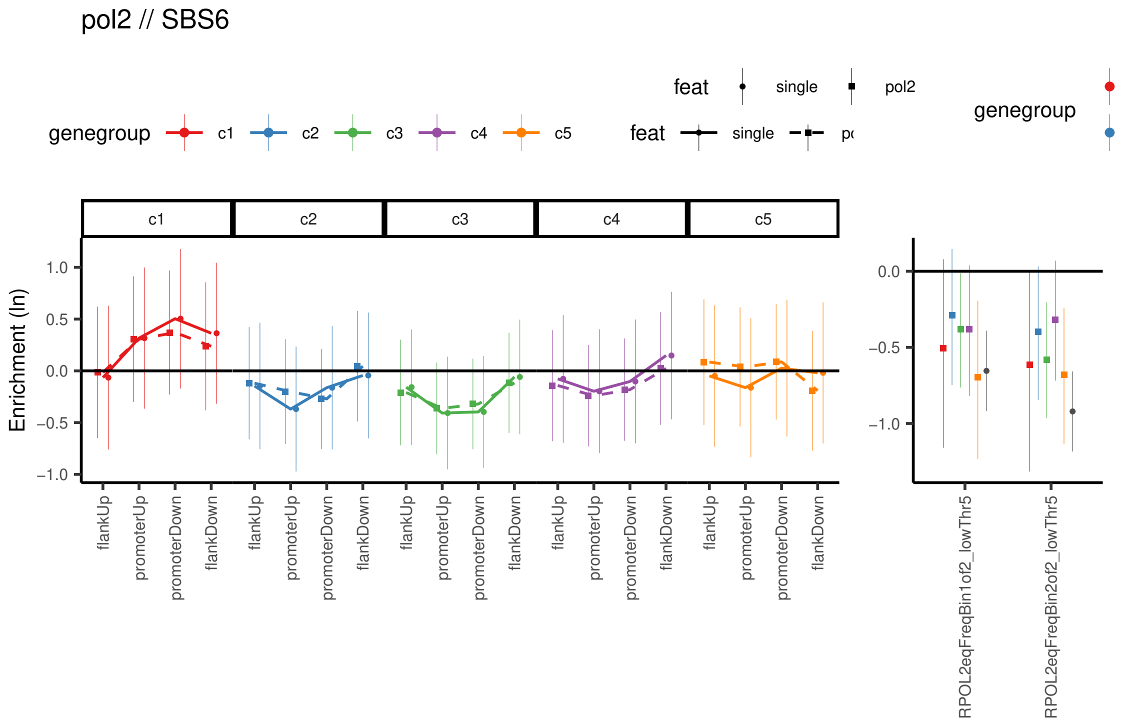

pol2 // SBS6
feat
single
pol2
genegroup
c1
c2
c3
c4
c5
feat
single
genegroup
c1
c2
c3
c4
c5
Enrichment (ln)
1.0
0.5
0.0
−0.5
−1.0
flankUp
promoterUp
promoterDown
flankDown
0.0
−0.5
−1.0
RPOL2eqFreqBin1of2_lowThr5
RPOL2eqFreqBin2of2_lowThr5

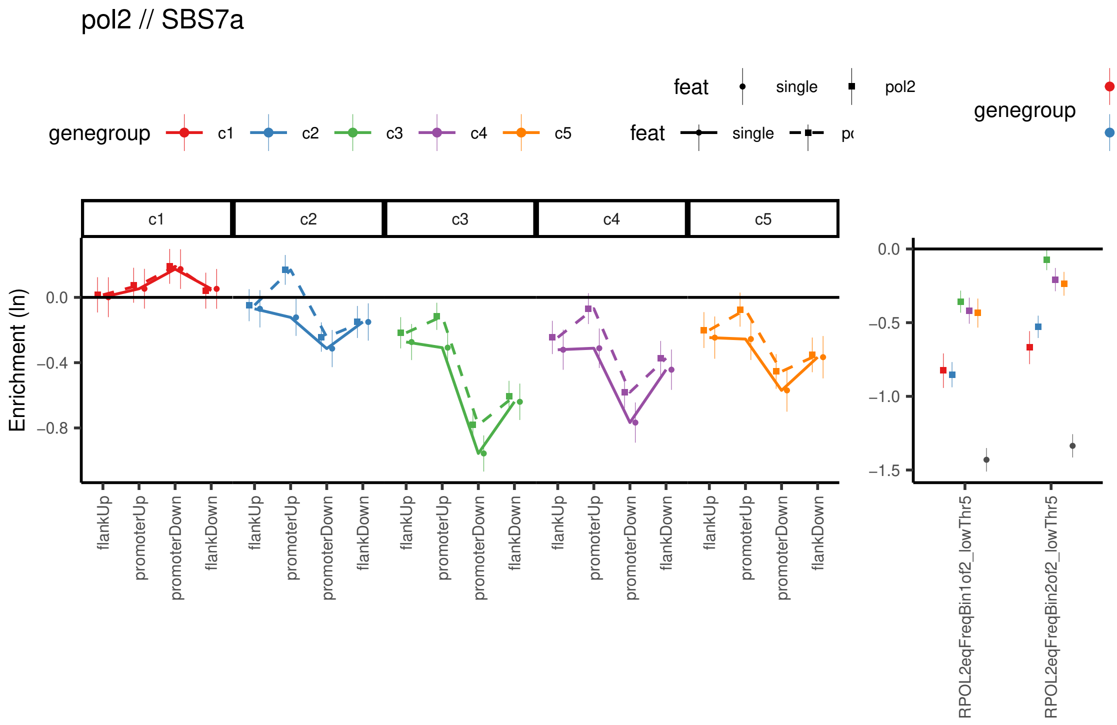

pol2 // SBS7a
feat
single
pol2
genegroup
genegroup
c1
c2
c3
c4
c5
feat
single
pc
c1
c2
c3
c4
c5
Enrichment (ln)
0.0
−0.4
−0.8
flankUp
promoterUp
promoterDown
flankDown
0.0
−0.5
−1.0
−1.5
RPOL2eqFreqBin1of2_lowThr5
RPOL2eqFreqBin2of2_lowThr5

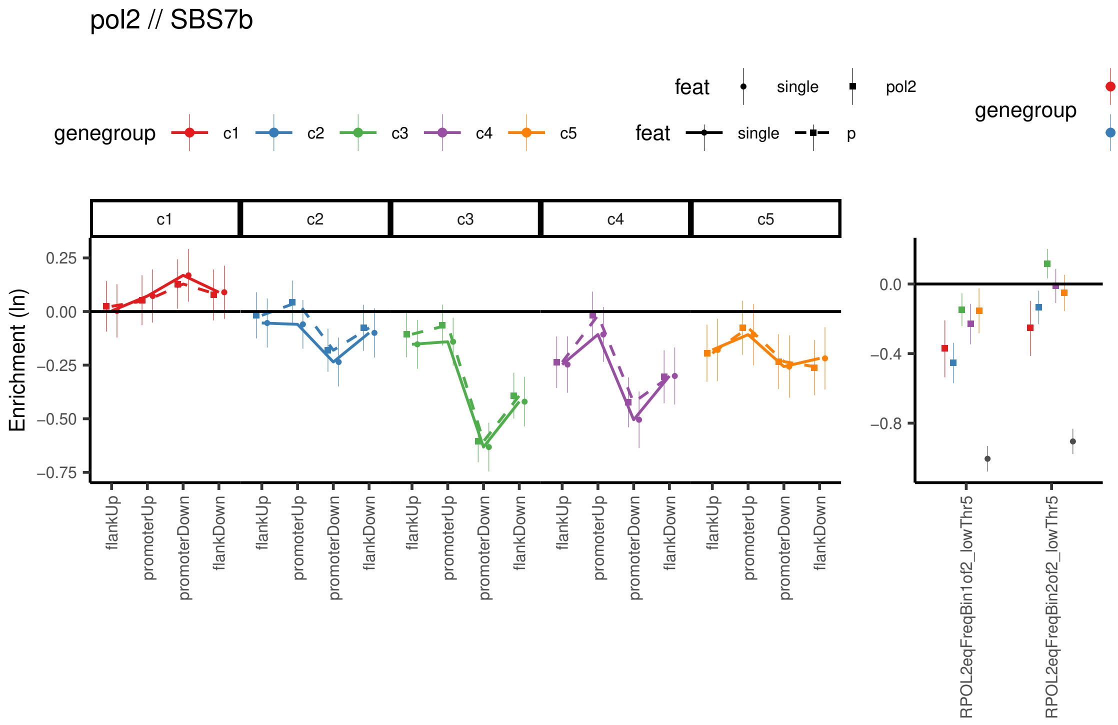
pol2 // SBS7b
feat
single
pol2
genegroup
c1
c2
c3
c4
c5
feat
single
p
genegroup
c1
c2
c3
c4
c5
Enrichment (ln)
0.25
0.00
−0.25
−0.50
−0.75
0.0
−0.4
−0.8
flankUp
promoterUp
promoterDown
flankDown
RPOL2eqFreqBin1of2_lowThr5
RPOL2eqFreqBin2of2_lowThr5

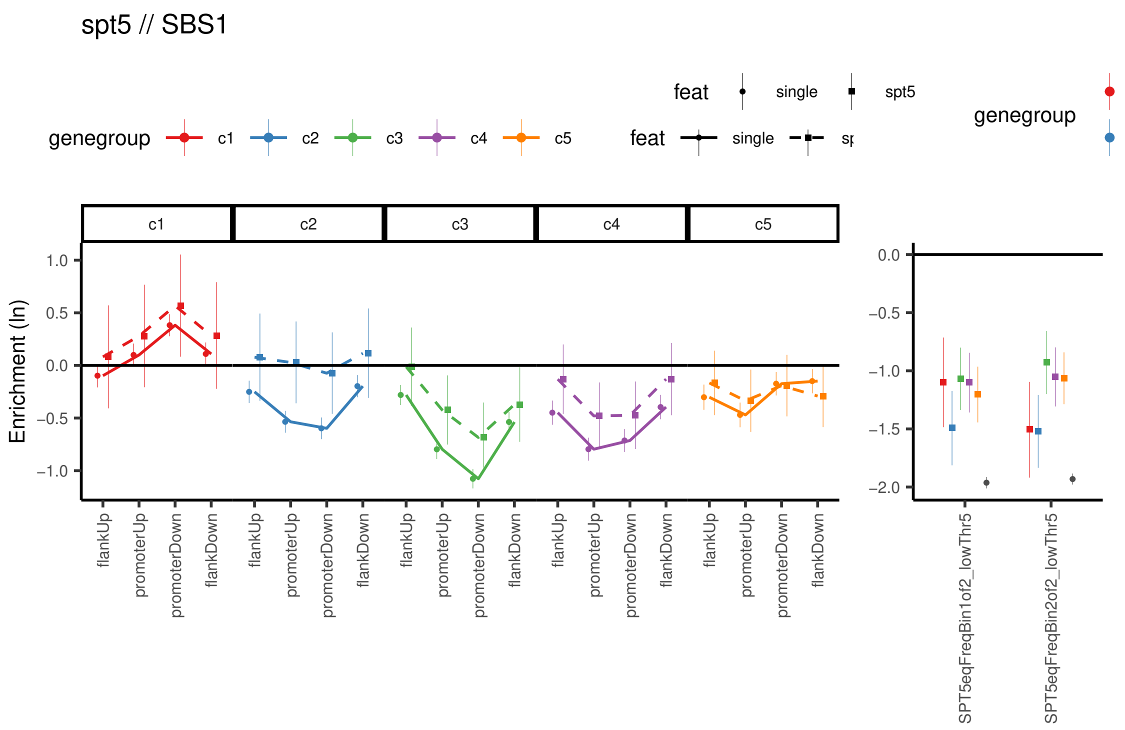
spt5 // SBS1
feat
single
spt5
genegroup
c1
c2
c3
c4
c5
feat
single
sp
genegroup
Enrichment (ln)
1.0
0.5
0.0
−0.5
−1.0
flankUp
promoterUp
promoterDown
flankDown
0.0
−0.5
−1.0
−1.5
−2.0
SPT5eqFreqBin1of2_lowThr5
SPT5eqFreqBin2of2_lowThr5

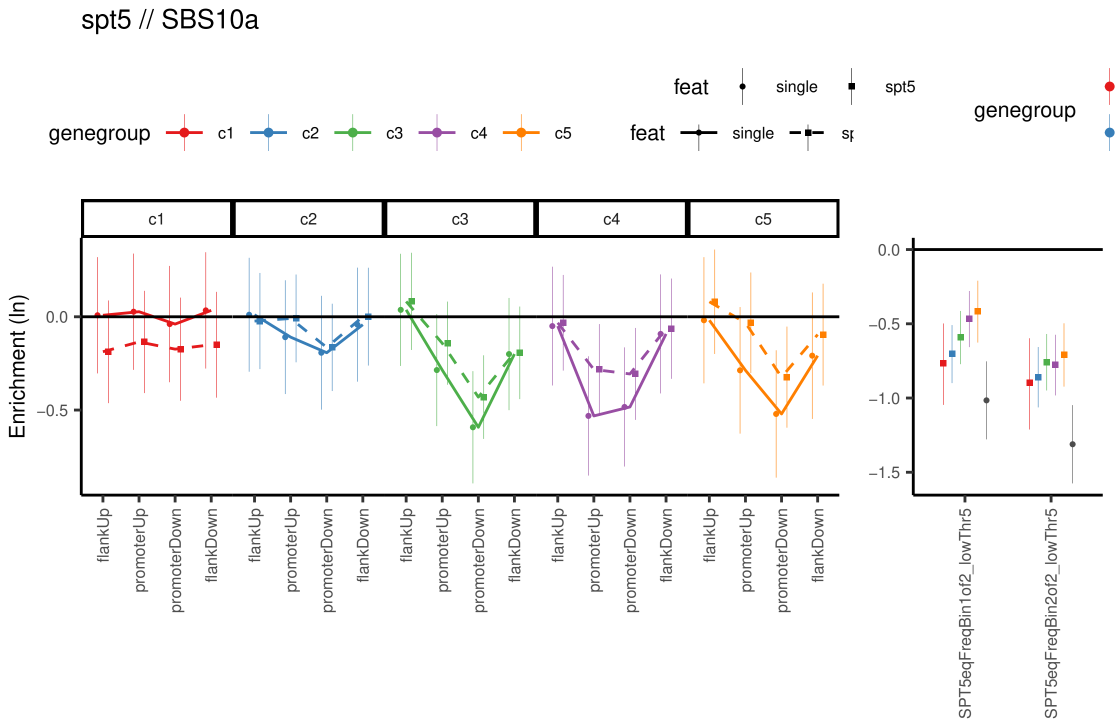
spt5 // SBS10a
feat
single
spt5
genegroup
genegroup
c1
c2
c3
c4
c5
feat
single
Enrichment (ln)
0.0
−0.5
−1.0
−1.5
flankUp
promoterUp
promoterDown
flankDown
SPT5eqFreqBin1of2_lowThr5
SPT5eqFreqBin2of2_lowThr5

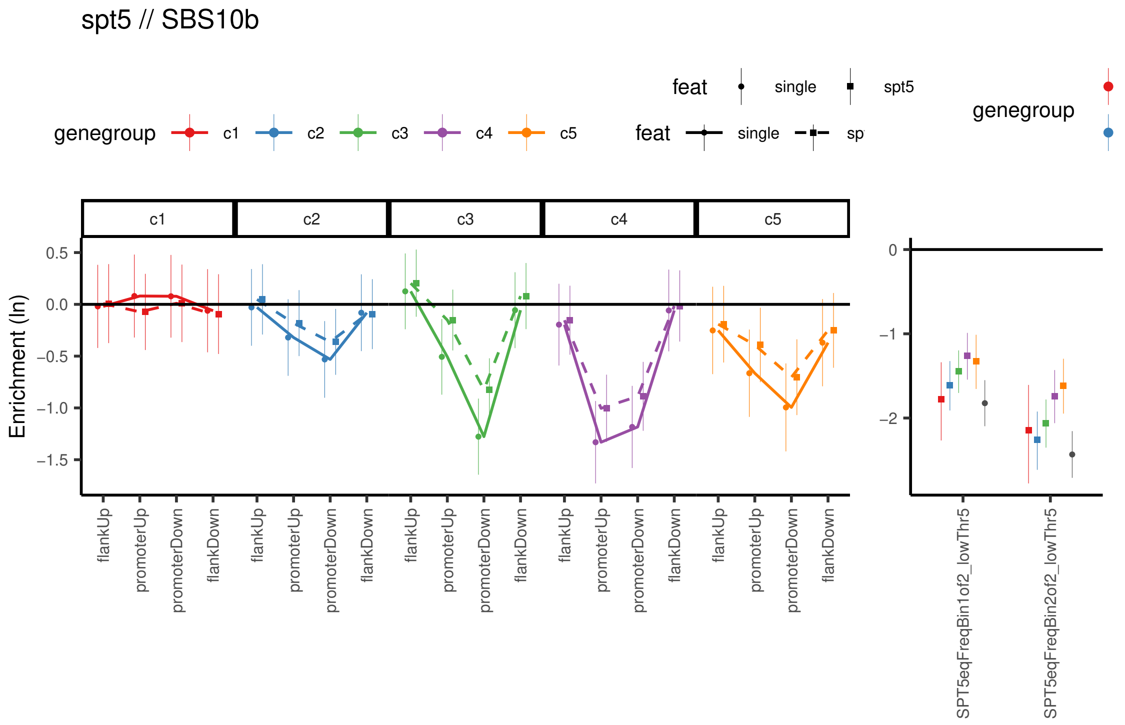

spt5 // SBS10b
feat
single
spt5
genegroup
c1
c2
c3
c4
c5
feat
single
sp
genegroup
Enrichment (ln)
0.5
0.0
−0.5
−1.0
−1.5
0
−1
−2
flankUp
promoterUp
promoterDown
flankDown
SPT5eqFreqBin1of2_lowThr5
SPT5eqFreqBin2of2_lowThr5

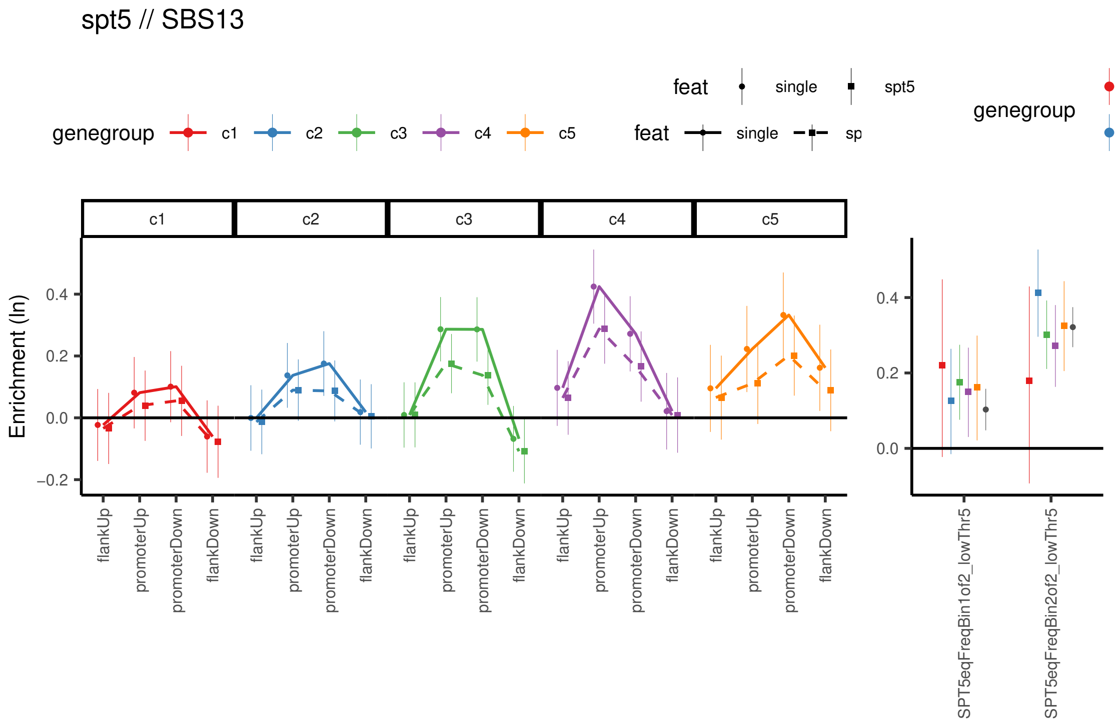
spt5 // SBS13
feat single spt5
genegroup
genegroup c1 c2 c3 c4 c5
feat single sp
c1
c2
c3
c4
c5
Enrichment (ln)
0.4
0.2
0.0
−0.2
flankUp
promoterUp
promoterDown
flankDown
SPT5eqFreqBin1of2_lowThr5
SPT5eqFreqBin2of2_lowThr5

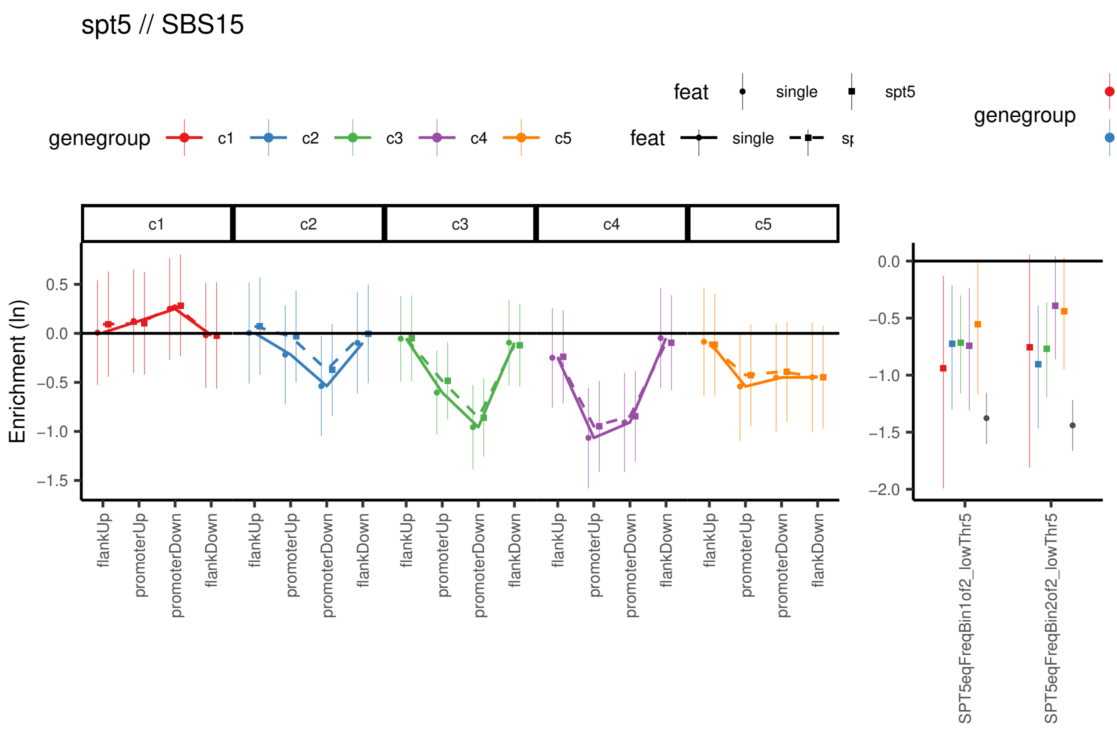

spt5 // SBS15
feat
single
spt5
genegroup
c1
c2
c3
c4
c5
c1
c2
c3
c4
c5
Enrichment (ln)
0.5
0.0
−0.5
−1.0
−1.5
flankUp
promoterUp
promoterDown
flankDown
0.0
−0.5
−1.0
−1.5
−2.0
SPT5eqFreqBin1of2_lowThr5
SPT5eqFreqBin2of2_lowThr5

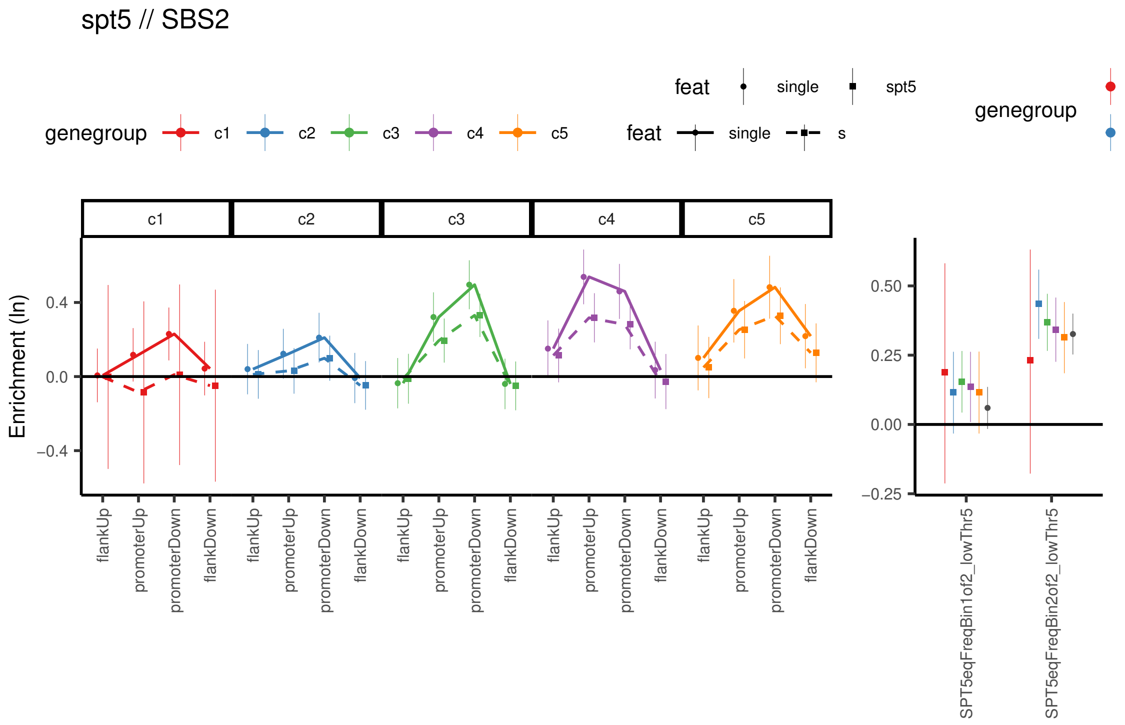

spt5 // SBS2
feat
single
spt5
genegroup
c1
c2
c3
c4
c5
feat
single
s
genegroup
Enrichment (ln)
0.4
0.0
−0.4
flankUp
promoterUp
promoterDown
flankDown
0.50
0.25
0.00
−0.25
SPT5eqFreqBin1of2_lowThr5
SPT5eqFreqBin2of2_lowThr5

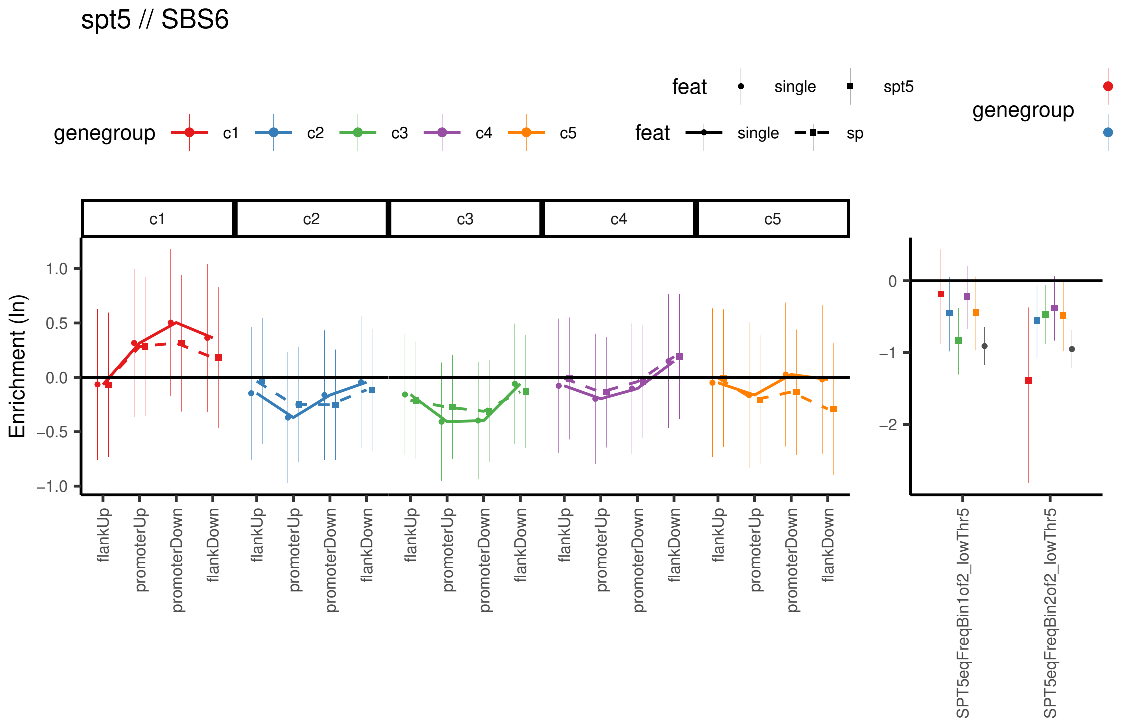

spt5 // SBS6
feat
single
spt5
genegroup
c1
c2
c3
c4
c5
feat
single
sp
genegroup
c1
c2
c3
c4
c5
Enrichment (ln)
1.0
0.5
0.0
−0.5
−1.0
flankUp
promoterUp
promoterDown
flankDown
0
−1
−2
SPT5eqFreqBin1of2_lowThr5
SPT5eqFreqBin2of2_lowThr5

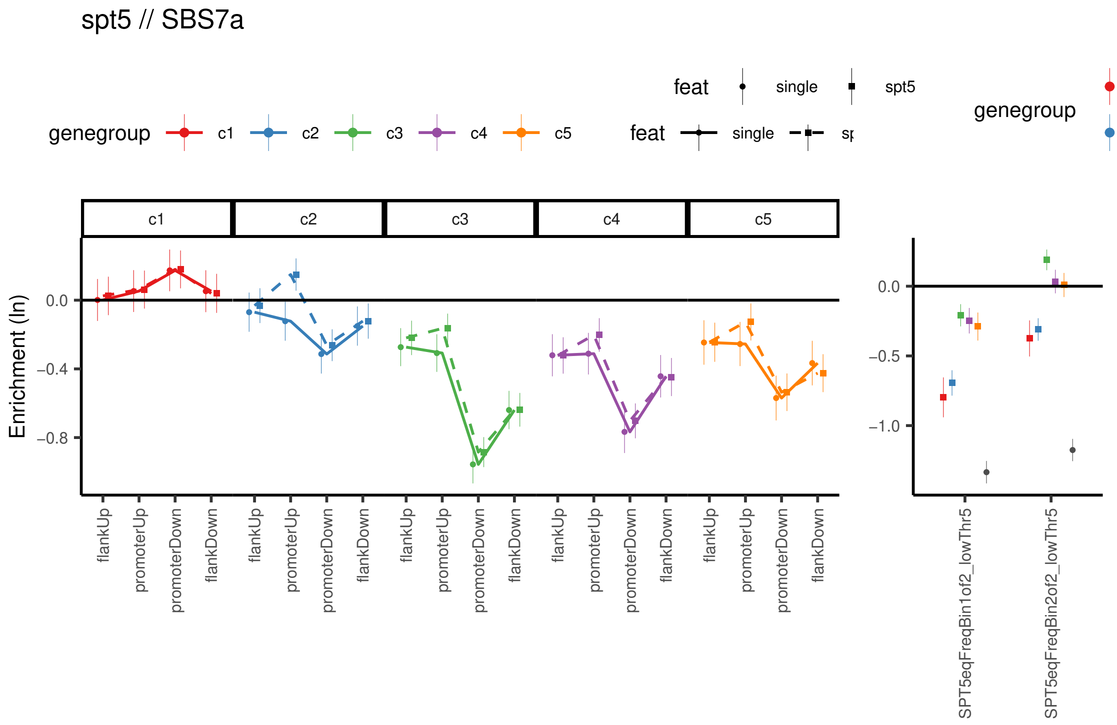
spt5 // SBS7a
feat
single
spt5
genegroup
c1
c2
c3
c4
c5
feat
single
sp
genegroup
Enrichment (ln)
0.0
−0.4
−0.8
0.0
−0.5
−1.0
flankUp
promoterUp
promoterDown
flankDown
SPT5eqFreqBin1of2_lowThr5
SPT5eqFreqBin2of2_lowThr5

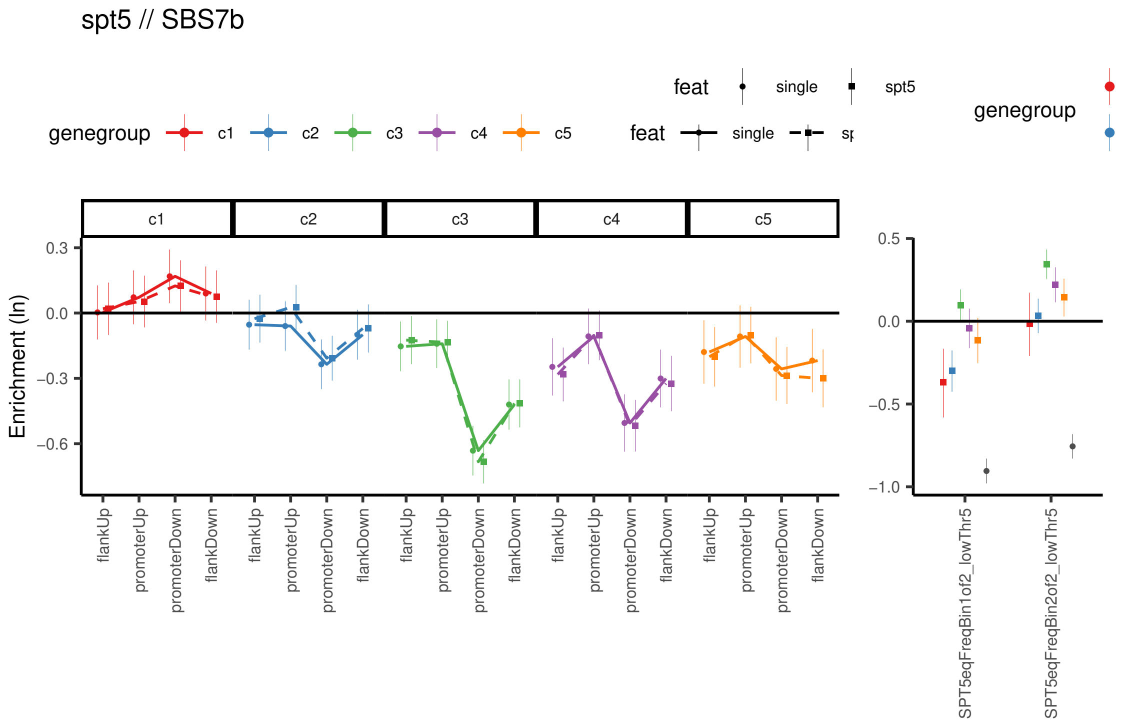

spt5 // SBS7b
feat
single
spt5
genegroup
c1
c2
c3
c4
c5
Enrichment (ln)
0.3
0.0
−0.3
−0.6
0.5
−0.5
−1.0
flankUp
promoterUp
promoterDown
flankDown
SPT5eqFreqBin1of2_lowThr5
SPT5eqFreqBin2of2_lowThr5

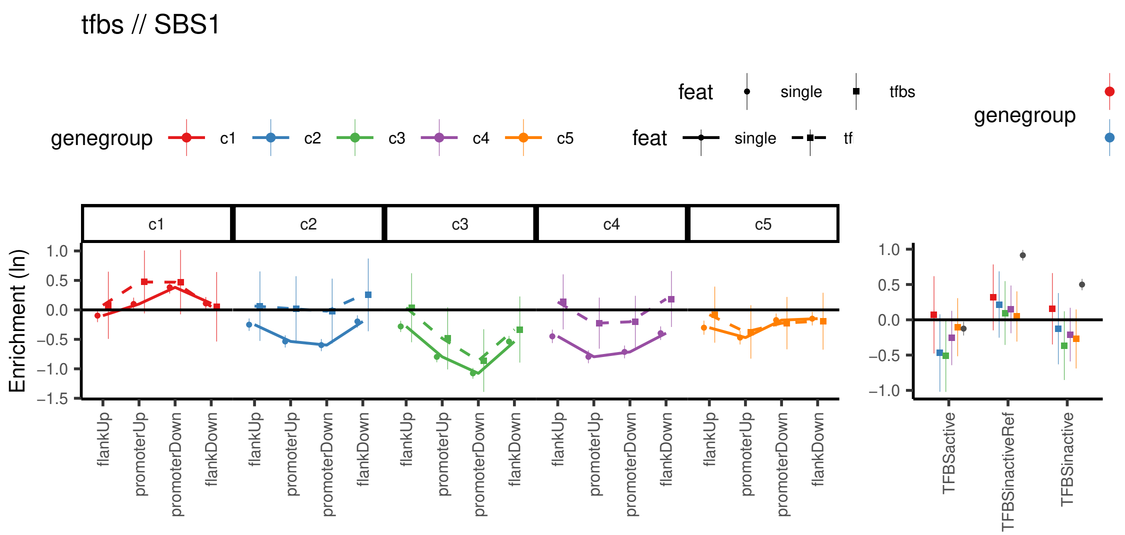

tfbs // SBS1
feat
single
tfbs
genegroup
c1
c2
c3
c4
c5
feat
single
tf
genegroup
c1
c2
c3
c4
c5
Enrichment (ln)
1.0
0.5
0.0
−0.5
−1.0
−1.5
flankUp
promoterUp
promoterDown
flankDown
TFBSactive
TFBSinactiveRef
TFBSinactive

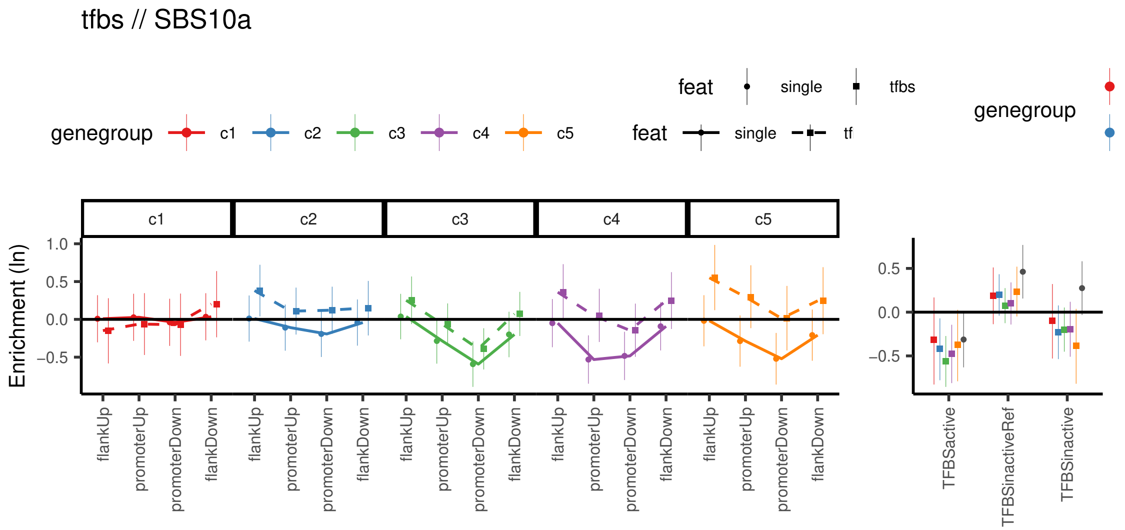

tfbs // SBS10a
feat
single
tfbs
genegroup
genegroup
c1
c2
c3
c4
c5
feat
single
tf
Enrichment (ln)
1.0
0.5
0.0
−0.5
flankUp
promoterUp
promoterDown
flankDown
0.5
0.0
−0.5
TFBSactive
TFBSinactiveRef
TFBSinactive

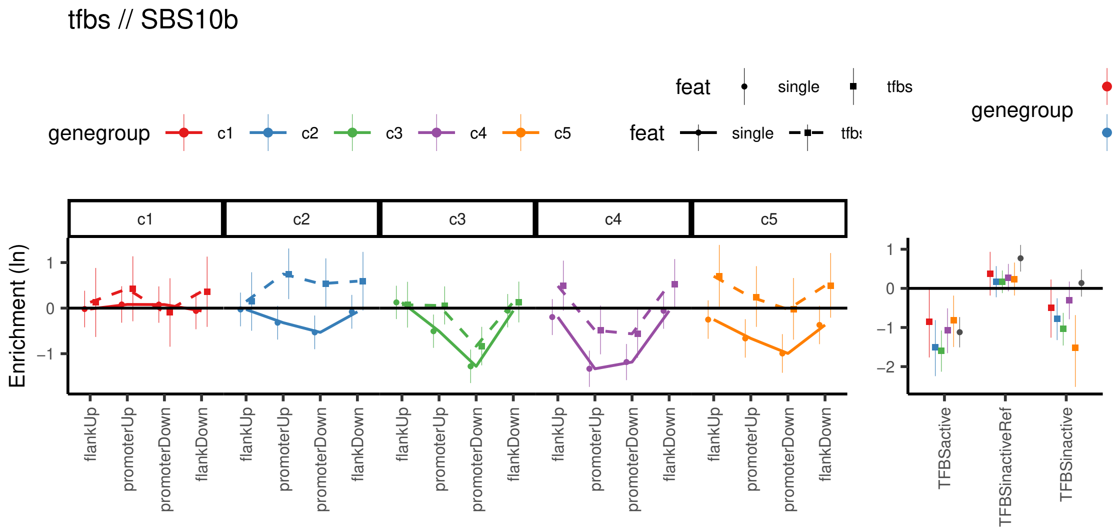
tfbs // SBS10b
feat
single
tfbs
genegroup
c1
c2
c3
c4
c5
feat
single
tfbs
genegroup
c1
c2
c3
c4
c5
Enrichment (ln)
1
0
−1
flankUp
promoterUp
promoterDown
flankDown
1
0
−1
−2
TFBSactive
TFBSinactiveRef
TFBSinactive

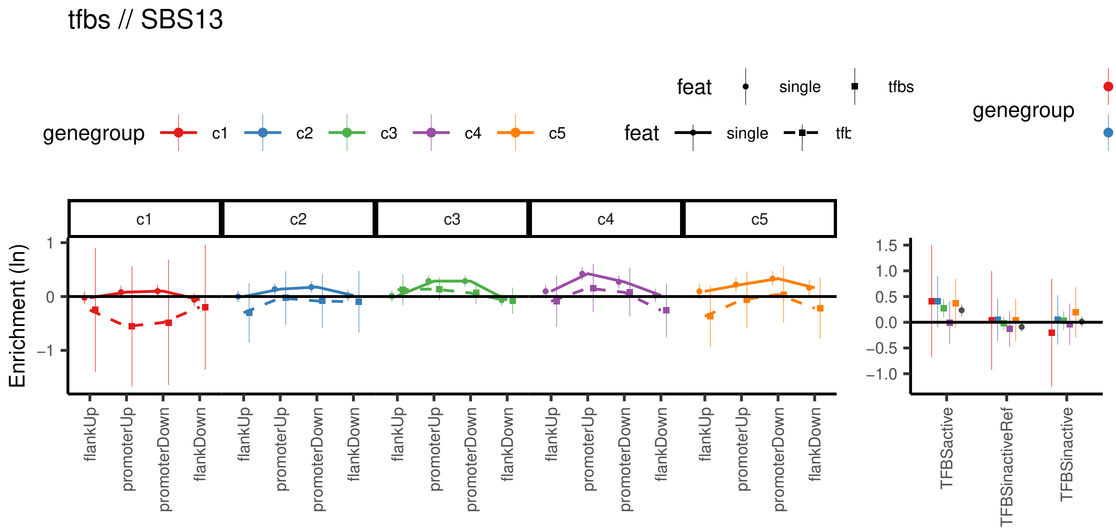

tfbs // SBS13
feat
single
tfbs
genegroup
c1
c2
c3
c4
c5
c1
c2
c3
c4
c5
Enrichment (ln)
1
0
−1
flankUp
promoterUp
promoterDown
flankDown
1.5
1.0
0.5
0.0
−0.5
−1.0
TFBSactive
TFBSinactiveRef
TFBSinactive

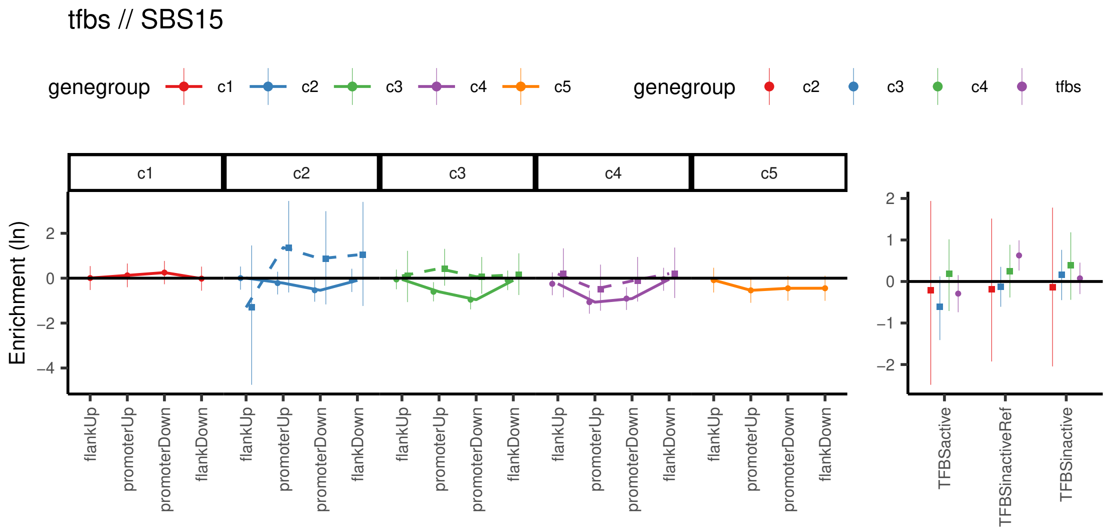
tfbs // SBS15
genegroup
c1
c2
c3
c4
c5
genegroup
c2
c3
c4
tfbs
c1
c2
c3
c4
c5
Enrichment (ln)
2
0
−2
−4
flankUp
promoterUp
promoterDown
flankDown
2
1
0
−1
−2
TFBSactive
TFBSinactiveRef
TFBSinactive

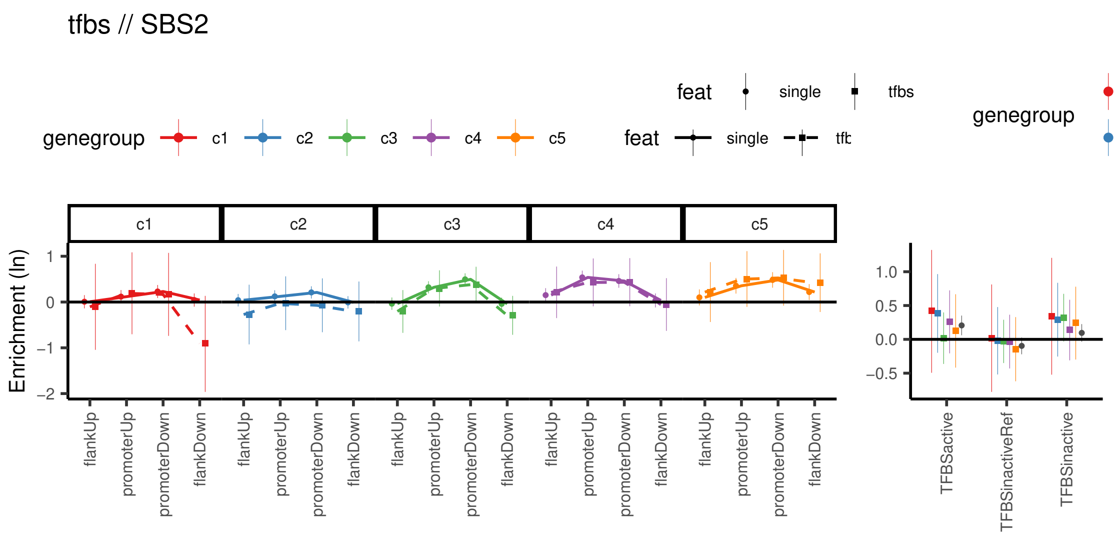

tfbs // SBS2
feat
single
tfbs
genegroup
c1
c2
c3
c4
c5
feat
single
tfb
genegroup
c1
c2
c3
c4
c5
Enrichment (ln)
1
0
−1
−2
flankUp
promoterUp
promoterDown
flankDown
1.0
0.5
0.0
−0.5
TFBSactive
TFBSinactiveRef
TFBSinactive

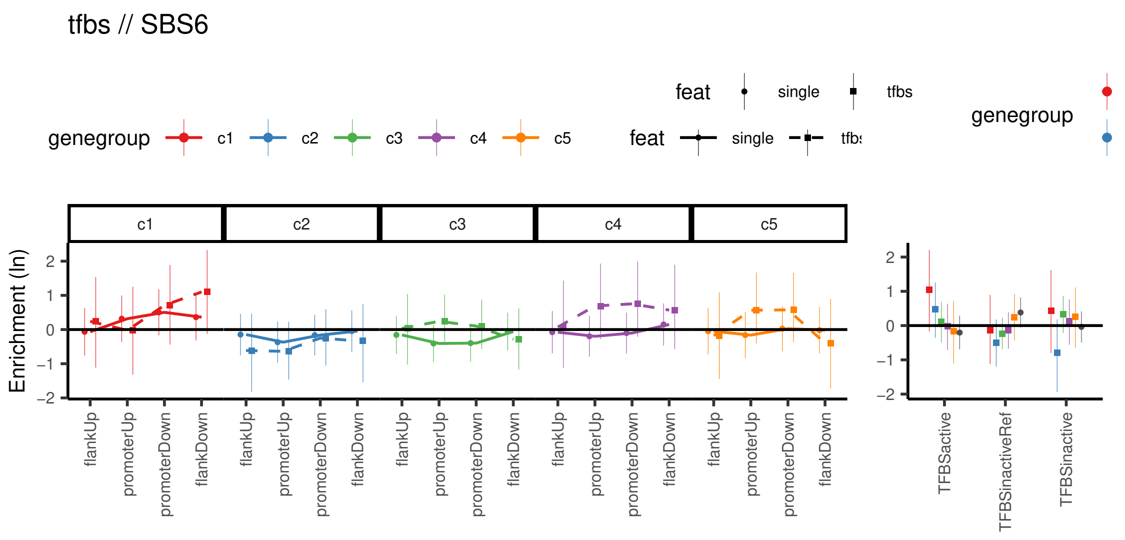
tfbs // SBS6
feat
single
tfbs
genegroup
c1
c2
c3
c4
c5
feat
single
tfbs
genegroup
c1
c2
c3
c4
c5
Enrichment (ln)
2
1
0
−1
−2
flankUp
promoterUp
promoterDown
flankDown
2
1
0
−1
−2
TFBSactive
TFBSinactiveRef
TFBSinactive

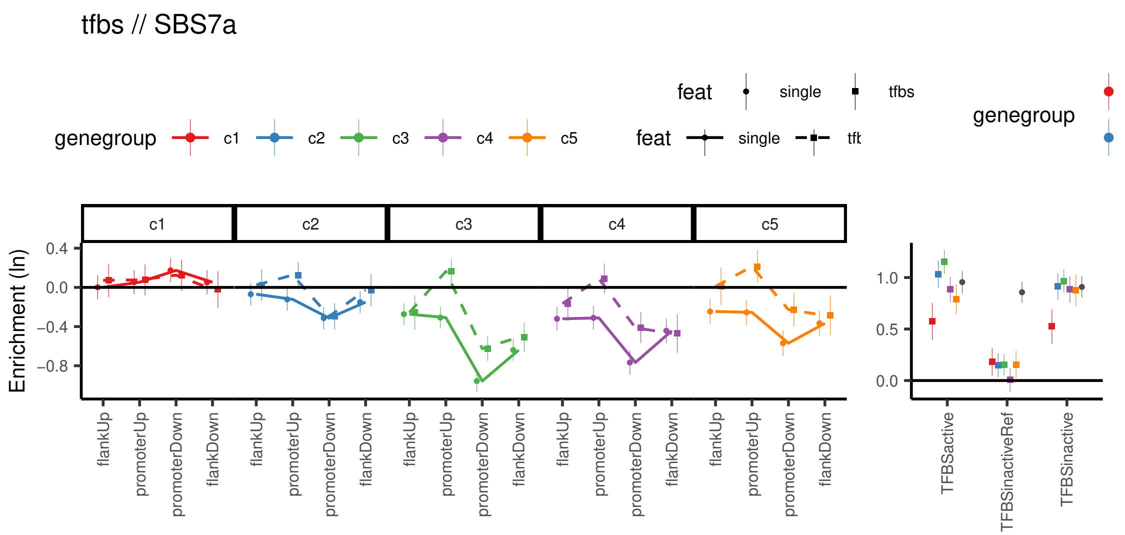

tfbs // SBS7a
feat
single
tfbs
genegroup
c1
c2
c3
c4
c5
Enrichment (ln)
0.4
0.0
−0.4
−0.8
flankUp
promoterUp
promoterDown
flankDown
1.0
0.5
0.0
TFBSactive
TFBSinactiveRef
TFBSinactive

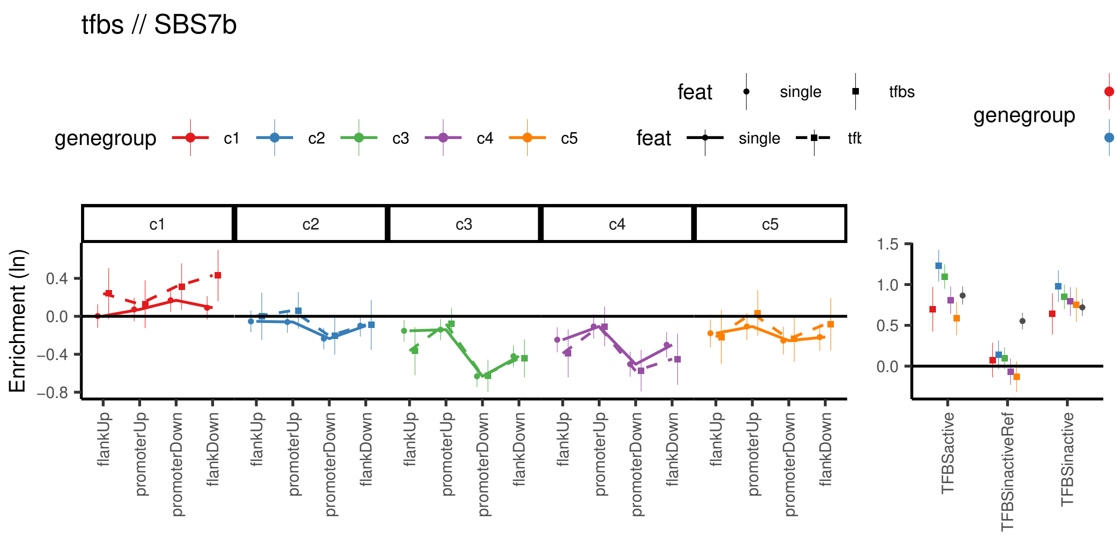

tfbs // SBS7b
feat
single
tfbs
genegroup
genegroup
c1
c2
c3
c4
c5
feat
single
tfb
c1
c2
c3
c4
c5
Enrichment (ln)
0.4
0.0
−0.4
−0.8
flankUp
promoterUp
promoterDown
flankDown
1.5
1.0
0.5
0.0
TFBSactive
TFBSinactiveRef
TFBSinactive
