# Supplementary figures and images for: Mutation rate heterogeneity at the sub-gene scale due to local DNA hypomethylation
Source: Nucleic Acids Res. 2024 Apr 8;52(8):4393–408. doi: 10.1093/nar/gkae252 (PMC11077091; doi:10.1093/nar/gkae252)

# PC1 coordinates

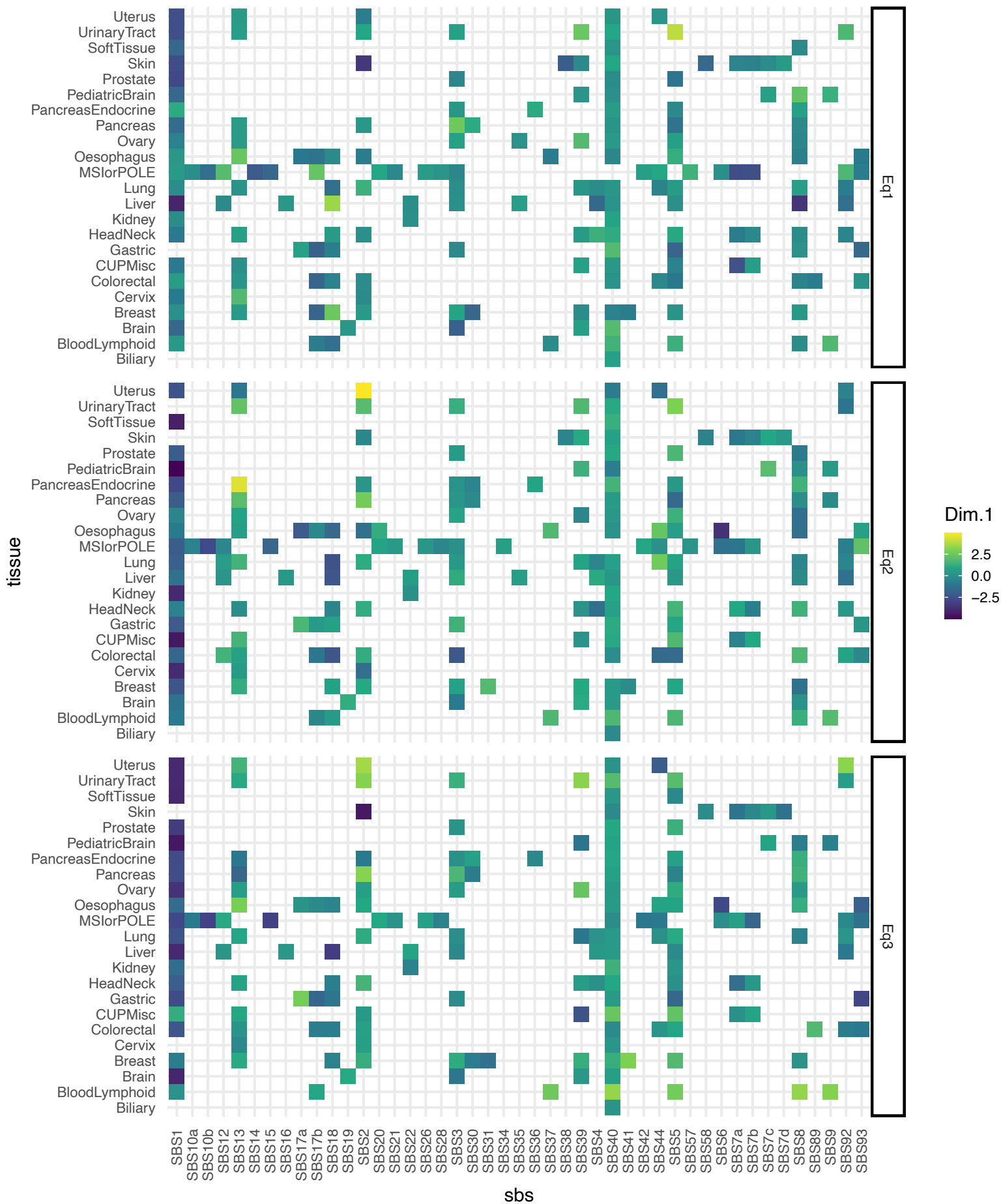

# PC2 coordinates

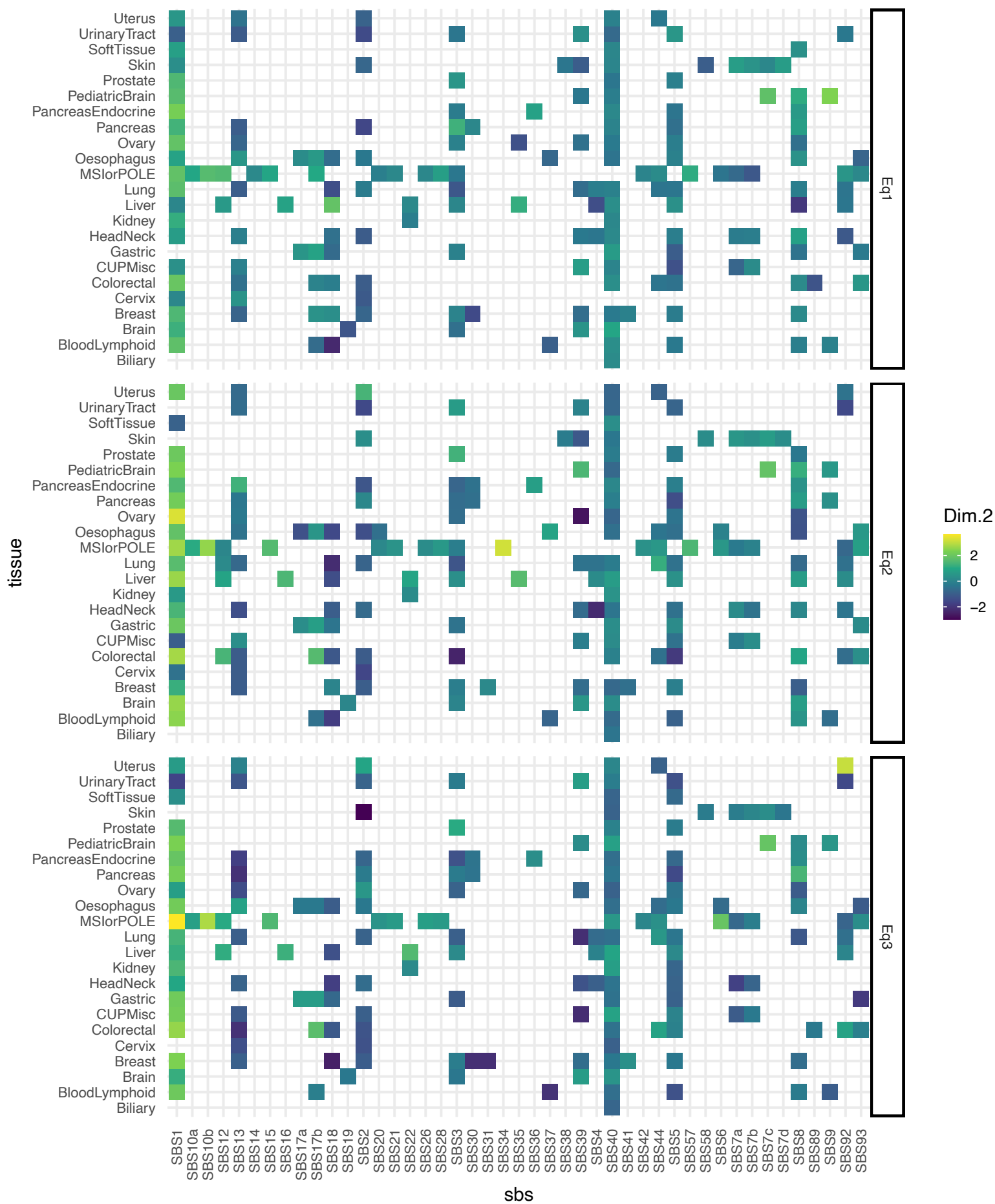

# PC3 coordinates

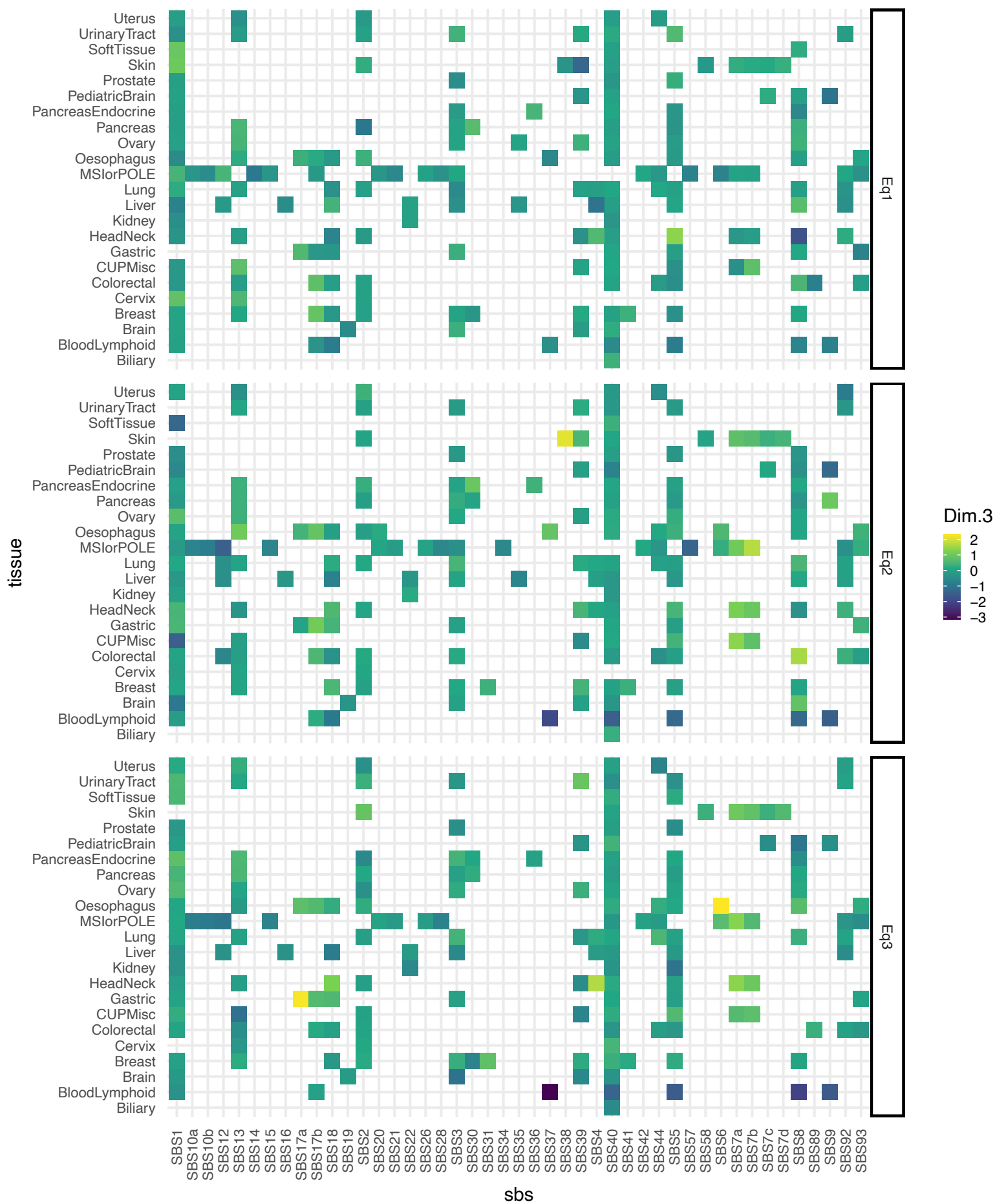

Supplement: gkae252_Supplemental_Files [file gkae252_supplemental_files.zip › Supplementary_Figure_S2.pdf]
